# DNA Topoisomerase 1 Structure-BASED Design, Synthesis, Activity Evaluation and Molecular Simulations Study of New 7-Amide Camptothecin Derivatives Against *Spodoptera frugiperda*

**DOI:** 10.3389/fchem.2018.00456

**Published:** 2018-10-05

**Authors:** Zhiyan Jiang, Zhijun Zhang, Gaofeng Cui, Zhipeng Sun, Gaopeng Song, Yingqian Liu, Guohua Zhong

**Affiliations:** ^1^Key Laboratory of Natural Pesticide and Chemical Biology, Ministry of Education, and Key Laboratory of Crop Integrated Pest Management in South China, Ministry of Agriculture, College of Agriculture, South China Agricultural University, Guangzhou, China; ^2^College of Materials and Energy, South China Agricultural University, Guangzhou, China; ^3^School of Pharmacy, Lanzhou University, Lanzhou, China

**Keywords:** camptothecin, synthesis, topoisomerase 1, binding mode, molecular docking, molecular dynamics simulation, activity

## Abstract

Camptothecin and its derivatives (CPTs) have strong toxicity to eukaryotic cells by targeting their DNA topoisomerase 1 (Top1) protein and have been increasingly explored as potential pesticides for plant protection. However, the detailed structure-binding mechanism of the interactions between CPTs and the insect Top1 protein remains unclear, which significantly hinders the development of novel CPTs as new insecticides. Herein, a series of 7-amide camptothecin analogs based on the binding mode of camptothecin in complex with Top1 (*Sf* Top1)-DNA from *Spodoptera frugiperda* cultured cell line Sf9 were designed and synthesized. Fifteen of these compounds exhibited excellent cytotoxic activity (values of IC_50_ from 2.01 to 6.78 μM) compared with camptothecin (29.47 μM). The molecular simulations revealed the binding mechanism when the camptothecin parent rings were inserting parallel to DNA bases and stabling the ternary complex by π-π stacked and hydrogen-bond interactions, and further suggested that introduction of lipophilic and some electron-withdrawing groups on the amide linkage of camptothecin could be beneficial to its activity via some non-covalent interactions. Furthermore, almost all the synthesized compounds could inhibit the growth of *Spodoptera litura* larvae strongly (Inhibition rate from 50.20 to 79.05%), superior or comparable to camptothecin (55.69%) after 8 days of exposure. In particular, the compounds **4c**, **4d**, **4f**, and **4j**, which presented more than 70% inhibitory activities, were deserved to be developed as potential biorational pesticides. The information described here would be useful for the further design and development of potentially effective pesticides in the field of plant protection.

## Introduction

In recent years, much interest has focused on the development of biorational pesticides with good insecticidal activity and environmental safety. Many active compounds derived from plants, such as azadirachtin, rotenone and nicotine, not only possess superior activity and environmental safety, but also provide lead compounds for biorational pesticides innovation (Petroski and Stanley, 2009; Cantrell et al., 2012). Camptothecin, a natural quinolone alkaloid product, was extracted from the bark of the Chinese tree *Camptotheca acuminata* by Wall et al. in the early 1960s (Wall and Wani, 1966). During the 1980s, topoisomerase 1 (Top1) has been identified as the target protein of camptothecin (Hsiang et al., 1985). Top1 is involved in the process of cell growth and proliferation, and is needed for DNA replication and transcription (Staker et al., 2002). Its mode of action is to break one of DNA supercoiling double-strands by covalent binding, then making a recombination with the other strand revolving around (Pommier et al., 1998; Redinbo et al., 1998). As an inhibitor, camptothecin could prevent DNA re-ligation by forming a stabilizing ternary composite with Top 1-DNA (Staker et al., 2002). Due to the special mechanism, CPTs present potent anti-tumor or insecticidal activities, and some of them have been commonly used in clinical trials (Broom, 1996; Chabot, 1997). Meanwhile, camptothecin was also used as a potent chemosterilant against the house fly for decades (DeMilo and Borkovec, 1974; Borkovec, 1976). In recent years, various CPTs have been synthesized and exhibited efficient toxicity to S*podoptera exigua, Nilaparvata lugens, Brevicoryne brassicae, Chilo suppressalis, Tetranychus cinnabarinus, Bursaphelenchus xylophilu*, and so on (Ma et al., 2010; Wu et al., 2015; Wang et al., 2017). It has been evaluated that 7-substituted analogs demonstrated quite good insecticidal activity (Liu et al., 2010; Ma et al., 2010; Wu et al., 2015). Moreover, camptothecin and its mixtures with other effective ingredients exhibited good insecticidal or synergistic activity when were evaluated in the laboratory and field against various insect pests (Sun et al., 2012; Tong and Feng, 2016a,b). Previous works conducted in our lab suggested that camptothecin could induce apoptosis in *Spodoptera frugiperda* cultured cell line Sf9, and inhibits the Top1 of insect cells by decreasing the ability to relax the negatively supercoiled plasmid pBR322 DNA (Wang et al., 2011; Gong et al., 2014). Similarly, some studies have also shown an identical outcome, which further revealed the key residues involved in the relaxation activity and the apoptosis mechanism related to the mitochondrial pathway (Zhang et al., 2012, 2013, 2017; Ren et al., 2017).

In order to facilitate the development of new compounds, rational drug design combined with structural biology is the most appropriate potential method (Hoque et al., 2017). Although the crystal structure of human Top1 in complex with the poison topotecan and a covalent complex with DNA duplexes was established in 2002 (Staker et al., 2002), few of researchers has addressed the importance of using this structure to develop new compounds applicable for pest management. Here we cloned the Top1 (*Sf* Top1) gene from Sf9 cells, built the 3D model for *Sf* Top1 by homologous modeling, and then illuminated its binding mode with CPTs. We designed and synthesized quite a number of target derivatives based on the binding mode, and assessed the activities *in vitro*. Furthermore, molecular docking and dynamical simulations studies were applied to clarify the interaction mechanism between CPTs and *Sf* Top1-DNA complex. Finally, the CPTs' activities against *S. litura* have also been tested *in vivo*. All these studies would redound to develop CPTs as new biorational pesticides.

## Materials and methods

### Cell culture

*S. frugiperda* cultured cell line Sf9 was maintained at 27°C in Grace's insect cell culture medium (Gibco, USA) with fetal bovine serum (FBS, Gibco, USA) (10%) added under a CO_2_ atmosphere (5%) and contained in 25 cm^2^ culture flasks (Corning, USA). The cultures were sub-cultured every 3 days.

### Isolation of *Sf*Top1 gene

Total RNA was extracted from Sf9 cells using the total RNA isolation system kit (Biotech, China) according to the manufacturer's instructions. The isolated RNA was reversely transcribed to first-strand cDNA with reverse transcriptase M-MLV (TaKaRa, China) following the manufacturer's instructions. The cDNA was used as a template for a polymerase chain reaction (PCR) following a general protocol. The forward prime (5′-ATGAGTGTCGAAAATCCC-3′) and the reverse prime (5′-TTAGAAGATATATTCCGGACC-3′) were designed according to the transcriptome sequencing data (Shu et al., 2017) and applied to the amplification of the entire coding region of the *Sf* 9Top1 gene. The gene targeting was carried out using Primerstar HS DNA Polymerase (Takara, Dalian, China), with an initial denaturation step of 98°C for 3 min and followed by 35 cycles of 98°C for 10 s, 55°C for 5 s and 72°C for 3 min. A final step for 10 min at 72°C was used to extend the amplicons fully. The PCR products were checked on a 1.2% agarose gel and purified using a Gel Extraction Kit (Omega, Norcross, GA). The target DNA was ligated into the pMD19-T vector (TaKaRa) and sequenced by the Invitrogen Company (Guangzhou, China).

### *In vitro* toxicity assay

#### Cell proliferation assay

The cytotoxicity of camptothecin (purchased from Nanjing Xyumax Bio-Tech Co., Ltd., Nanjing, Jiangsu, China) and the synthesized compounds against Sf9 cells were evaluated using the MTT assay. The exponentially grown Sf9 cells were collected and then diluted with fresh culture medium. Cells (100 μL, 2 × 10^3^ per well) was seeded into 96-well plates and incubated at 28°C for 24 h in a gas atmosphere of CO_2_ (5%). The test compounds were then sequentially added to the culture plates at five concentrations, with the untreated cells as the control. All of the treatments and controls were repeated three times. After incubation at 28°C for 24 h, MTT solution (50 μL, 1 mg mL^−1^) was added to each well and incubated for a further 4 h at 28°C. The culture medium was discarded carefully, and DMSO (150 μL) was then added to each well to dissolve the formazan crystals. The absorbance of each well was measured at 490 nm using an Enzyme-Linked Immunosorbent Assay (ELISA) reader (Bio-Tek, USA). The anti-proliferation activity was calculated according to the following formula:

$$\begin{array}{l} \text{Cell proliferation inhibition rate }\left(\text{\%}\right)=\left(\text{O}{\text{D}}_{\text{control}}-\text{O}{\text{D}}_{\text{treatment}}\right)\\ \times 100/\text{O}{\text{D}}_{\text{control}}. \end{array}$$

#### Recombinant protein expression and purification

The *Sf* Top1 encoding the residues 367-929 (*Sf* Top70) was amplified by PCR with the forward prime (5′-CCCGAATTCGAAGTTTGGAAATGGTGGGAAG-3′) and the reverse prime (5′- CAAGTCGACGAAGATATATCCGGACCGGCCAT−3′), and then cloned into the vector pEASYBLunt E2 to generate plasmid pEASY-*Sf* Top70. *E. coil* BL21 (DE3; TIANGEN biotech, China) cells were transformed with pEASY- *Sf* Top70 grown at 37°C in LB medium including 100 mg/mL ampicillin overnight. Exponentially growing bacteria (OD600 = 0.6) were treated with 0.5 mM isoproyl-1-thio-b-D-galactopyranoside (IPTG) to induce target protein expression for 4 h at 30°C. The culture *E. coli* with recombinant plasmid was collected by centrifugation at 7,800 rpm for 5 min and then washed with the PBS buffer twice. The cells were resuspended in lysis buffer (500 mM Tris–HCl, pH 7.5, 250 mM KCl, 0.5% Triton X-100, 1 mM DTT, 2 mM EDTA, 1% protease inhibitors and 0.01% nuclease) with a final concentration of 250 μg/mL lysozyme on ice for 30 min. Cell debris and insoluble materials were removed by centrifugation at 12,000 rpm for 30 min at 4°C. The collected supernatant was purified with HisPur™ Ni-NTA Resin (ThermoFisher, China) according to the manufacturer's instructions. The purified products confirmed by SDS-PAGE were kept in 50% glycerol with aliquots at −80°C.The concentration of protein was detected by Bradford's method.

#### Effects of CPT and derivatives pre-treatment on the specific activity of SfTop1

The purified recombinant protein was used for the specific activity analysis. The insect DNA topoisomerase-1 relaxation assay was performed according to the procedures as previously reported with some modifications (Zhang et al., 2013). The mixed total reaction buffer including 1 μL 10 × buffer (10 mM Tris–HCl, pH 7.5, 150 mM KCl, 1 mM DTT, 1 mM EDTA and 1 mg/mL BSA), 20 ng purified protein, 250 ng plasmid PBR322 (TaKaRa, China), 1 μL DMSO or CPTs (final concentration was 100 mM), and the supplemental water in 10 μL volumes was incubated under 37°C for 30 min. The reaction was terminated by adding proteinase K (250 μg/mL) and 0.5% SDS. Subsequently, the different forms of DNA were detected by electrophoresis in 1% agarose gel under 100 V for 50 min.

### Homology model

The reported X-ray crystal structure (PDB code: 1K4T, www.rcsb.org/) of *Hs*Top1 was used as the template structure for building the model structure of *Sf* Top1. The homology modeling module in Discovery Studio 2017 (DS 2017) (Dassault Systèmes BIOVIA, 2016) was employed to build the *Sf* Top1 structure. The sequence of *Sf* Top1 was first aligned to the template protein based on their structural similarity using the structure alignment method in the MODELER program (Sali and Blundell, 1993). The nucleic acid chains and ligand were copied from the template structure, and the number of models with a high optimization level was set to 20. Other parameters were set to the default value. Furthermore, the structure that had the lowest probability density function (PDF) energy (Sali and Blundell, 1993) and DOPE score (Shen and Sali, 2006) was selected as the candidate model and optimized by conjugate gradient (Press et al., 1987) to remove the steric overlap that produces bad contacts. The final model was further assessed using the Verify Protein (Profiles-3D) and Ramachandran Plot protocol (Lovell et al., 2003).

### Molecular simulations

The *Sf* Top1-DNA structure model was prepared using the Prepare Protein protocol. The binding site was defined from the current ligand. The target compounds were minimized using the CHARMm forcefield with 2000 steps of the steepest descent algorithm at an RMS gradient of 0.1 kcal/(mol × Å) and followed by using 2000 steps of the conjugate gradient at an RMS gradient of 0.01 kcal/(mol × Å). All of the minimized compounds were docked into the binding site using a CHARMm-based CDOCKER protocol (Wu et al., 2003) in DS 2017. In this process, 30 random conformations were generated in high temperature (1000 K) with a dynamics step of 1000 and translated into the binding site. The docking results were then displayed as non-bond interactions between the receptor and ligands. The best scoring pose was selected for further analysis. The binding free energy (Tirado-Rives and Jorgensen, 2006) was estimated between each ligand and receptor by using CHARMm based implicit solvation models. The pharmacophore model was also constructed based on the binding model of camptothecin under the Pharmacophore Generation protocol in DS2017.

The best-scoring configuration of *Sf* Top1-DNA in complex with the ligand **4j** was selected for molecular dynamics simulation (MDs). The AMBER ff03 force field (Duan et al., 2003) implemented by Sorin and Pande (2005) in the GROMACS MD package (Pronk et al., 2013) version 2016.4 was used as the parameters for proteins and ions. Meanwhile, the general AMBER force field (GAFF) (Wang et al., 2004) and AMBER ff03 force field were used as the parameters for the ligand and nonstandard residues using parmchk2 program in AmberTools18 (Case et al., 2018). In order to obtain the partial atomic charges of the compound and nonstandard residues, the stable structure was determined through the geometry optimization at the HF/6-31g(d) level of theory with Gaussian09 program (Frisch et al., 2009). Subsequently, the electrostatic potential (ESP) charges were calculated with the same method and basis set, followed by the restrained electrostatic potential (RESP) fitting (Cieplak et al., 1995). The topologies have been created using tleap with the AMBER ff03 force field, and have been then converted in the GROMACS format using Acpype (Sousa da Silva and Vranken, 2012). The complex have been immersed in a rhombic dodecahedron box and further solvated with TIP3P water molecules (Jorgensen et al., 1983), and then neutralized by adding Na+ counter-ions via the genion tool of the GROMACS package. The final super script system being composed by 154,134 atoms. The simulations have been then carried out using Gromacs. Electrostatic interactions have been taken into account by means of the Particle Mesh Ewald method (Cheatham et al., 1995) and the bond lengths and angles have been constrained using the LINCS algorithm (Ryckaert et al., 1977). The system has then been simulated for 50 ns with a time step of 2.0 fs at a constant temperature of 300 K using the Berendsen's method (Berendsen et al., 1984) with a coupling constant of 0.1 ps during sampling, while pressure was kept constant at 1 bar using the Parrinello-Rahman barostat (Parrinello and Rahman, 1981) with a coupling constant of 1.0 ps during sampling. Standard analyses, as root mean square deviations (RMSD) and fluctuations (RMSF) evaluations were calculated using tools of GROMACS package. Images were produced with VMD (Humphrey et al., 1996), Pymol (Delano, 2002) version 1.7 (http://www.pymol.org) and DS 2017, and graphs have been obtained with the Grace program (http://plasma-gate.weizmann.ac.il/Grace/).

### Bioassay

*Spodoptera litura* was fed in the lab by a standard condition, and was assayed with the synthesized compounds according to previous research with some modifications (Sang et al., 2016). Newly hatched 2rd larvae were used to determine the inhibiting rate in the surviving larval weight increase test. Target compounds were dissolved in DMSO (10 mg mL^−1^), and followed by dilution with acetone: water (1:1) to a final concentration of 0.02 mg mL^−1^. An acetone: water solution containing DMSO (0.2%) was used as the negative control. Fifteen fresh taro dishes (2 cm in diameter) were dipped in the target solution for 10 s, followed by air-drying and were then transferred into a Petri dish containing 20 starved larvae. All the treatments and controls were repeated in triplicate, and the tested larvae were cultured at a temperature of 25 ± 2°C and 60–70% relative humidity with a photoperiod of 16:8 h (light: dark). After every 2 days of exposure, all of the existing taro dishes were replaced with fresh leaves that had undergone the same treatment. The larvae weight increase (LWI) after treatment for 2, 4, and 8 days were calculated respectively, and the inhibition rate (IR) of weight increase was reported according to the following formula (Zhong et al., 2001):

$$\begin{array}{l} IR\;\left(\%\right)=\left(LWI_{\text{control}}-LWI_{\text{treatment}}\right)\;\times \;100/LWI_{\text{control}.} \end{array}$$

### General chemical methods

All chemicals and reagents used in the current study were of analytical grade. The reactions were monitored by thin layer chromatography (TLC) on precoated E. Merck silica gel 60 F254 plates. Flash column chromatography was performed on silica gel (200–300 mesh, Qingdao Haiyang Chemical Co., Ltd., Qingdao, China). ^1^H NMR and ^13^C NMR spectra were taken on a JEOL JNM-ECP 600 spectrometer with tetramethylsilane as an internal standard, and chemical shifts are recorded in ppm values. Mass spectra were recorded on a Q-TOF Global mass spectrometer.

### Chemical synthesis

#### (S)-4-ethyl-4-hydroxy-3,14-dioxo-3,4,12,14-tetrahydro-1h-pyrano[3′,4′: 6,7]indolizino[1,2-b]quinoline-11-carboxylic acid (3)

The synthesis methods for 7-carboxy camptothecin (**3**) have been described previously (Ricci et al., 2011). Briefly, to obtain 7-dimethoxmethyl camptothecin (**1**), the starting CPT was suspended in methanol (1.5% solution, w/v) and 96% H_2_SO_4_ (10%, v/v), and then two oxidizing steps were performed, firstly with an oxidizing system consisting of 30% H_2_O_2_/iron sulfate and secondly with an oxidizing system consisting of MnO_2_. Subsequently, 7-Formyl camptothecin (**2**) was obtained by reflux with an acetic acid/water mixture. Afterwards, the target product was obtained using an oxidizing system consisting of 30% H_2_O_2_/formic acid and identified by using ^1^H NMR and ^13^C NMR spectra. ^1^H NMR (600 MHz, DMSO-*d*_6_): δ 14.58 (s, 1H, COOH), 8.88 (d, *J* = 8.6 Hz, 1H, Ar–H), 8.24 (d, *J* = 8.4 Hz, 1H, Ar–H), 7.92 (t, *J* = 7.6 Hz, 1H, Ar–H), 7.81 (t, *J* = 7.7 Hz, 1H, Ar–H), 7.36 (s, 1H, Ar–H), 6.57 (s, 1H, OH), 5.44 (s, 2H, CH_2_), 5.41 (s, 2H, CH_2_), 1.87 (m, 2H, C*H*_2_CH_3_), 0.89 (t, *J* = 7.3 Hz, 3H, CH_2_C*H*_3_). ^13^C NMR (151 MHz, DMSO*-d*_6_) δ 172.76, 166.81, 157.12, 153.46, 150.46, 149.51, 145.40, 131.82, 131.43, 130.86, 130.22, 129.36, 126.36, 125.21, 120.01, 97.21, 72.83, 65.75, 52.43, 30.92, 8.21.

#### General procedure for synthesis of compounds 4a–q

7-Carboxy camptothecin **3** (80 mg, 0.204 mM) was added to dry DMF (4 mL) under ultrasonic conditions. The suspension was incubated at −20°C and then 41 mg HOBt (0.306 mM, 1.5 eq) and 47 mg 1-Ethyl-3-(3-dimethylaminopropyl) carbodiimide hydrochloride (EDCI, 0.245 mM, 1.2 eq) was added, and the resulting mixture was stirred for 1 h. Subsequently, various amines (0.245 mM, 1.2 eq) were added slowly to the solution, respectively, and the solutions were stirred at room temperature for 23 h. All of the reactions were protected under nitrogen and monitored by TLC (eluent CH_2_Cl_2_: MeOH). The reaction was terminated with water (1 mL), and the solvent was removed *in vacuo*. Afterwards, the residues were further purified by silica gel flash chromatography to obtain the target products. These compounds were identified by ^1^H NMR, ^13^C NMR, and HRMS spectra additionally, and the results are shown in the Supplementary Figures 5–38.

## Results

### Characterization of *Sf*Top1

**Figure 1:**
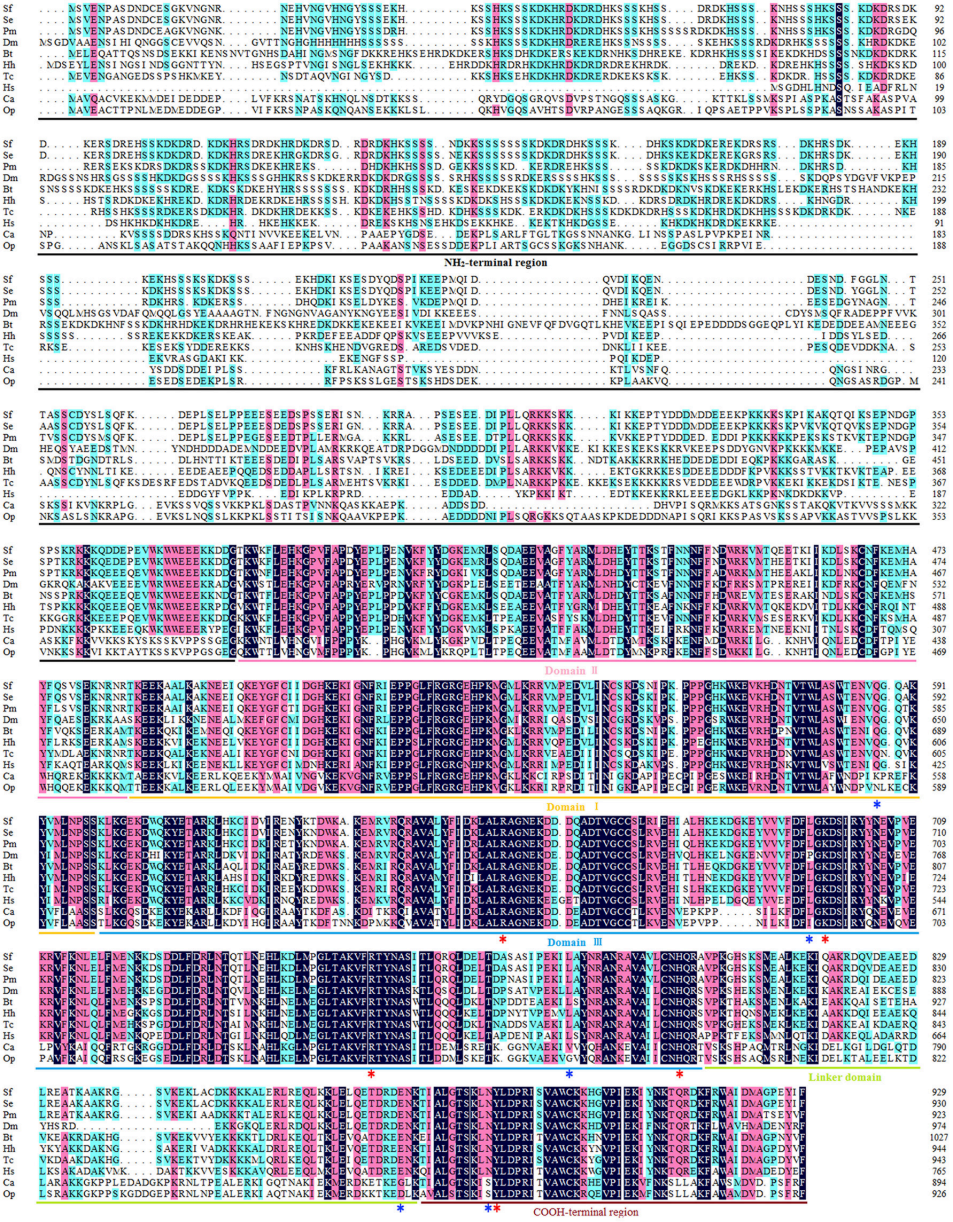
Multiple sequence alignment of Top1. The amino acid sequences of Top1 proteins from nine species were obtained from the website of the National Center for Biotechnology Information (NCBI, https://www.ncbi.nlm.nih.gov/) and were aligned using DNAMAN with appropriate parameters. NH_2_-terminal region, aa 1–380 (numbered according to *Hs*Top1); Domain II, aa 381–485; Domain I, aa 486–599; Domain III, aa 600–800; Linker domain, aa 801–876; COOH-terminal region, aa 877–929. The red asterisk represents the crucial residue for catalytic activity, and the blue asterisk represents the key residue involved in camptothecin-resistance with a camptothecin direct/indirect binding. Sf, *Spodoptera frugiperda*; Se, *Spodoptera exigua* (GenBank ID: JN258956); Pm, *Papilio machaon* (GenBank ID: XP_014361563); Dm, *Drosophila melanogaster* (GenBank ID: NM078606); Bt, *Bombus terrestris* (GenBank ID: XP_003398903); Hh, *Halyomorpha halys* (GenBank ID: XP_014284042); Tc, *Tribolium castaneum* (GenBank ID: XP_008200441); Hs, *Homo sapiens* (GenBank ID: AH003017.2); CA, *Camptotheca acuminate* (GenBank ID: AB372511); Op, *Ophiorrhiza pumila* (GenBank ID: BAG31373.1).

The open reading frame (ORF) of the Top1 gene consists of 2,787 base pairs, and the deduced Top1 polypeptide has 929 amino acids with a calculated molecular weight of 108.04 kDa and an estimated pI of 8.91. Multiple sequence alignment was performed to explore the similarities and differences of the protein amongst nine species. It exhibited high homology (58.6% identity) with all of the species mentioned in Figure 1. In particular, it shared an extremely high homology level (98 and 84% identity) with the Lepidoptera insect *S. exigua* and *Papilio machaon*, and also showed more than 51% identity with *Homo sapiens* and other insects. It is noteworthy that *Sf* Top1 has a junior homology level (34 and 35% identity, respectively) with *C. acuminate* and *O. pumila* presenting strong resistance to its product camptothecin through mutants of their amino acid residues. Mutants such as Q421K, L530I, L617G, E710G, and N722S, numbered according to *Hs*Top1, are involved in the camptothecin direct/indirect binding and have been reported to be responsible for the production of anti-camptothecins in plants (Sirikantaramas et al., 2008, 2015). Interestingly, some tumor cells mutate themselves to adapt the oppression of anti-tumor drugs such as camptothecin, and some frequent mutations, such as F361, G363, R364, G503, D533, G717, N722, T729, give them significant resistance (Gongora et al., 2008). In general, insect cells and normal human cells are more sensitive to camptothecin, and we did not find any of the reported mutations leading to camptothecin resistance in their sequences, which may explain why the insect cells are more sensitive to camptothecin derivatives.

Consistent with other eukaryotes, the *Sf* Top1 protein is composed of four different structural domains (Figure 1): the NH_2_-terminal region, the core domains contained three subdomains I~III, the linker domain and the COOH-terminal region (Redinbo et al., 1998). Similar to *H. sapiens*, the core domains and the COOH-terminal region, which contains all of the catalytic residues (R488, K532, R590, H632 and Y723; numbered according to *Hs*Top1) presenting a crucial role in catalytic activity, are highly conserved in *SfTop1*. In addition, two other regions (NH_2_-terminal and linker domain) are poorly conserved and not strictly required for the catalytic and relaxation functions, while the short linker domain has a function of connecting the core and COOH-terminal domains (Redinbo et al., 1998).

### Building a 3D model of *Sf*Top1 with nucleic acid

**Figure 2:**
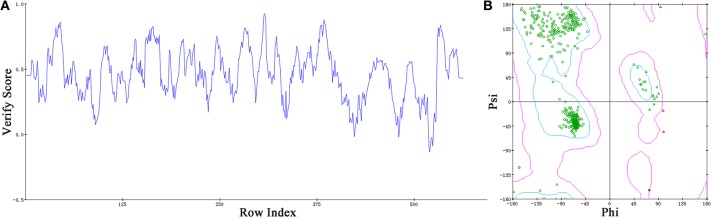
Assessment of the *Sf* Top1 protein model with two different methods of Verify Protein (Profiles-3D) **(A)** and Ramachandran Plot **(B)**. **(A)** The Verify scores of per-residue were calculated to evaluate the quality of the theoretical model. The score value, which is greater than or equal to zero, indicates that the residue is fit to its current 3D environment. **(B)** The Ramachandran Plot provides a graphical representation of the local backbone conformation of each residue in a protein. Each point on the Ramachandran Plot represents the ϕ (phi) and ψ (psi) torsion angles of a residue. The green circles or triangles indicate that these amino acids residues are acceptable.

**Figure 3:**
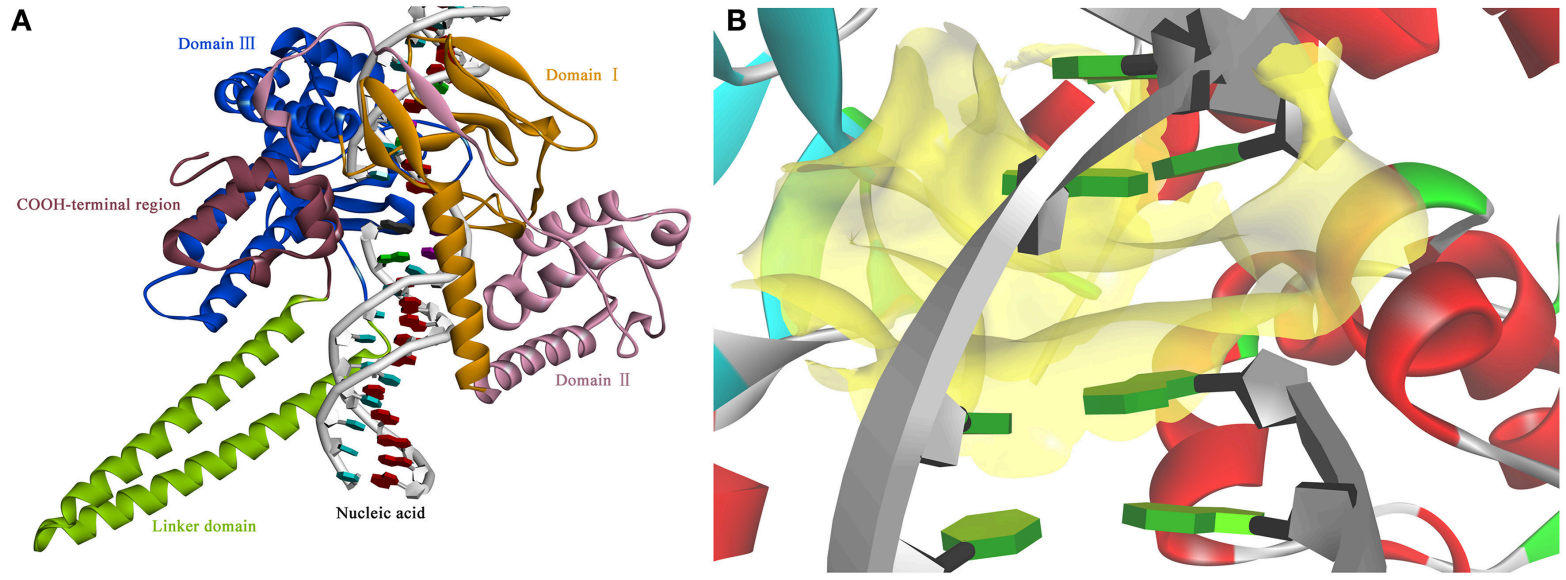
The view of 3D structure of the *Sf* Top1-DNA complex **(A)** and the binding cavity **(B)**. **(A)** The complex presented five domains in the protein with different colors: Domain II (purple), Domain III (blue), Domain I (yellow), Linker domain (green) and COOH-terminal region (brown). **(B)** The center of the binding cavity (yellow) embed in the *Sf* Top1-DNA complex.

Based on the information mentioned above, the crystal structure of *Hs*Top1 (PDB: 1K4T) was employed as the reference to building the corresponding three-dimensional structure via a homology modeling module MODELER in DS 2017 with standard parameters. The sequence similarity of *Sf* Top1 catalytic regions with *H. sapiens* reached 84.6%, which implied that a reasonable model could be built by this template. The model with the lowest PDF, and having a total energy (Sali and Blundell, 1993) of 2169.6 and a DOPE score (Shen and Sali, 2006) of −60429.9 was selected and optimized. The final structure was further assessed via the Verify Protein (Profiles-3D) and the Ramachandran Plot protocol to measure the compatibility of an amino acid sequence with a 3D protein structure and verify the predicted torsion angles in the protein (Lüthy et al., 1992; Lovell et al., 2003). The verify score of this model reached a value of 236.2, which approximated to the Verify Expected High Score of 257.5. The line plot of the per-residue Verify scores and the graphic of the Ramachandran Plot (Figure 2) showed that more than 99% of amino acid residues in the model were reasonable. When matched to the corresponding sequence, the 3D structure of *Sf* Top1 has three core subdomains (Domain I~III), a linker region and COOH-terminal as shown in Figure 3, and the cavity that appears to be adequate space to accommodate seemingly possible inhibitors. These results suggested that the model would be appropriate for further study.

### Designing camptothecin derivatives

**Figure 4:**
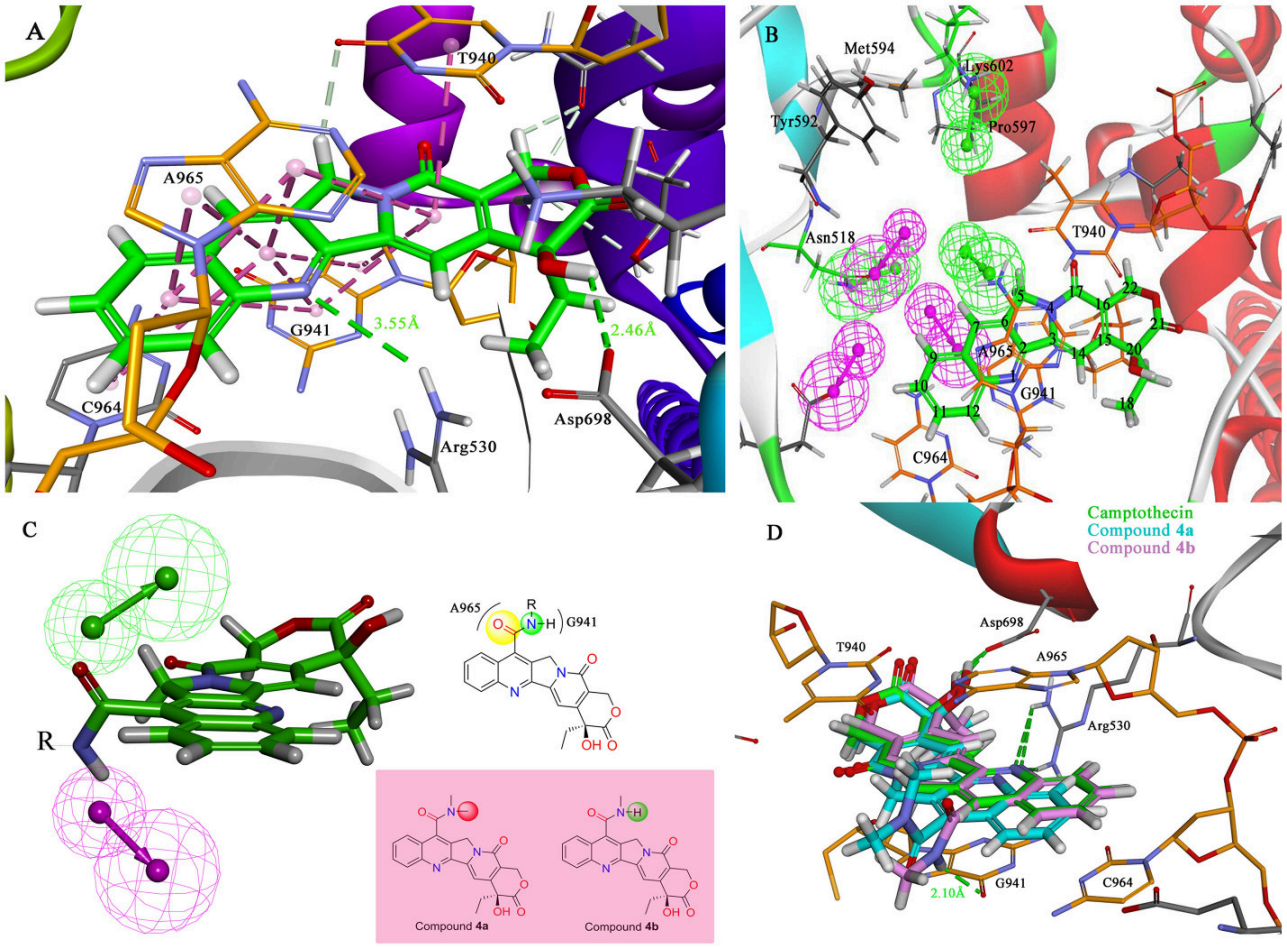
Analysis of the binding model of camptothecin with *Sf* Top1-DNA complex and designation of the compounds based on the model structure. **(A)** The docked mode of camptothecin (green) with *Sf* Top1-DNA complex. The bases of DNA are shown with orange carbons. The H-bonds formed between the ligand and residues are indicated as green dashed lines, and the π-π stacked interactions are shown in lavender dashed lines. **(B)** The pharmacophore model closest to the 7-C of camptothecin in the center of the *Sf* Top1-DNA complex binding site. The larger green and purple mesh spheres represent the hydrogen bond donor and acceptor onto the protein or nucleic acid, respectively. In contrast, the smaller green sphere represent the hydrogen bond acceptor, and the purple one represents the hydrogen bond donor. **(C)** The assumed model of the amide-based camptothecin substituents interacting with the base pairs, and the structures of designed compound **4a** and **4b**. **(D)** The docked binding models of camptothecin (green), compound **4a** (cyan) and **4b** (lavender).

The molecular docking process was performed through a standard CDOCKER protocol, which is a classical method using a CHARMm-based molecular dynamics (MD) scheme to dock ligands into a receptor binding site (Wu et al., 2003). The binding mode (Figure 4A) of *Sf* Top1-DNA-camptothecin indicated that the skeleton of camptothecin is likely to interact with the *Sf* Top1-DNA complex by a combination of π-π stacked interactions with the upstream and downstream base pairs (A965, T940, C964, and G941) in the nucleic acid. The two fundamental hydrogen bond interactions between the 20- hydroxyl and the 1-nitrogen atom with the Arg530 and Asp698 residue, respectively, are also shown in the figure. This combination mode has a strong resemblance to the binding mechanism of human topoisomerase 1 for TPT, and π-π stacked and hydrogen-bond interactions provide a significant contribution to the stability of the ternary complex (Staker et al., 2002). The pharmacophore around the 7-site of camptothecin (Figure 4B) displayed a big cavity and showed several chemical features such as hydrogen bond acceptor (HBA) and hydrogen bond donor (HBD), which are closest to position 7 of camptothecin and have two bases (A965 and G941) that can be defined as HBA and HBD. This results implied that a group containing both HBA and HBD has the potential to interact with the bases via hydrogen-bond interactions. Therefore, by considering the synthesis feasibility, the acylamino, an assumed group which is plausibly a perfect match as shown in Figure 4C, was designed on position 7. To test the idea computationally, the two simplest compounds (**4a** and **4b**, mentioned in Figure 4C), as well as the control camptothecin, were initially designed, prepared and docked into the *Sf* Top1-DNA binding site. The docking results (Figure 4D) showed that compound **4b** and camptothecin bound similarly in the cavity with their rigid rings inserting into the cleaved gap and forming H-bonds interactions with Asp698 and Arg530. Intriguingly, the rigid rings of compound **4a** showed a slight shift compared with compound **4b** and camptothecin, thus resulting in the 1-nitrogen atom instead of forming an H-bond interaction with the residue Arg530. It is noteworthy that the amide group on the compound **4b** has a srong H-bond interaction with the base G941, but it was not found in compound **4a**. Although the designed carbonyl group does not interact with the intended base, the free hydrogen atom on the amide of CPT may form a strong H-bond interaction with the nucleic acid bases and stabilize the ternary complex. These results implied that this type of 7-amide camptothecin could be used as a novel backbone structure, and other groups could be introduced onto the amide.

**Figure 5:**
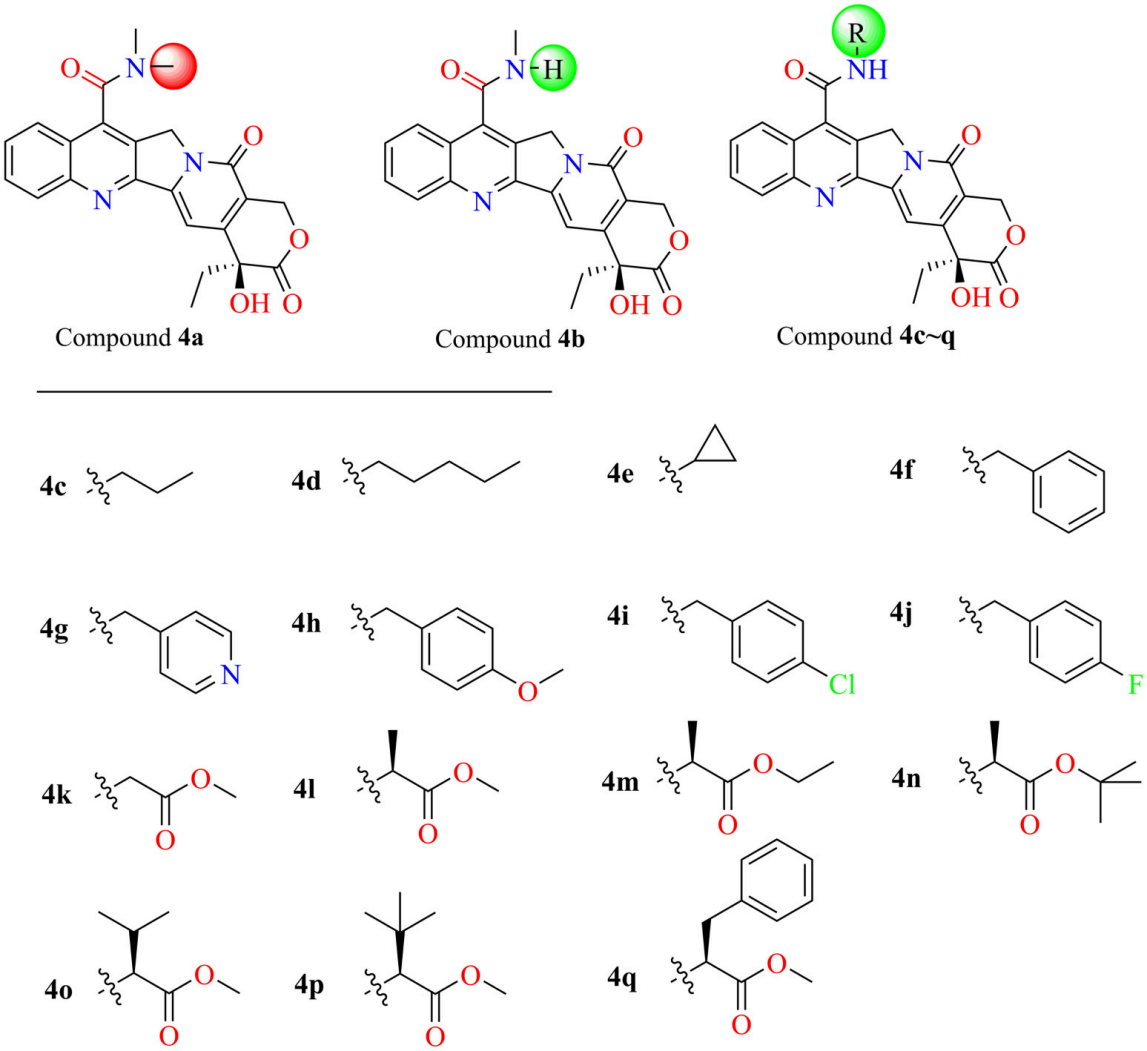
The structures of designed target compounds.

In the past five decades, a large number of camptothecin derivatives have been synthesized and applied in the anti-tumor field, and the structure-activity relationship (SAR) of CPTs was clear (Liu et al., 2015; Martino et al., 2017). Generally, substitutions at 7 with some liposolubility groups, such as alkyl, aryl, and alkynyl, could effectively improve the stability of CPTs (Li et al., 2009; Liu et al., 2011, 2012; Zhu et al., 2012). Hence, the synthesis strategy of CPTs was mainly to consider the stability and the combining capacity of compounds with the Top1-DNA complex. Therefore, by considering the size of the protein cavity, a series of compounds (**4a–f**) containing lipophilic groups such as alkyl and aromatic rings were subsequently designed. Moreover, to explore and exploit more substituents which could extend to the lumen of the *Sf* Top1-DNA complex and have a potential to stabilize the complex, the amine acid residues around 7-C of camptothecin in the cavity were subsequently analyzed. Two hydrophilic amino acids, Asn518 and Lys602 residues, which were closest to the position, and several hydrophobic residues (Met594 and Pro597) were exposed to the modification site of camptothecin (Figure 4B). Accordingly, a series of compounds (**4g–q**) listed in Figure 5 consisting of groups with electron-withdrawing or electron-donating capabilities were considered for potential combination with the *Sf* Top1-DNA complex.

### Chemistry

**Scheme 1:**
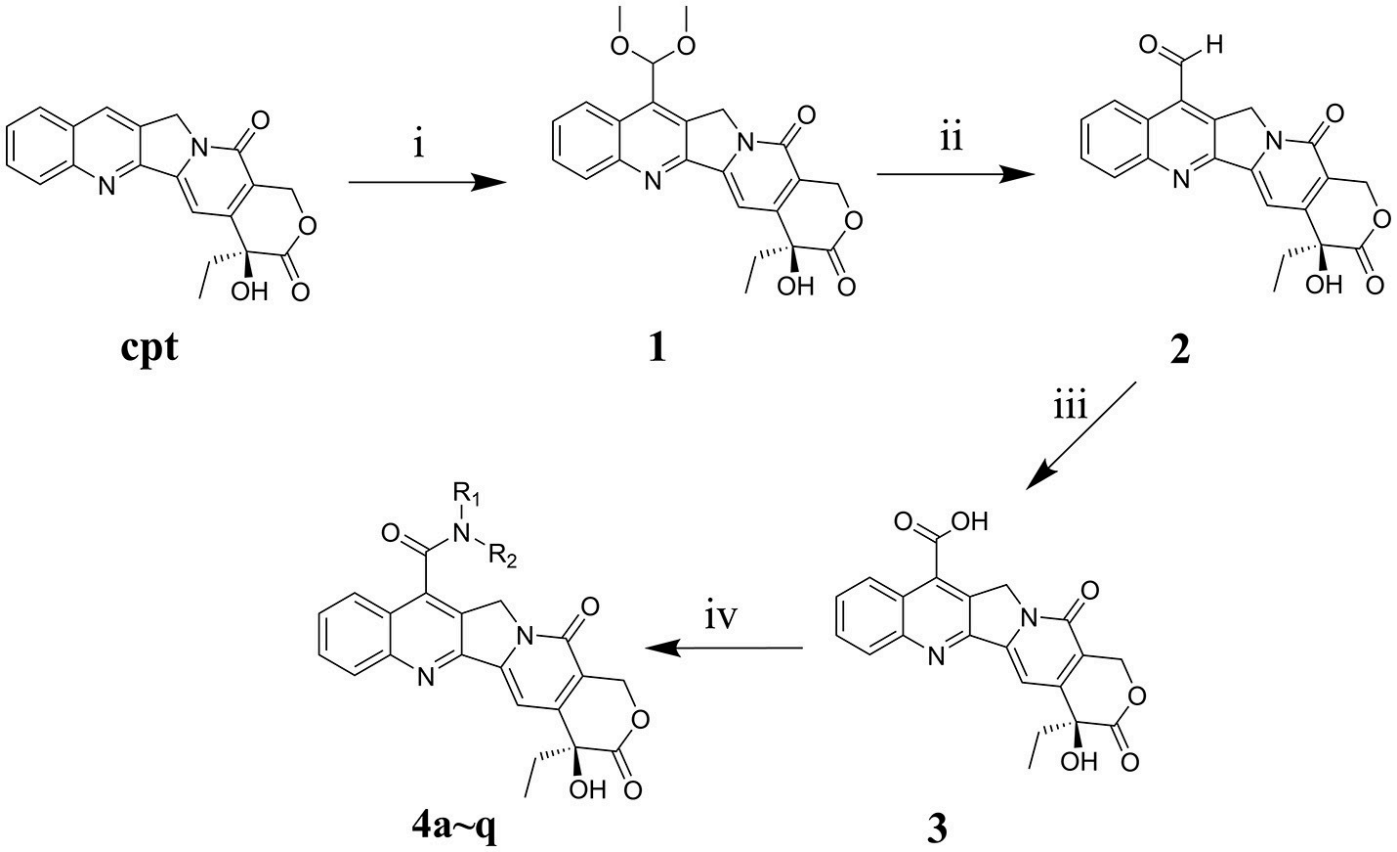
Synthesis routes of 7-amide camptothecin derivatives. Reagents and conditions: i, CH_3_OH, 96% H_2_SO_4_, ${\text{FeSO}}_{4}^{*}$10H_2_O, 30% H_2_O_2_, 3h, MnO_2_, 2h; ii, C_2_H_5_OH-H_2_O, 4h; iii, HCOOH, 30% H_2_O_2_, 40h, iv, Amide, EDCI, HOBt, DMF, 24 h.

The main strategy for synthesizing different amide compounds is the condensation reaction of carboxylic acids with various kinds of amines. Compound **3** was accumulated via an entire oxidizing system (Scheme 1) in three steps according to the material and methods section, and it has a reasonably impressive yield (42% in three steps). Compound **3** was dissolved in DMF, and EDCI and HOBt were then added to form a stable activated ester, while the target compounds were obtained by nucleophilic substitution of different amines with the activated ester in a decent yield between 39 and 60%.

### *In vitro* toxicity assay

The MTT assay was used to evaluate the cytotoxic activity of these CPTs against Sf9 cells for 24 h. The results shown in Table 1 indicated that camptothecin had a strong effect on Sf9 cells with an IC_50_ of 29.47 μM. As compared with camptothecin, the anti-proliferative activity of **3** (14.32 μM) had a specific extent increasing after the incorporation of a carboxyl group on the position 7 of CPT. Interestingly, compounds **4a** (IC_50_ > 50.00 μM) and **4b** (IC_50_ 6.26 μM) exhibited diametrically opposite cytotoxicity, and these experimental results also further validated the reasonability of the conjecture mentioned above. It is satisfying to note that all of the target compounds, except compound **4a** and **4q**, showed significant cytotoxic activities (the values of IC_50_ were between 2.01 and 6.78 μM) against the Sf9 cells. Amongst all the tested compounds, compound **4j** exhibited the most substantial anti-proliferative activity (2.01 μM), being 15-fold more potent than the positive control camptothecin. However, as illustrated by the results of the antiproliferative activity, compound **4q** showed less popular cytotoxic activity (IC_50_ > 50.00 μM) compared with that of other analogous. In an additional *in vitro* test, to confirm whether CPTs could specifically reduce the activity of *sf* Top1 as expected, pre-mixed purified protein and supercoiled DNA PBR322 were incubated with camptothecin and its derivatives, respectively. As shown in Supplementary Figure 1, the purified protein completely abolished the supercoiled form of PBR322 to the relaxation state, and CPT could significantly inhibit the relaxation activity of the enzyme. Most of the derivatives are also equivalent to the effects of CPT, meaning that CPTs are able to specifically influence the function of insect cell topoisomerase 1.

**Table 1:** Antiproliferative activity of CPTs against the Sf9 cell line.

**Compound**	**Toxicity regression equation**	**Correlation coefficient**	**IC_50_ (μM)**
Camptothecin	y = 0.4196 x + 4.5756	0.9502	29.47
3	y = 0.6486 x + 4.5138	0.9737	14.32
4a	y = 0.4890 x + 3.9788	/	>50.00
4b	y = 0.9302 x + 4.6235	0.9542	6.26
4c	y = 0.5307 x + 4.9295	0.9683	3.13
4d	y = 0.6845 x + 4.9595	0.9861	2.48
4e	y = 0.4074 x + 4.9289	0.9508	3.46
4f	y = 0.6280 x + 4.9054	0.9733	2.94
4g	y = 1.1000 x + 4.4340	0.9509	6.78
4h	y = 0.5943 x + 4.9180	0.9509	2.69
4i	y = 0.7029 x + 4.9022	0.9859	2.67
4j	y = 0.5036 x + 4.8634	0.9763	2.01
4k	y = 0.7624 x + 4.8663	0.9933	3.23
4l	y = 0.8915 x + 4.7481	0.9569	4.01
4m	y = 0.7298 x + 4.8800	0.9679	2.97
4n	y = 0.7321 x + 4.7543	0.9645	4.17
4o	y = 0.7812 x + 4.6980	0.9735	4.82
4p	y = 0.5373 x + 4.8328	0.9522	3.94
4q	y = 0.9867 x + 3.1877	/	>50.00

Among these compounds, some of the lipophilic alkyl groups, methyl (**4b**), propyl (**4c**), cyclopropyl (**4e**), and pentyl (**4d**), were respectively introduced onto the amide linkage, and these corresponding compounds not only presented considerable cytotoxicity, but also enhanced with the increasing length of the alkyl chain. In addition, compound **4f**, which links the lipophilic aromatic ring, also exhibited appreciable cytotoxicity. These results, similar to the structure-activity relationship in terms of the anti-tumor field, revealed that introduction of lipophilic groups might be beneficial to its activities (Liu et al., 2015). Furthermore, the compound (**4j**) that was connected with the electronegative halogen atom fluorine also displayed excellent antiproliferative activity as compared with compounds (**4g–i**) possessing similar substituents. Moreover, the compounds which contained different types of amino acid ester groups, except for **4q** that bore a large volume group in the side chain of the substituent, showed significant antiproliferative activity as well. These compounds with some small ester groups have the potential to combine with the protein and promote the activity, but a bulky group, generally would be hinder the formation of ternary complexes (Liu et al., 2015) even decrease the activity.

### Molecular docking study

**Figure 6:**
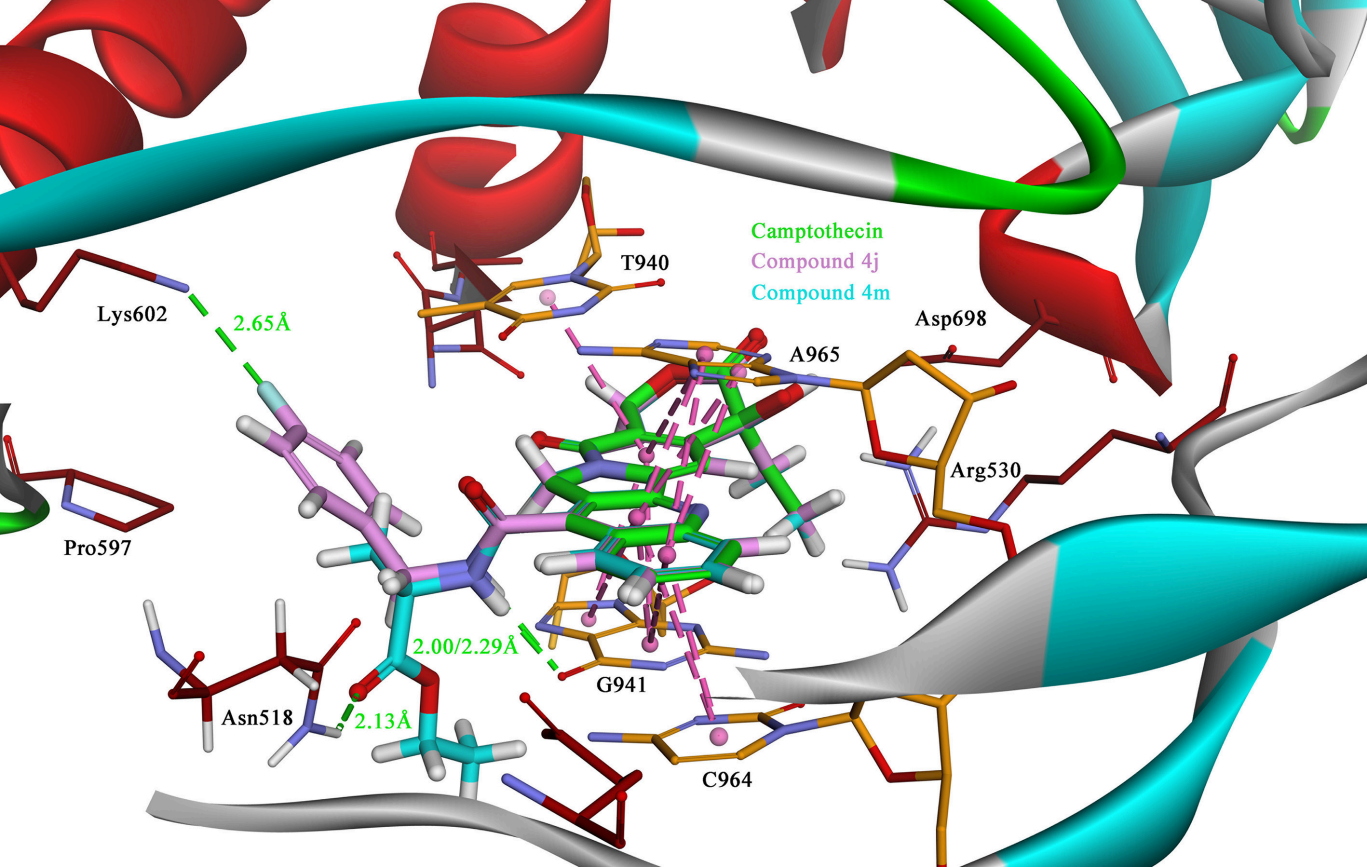
The binding modes of the *Sf* Top1-DNA in complex with three compounds, **4j** (lavender), **4m** (cyan) and camptothecin (green). The bases of DNA and the residues of protein are shown in orange and brown carbons, respectively. The H-bonds formed between the ligand and residues are indicated as green dashed lines, and the π-π stacked interactions are shown in lavender dashed lines.

Due to the target compounds exhibiting excellent activity against Sf9 cells, and aiming to explore the interaction between the derivatives with the protein-DNA complex, two compounds (**4j** and **4m**) consisted of two different groups with excellent activity, were selected for molecular docking studies. The binding modes of compounds **4j**, **4m**, and camptothecin are shown in Figure 6. The rigid rings of the three compounds were inserted into the four bases (T940, G941, C964, and A965) of DNA in parallel, and overlaid well with each other. Similar to the mode of camptothecin, the backbone of the derivatives could also form ternary complexes via π-π stacking, hydrophobic and hydrogen bonding interactions with DNA-protein complex. Moreover, the amide on the compounds **4j** and **4m** presented a strong hydrogen bond interaction with the base G941, and these interactions presented a respectively short distance of 2.29 and 2.00 Å. The difference in the binding modes of compounds **4j** and **4m** is mainly caused by the different groups attached to the amide, which could form non-covalent interactions with different amino acid residues of the protein. The 4-fluorobenzyl group on the **4j** might be extended to the cavity of the protein and could form a strong H-bond interaction with the residue Lys602. However, the type of ester group on the **4m** forms a strong H-bond with the other residue Asn518. Despite the discrepancy, the two compounds bound to the DNA-protein complex presented a high affinity with the binding free energy of −61.78 kcal/mol (**4j**) and −76.50 kcal/mol (**4m**) as compared to that of camptothecin (−31.43 kcal/mol), which means that these groups have contributed to the stability of the complex.

### Molecular dynamics simulation

To better observation that the contribution of compound **4j** to the stability of the complex, we have carried out a 50 ns molecular dynamical simulation of the system. The RMSD value, as shown in Supplementary Figure 2, for residues indicated that the system tends to be stable after 10 ns. Analysis of the per-residue root mean square fluctuation, RMSF, indicated that linker domain (residue: 801–876) being the most fluctuating region in the protein in Supplementary Figure 3. In contrast, the fluctuations in amino acid residues associated with the ligand binding were not severe, meaning that the protein system has greater stability upon binding to the compound. During the dynamical equilibrium process in Supplementary Figure 4, compound **4j** was always in the middle of the binding pocket without major shifts, and the six-membered ring structure remained strongly interacting with the amino acid residues, which is consistent with the docking results. In addition, it can be noted that the designed amide groups in this process often formed hydrogen bonds with the upper or lower bases (A965 and G941, respectively), which contributes to the stable ternary complex. However, the side chain group of **4j** fluctuated greatly during the simulation, but formed weak interactions with different amino acids, which may also play a role in the stability of the complex.

### Bioassay

**Figure 7:**
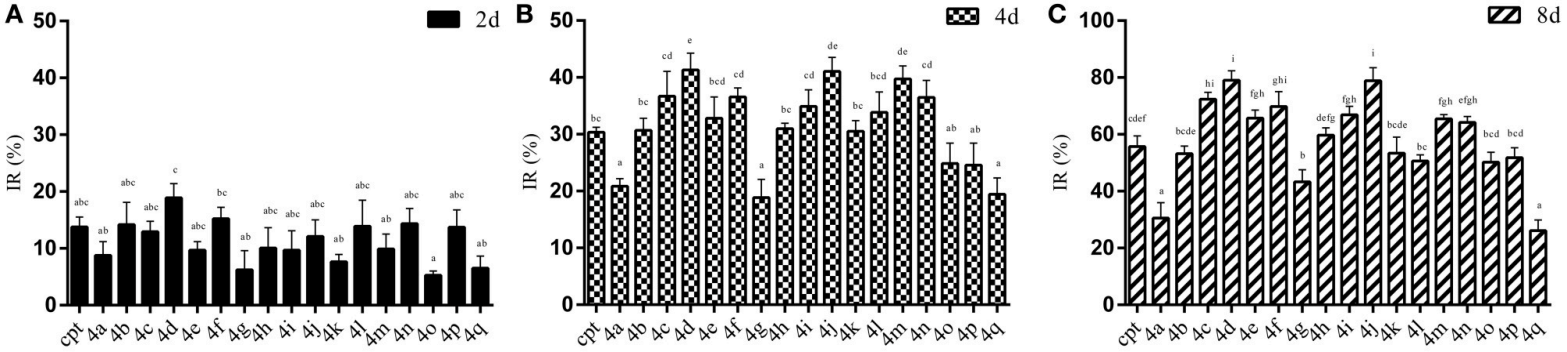
Effects of CPTs on the inhibiting rate of weight increase (IR) against *S. litura* 2rd larvae after 2 **(A)**, 4 **(B)**, and 8 **(C)** days of exposure, respectively. All data are represented by the mean ± SEM of three separate experiments. SPSS 22.0 software was used to perform the statistical analysis, and Duncan's test was performed with *P* = 0.05 representing significance.

The results of the IR of the larvae weight increase are shown in Figure 7. It can be seen that the increasing inhibition is in a time-dependent manner. All of the treatments presented less pronounced inhibitory effects (IR: 5.29~18.91%) on the 2rd *S. litura* larvae after 2 days of exposure (Figure 7A). However, with continuous exposure, the IR was significantly enhanced after treatment for 4 and 8 days (Figures 7B,**C**). Most of the tested compounds exhibited a good inhibitory activity (IR from 24.57 to 41.33%), superior or comparable to that of camptothecin (IR 30.35%) after 4 days. Some of them, **4d**, **4j**, **4m**, even showed a significantly higher activity than CPT (Figure 7B, *P* < 0.05). Furthermore, compounds **4a** and **4q**, presenting a similar insignificant inhibitory activity (IR 20.80 and 19.42%, respectively) in larvae, showed unsatisfactory cytotoxic activities in Sf9 cells. As the results illustrated in Figure 7C, almost all of the tested compounds could strongly inhibit the growth of insect larvae (IR from 50.20 to 79.05%) except compounds **4a**, **4g**, and **4q** (IR 30.54%, 43.27%, and 26.09%, respectively) after 8 days of exposure. In particular, four compounds (**4c**, **4d**, **4f**, and **4j**) exhibited a sturdy inhibition activity (more than 70%) that was significantly higher than camptothecin (IR 55.69%).

## Discussion

In the progress of drug design and high-throughput screening, some molecules, which belongs to Pan-Assay Interference Compounds (PAINS), disguised to bind with the target by other mechanisms and transmitted to other interferential signals for us, thus wasting a lot of resources to study on the PAINS (Baell and Holloway, 2010; Baell and Nissink, 2017). A modified compound, due to the addition or subtraction of a group, a large change in the skeletal structure may cause it to be a candidate for PAINS. Although camptothecin has been shown to be a specific active substance for topoisomerase 1 in human and some insect cells, but its derivative still have the possibility of being defined as PAINS for effects on the insect. In our work, we demonstrated that these derivatives significantly inhibited the activity of the *sf* Top1 protein as much as camptothecin *in vitro*, indicating the specific activity of the synthesized compounds on the insect. In fact, from the results of molecular dynamics simulations, we revealed the mechanism by which CPT derivatives act primarily by maintaining the stability of the complex through its six-membered ring, whereas the side chain groups can interact with protein/nucleic acids and further appropriately enhanced the stability. These evidences indicated that the mechanism of the modified camptothecin derivatives has not be changed in spite of the significantly structural changes, and ruled out the possibility of compounds becoming as PAINS for the insect.

To elucidate the specific mechanism of camptothecin derivatives, we performed molecular docking and molecular dynamics simulations. In the binding model of camptothecin with *Hs*Top 1, the rigid backbone structure of camptothecin forms a strong hydrophobic interaction with the base. In addition, the hydrogen bond interaction between Top1 and camptothecin provides an essential contribution to stabilizing the top-DNA complex, which is crucial for maintaining the activity (Staker et al., 2002). Among the camptothecin derivatives we designed, some compounds, such as **4j** and **4m** could form strong hydrogen bonds with the insect Top1. Interestingly, the amino acid residues Asn352 and Lys436 in *Hs*Top1, which are equivalent to Asn518 and Lys602 residues in *sf* Top1, may also form H-bond interactions with camptothecin derivatives to stabilize the ternary complex (Song et al., 2016). In different organisms, these two amino acid residues behave very conservatively, and they are located in the active pocket of the protein. In our study, the docking studies revealed the direct interactions between the residues Asn518 and Lys602 in the insect protein with some camptothecin derivatives we designed, and also illustrated these two residues may play critical roles in the binding of the ligand. However, in the results of the molecular dynamics simulation, the side chain of the derivative has a large volatility in the binding lumen, which is different from the docking results. The protein is inflexible during molecular docking study, and the initial structure of the protein has a large impact on the final binding model. CDOCKER molecular docking program based on the CHARMm force field can quickly predict the possible binding model of protein-ligand, but the inflexibility of protein and nucleic acid may compromise the accuracy of the results. In addition, the original CHARMm force field is not optimal for DNA during molecular docking simulation although it is particularly useful when mimicking proteins (Hart et al., 2012). In contrast, molecular dynamics simulation is better to predict the binding modes without the deficiency presenting in the docking simulations, and to verify whether proposed ligand remain in interactions with the listed residues. In fact, the binding model obtained by molecular dynamics simulation is similar to the molecular docking result, in which the compound interacts with the listed residues (Arg530, Asp698, and Lys697) and the bases via hydrogen bonding and combines with the nucleic acid by π-π stacking interactions. Overall, molecular docking simulation is still a good method to quickly predict the mainly protein-ligand binding, albeit with some deficiencies. However, molecular dynamics simulations can better explain the binding mechanism of protein-ligands in which amino acid residues in the binding lumen and ligand groups with greater flexibility. The revealed interaction mechanism by these simulations might be useful for further structural modifications and could provide valuable direction for exploring new potent compounds.

Although most of the designed compounds exhibited more pronounced activity (about 10-fold) *in vitro* than camptothecin, the activity *in vivo* did not wholly follow the law *in vitro*, and most of the compounds showed only equivalence to camptothecin. In general, camptothecin is able to enter the cells easily and interact with Top1, and then cause apoptosis and hinder cell growth (Zhong et al., 2016). Different from the traditional neurotoxic insecticides, CPTs generally exhibit a delayed insecticidal activity (Liu et al., 2010). In addition to inducing apoptosis in insect cells, camptothecin can also induce the midgut epithelial cells apoptosis in *S. litura* larva (Gong et al., 2014). However, insects have a complete detoxification system, including many detoxification enzymes, therefore, they may decompose the exogenous compounds and reduce their toxicity, which might lead to the different toxicity results obtained *in vitro* and *in vivo*, but the detailed mechanism that the relationship between activity *in vitro* and effectiveness *in vivo* remains unclear and needs to be explored further. Nevertheless, in our study, some compounds, such as **4c**, **4d**, **4f**, and **4j**, still show more significant activities than camptothecin both *in vitro* and *in vivo*, and are still hopeful to continue further research as the potential pesticides. In view of this, considering the safety of using these compounds on agricultural products that would be consumed by humans, the toxicological assessments of camptothecin derivatives for *environment* and organisms might be performed in the future. In the early report, 0.2% camptothecin emulsifiable concentrate (EC) showed strong toxicity for control of agricultural pests and no acute oral toxicity to the mouse (LD_50_ > 5000 mg/kg) nor acute dermal toxicity (LD_50_ > 2000 mg/kg) (Ma et al., 2010), which means that the camptothecin pesticide might have a safe dosage to mammals. A high dosage of oral or injectable camptothecin drugs easily caused side effects to humans in treatment of solid tumors. In general, the final residual pesticides degraded by microorganisms in the agricultural field are often safety to mammals. However, the oral acute and sub-acute toxicity of camptothecin derivatives remains to be further evaluated, and it is also necessary to explore the highest safety dosage of the drugs for humans (Manikandan and Kannan, 2015). In addition, the residue of camptothecin derivatives need to be tested by the field experiment in order to monitor the degradation dynamics of this kind of pesticide. After a period of safety interval, the residue of camptothecin pesticide could reach to a lower level which is safe for human beings. Moreover, considering the toxicity of this kind of pesticide for non-target organisms, especially whether they could cause lethal effect or infertility on mammals when applying in the field, more assessments should be taken into account. At the same time, these potential side effects could be alleviated by continually optimizing the structure. Overall, such compounds have the special mechanism on the insect and might have a good prospect for further development.

## Conclusions

The coding sequence of *Sf* Top1 was obtained, and the three-dimensional structure model was built according to the known information. The binding mode of camptothecin with *Sf* Top1-DNA complex was proposed. Besides, a total of seventeen novel 7-amide camptothecin derivatives were designed based on the mode of the *Sf* Top1-DNA-camptothecin complex via a rational analysis, and systematically synthesized. Almost all of the synthesized compounds were found to be more antiproliferative as compared with CPT. The molecular simulations revealed that the amide linkage of derivatives is likely to be formed by H-bond interaction with the DNA bases, and the introduction of some electronegative groups also has the potential to stabilize the ternary complexes via beneficial non-covalent interactions. Furthermore, the designed compounds, especially **4c**, **4d**, **4f**, and **4j**, which could strongly inhibit the growth of the *S. litura* larva are expected to be developed as potential biorational pesticides. All these results demonstrated the interaction mechanism between camptothecin and its derivatives with *Sf* Top1, and presented a structural model of *Sf* Top1-DNA that is useful for further structural modification and exploration of potential compounds.

## Author contributions

GZ, YL, and GS designed and supervised the study. ZJ and ZZ conducted the chemical synthesis under the supervision of GZ and GS. ZJ, GC, and ZS performed the biological experiments under the supervision of GZ. ZJ performed the computational simulations. All authors were involved in the data analysis and manuscript writing and approved the final manuscript.

### Conflict of interest statement

The authors declare that the research was conducted in the absence of any commercial or financial relationships that could be construed as a potential conflict of interest.
