# Supplementary material for: DNA Topoisomerase 1 Structure-BASED Design, Synthesis, Activity Evaluation and Molecular Simulations Study of New 7-Amide Camptothecin Derivatives Against Spodoptera frugiperda
Source: Front Chem. 2018 Oct 5;6:456. doi: 10.3389/fchem.2018.00456 (PMC6182061; doi:10.3389/fchem.2018.00456)
Supplement: Supplementary file 1 [file Data_Sheet_1.docx]

***Supplementary Material***

**DNA topoisomerase 1 structure-based design, synthesis, activity evaluation and molecular simulations study of new 7-amide** **camptothecin derivatives against *Spodoptera frugiperda***

**Zhiyan Jiang^1^, Zhijun Zhang^1^, Gaofeng Cui^1^, Zhipeng Sun^1^, Gaopeng Song^2^, Yingqian Liu^3*^, Guohua Zhong^1*^**

^1^Key Laboratory of Natural Pesticide and Chemical Biology, Ministry of Education, and Key Laboratory of Crop Integrated Pest Management in South China, Ministry of Agriculture, College of Agriculture, South China Agricultural University*,* Guangzhou*,* China.

^2^College of Materials and Energy, South China Agricultural University, Guangzhou, China.

^3^School of Pharmacy, Lanzhou University, Lanzhou, China.

*** Correspondence:**Yingqian Liu
yqliu@lzu.edu.cn

Guohua Zhong
guohuazhong@scau.edu.cn

**1.** **The specific inhibit activity of CPTs for *Sf*Top1
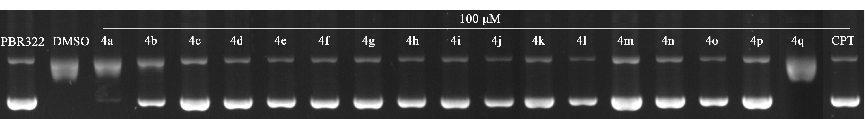
**

**Supplementary Figure 1.** The specific inhibit activity of CPTs for *Sf*Top1 *in vitro*. A quantity of 20 ng of puriﬁed protein was incubated with a concentration of 100 μM of various CPTs. pBR322 and DMSO denote no protein inside and no CPTs, respectively.

**2. The supplementary figures of molecular dynamical simulations**

**
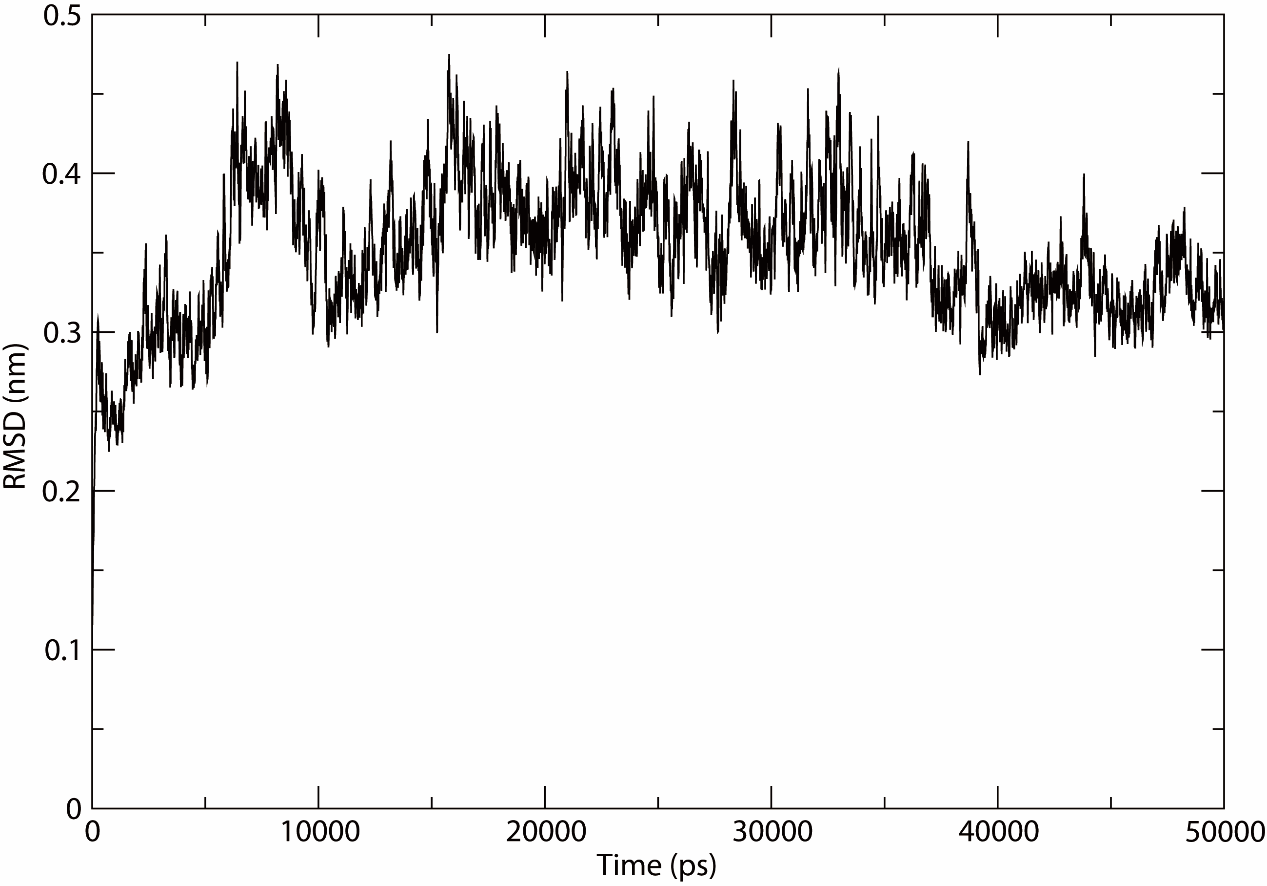
**

**Supplementary Figure 2.** Illustration of the root-mean-square displacement (RMSD)

**
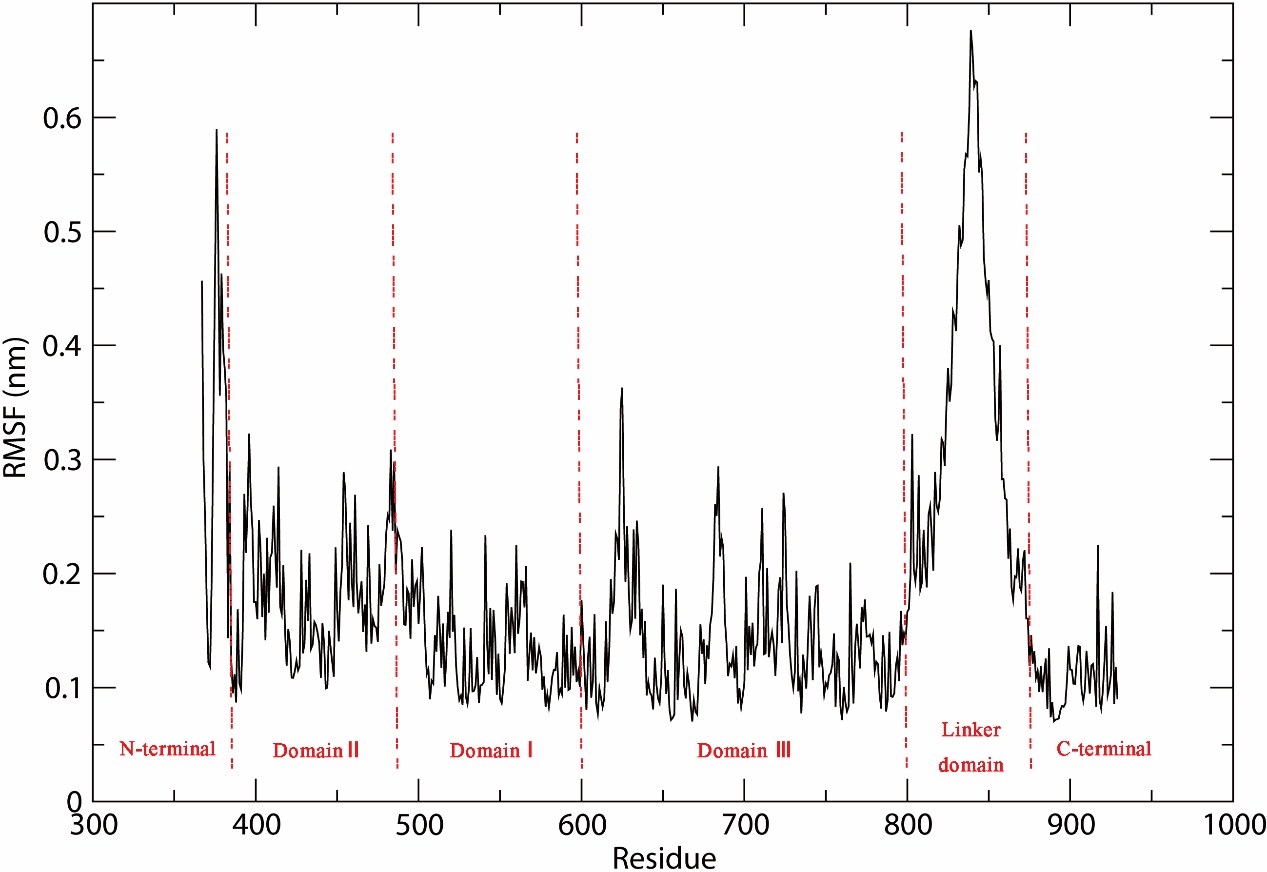
**

**Supplementary Figure 3.** Per-residue Root Mean Square Fluctuation (RMSF) of the enzyme *sf*Top1

**
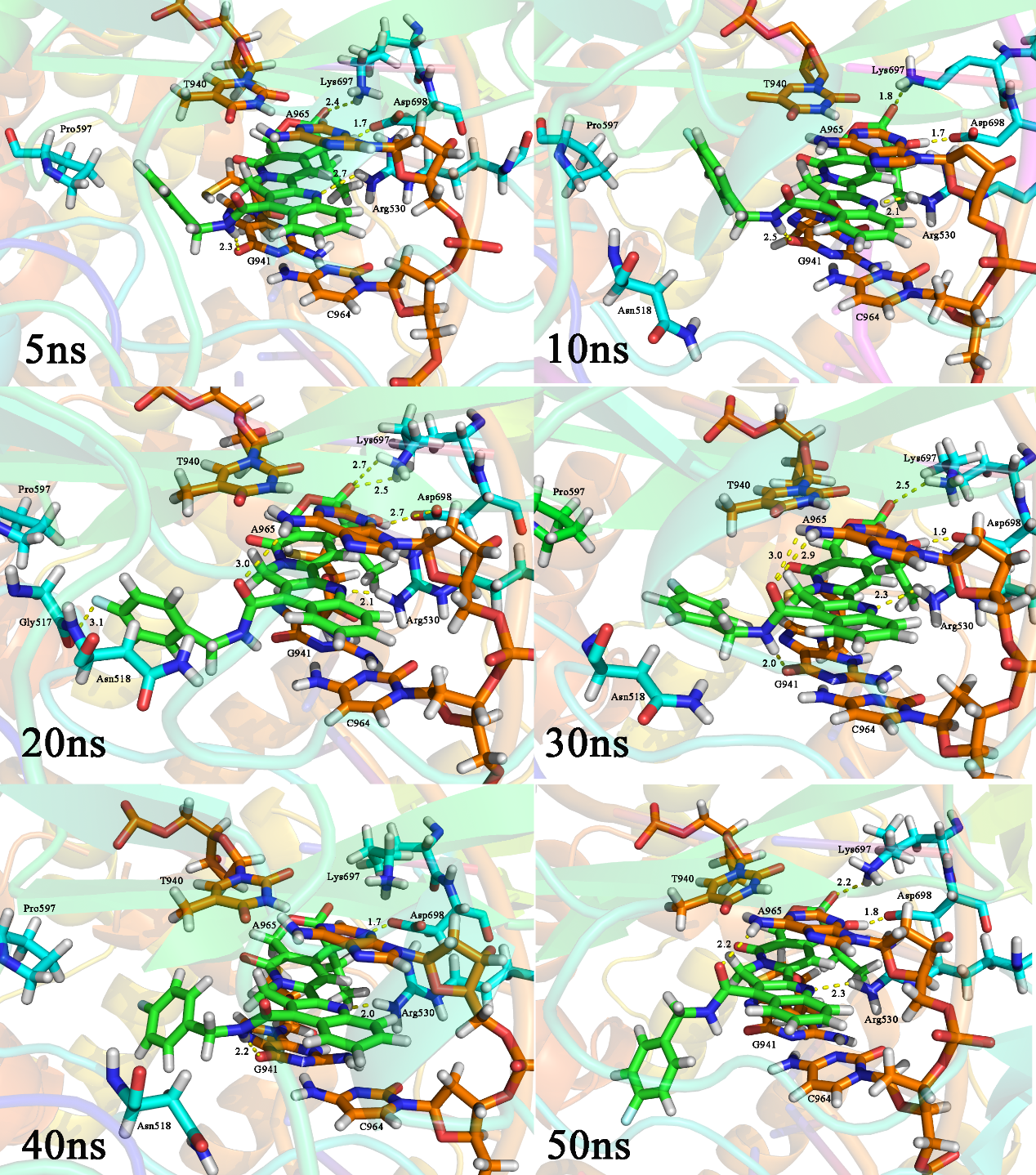
**

**Supplementary Figure 4. The** Combined conformational changes at 5, 10, 20, 30, 40 and 50 ns in molecular dynamics simulation.

**3. ^1^H NMR, ^13^C NMR and HRMS spectra data for target compounds**

***(S)-4-ethyl-4-hydroxy-N,N-dimethyl-3,14-dioxo-3,4,12,14-tetrahydro-1H-pyrano[3',4': 6,7]indolizino[1,2-b]quinoline-11-carboxamide (4a)***

Compound **4a** was synthesized as a yellow solid in 38% yield; ^1^H NMR (600 MHz, DMSO-*d*_6_) *δ* 8.25 (d, *J* = 8.4 Hz, 1H, Ar-H), 7.93 (t, *J* = 7.6 Hz, 1H, Ar-H), 7.85 (d, *J* = 8.3 Hz, 1H, Ar-H), 7.77 (t, *J* = 7.6 Hz, 1H, Ar-H), 7.37 (s, 1H, Ar-H), 6.56 (s, 1H, OH), 5.44 (s, 2H, CH_2_), 5.22 (d, *J* = 18.5 Hz, 1H, CH-H), 5.09 (d, *J* = 18.5 Hz, 1H, CH-H), 3.20 (s, 3H, CH_3_), 2.79 (s, 3H, CH_3_), 1.87 (m, 2H, CH_2_CH_3_), 0.88 (t, *J* = 7.3 Hz, 3H, CH_2_CH_3_); ^13^C NMR (151 MHz, DMSO-*d*_6_) *δ* 172.85, 165.57, 157.20, 153.38, 150.49, 148.62, 145.76, 138.40, 131.26, 130.18, 129.12, 126.40, 125.55, 124.05, 119.91, 97.45, 72.82, 65.72, 49.77, 37.85, 34.62, 30.83, 8.20. HRMS (ESI): calcd for C_23_H_21_N_3_O_5_ (M-H^+^) 418.1481, found 418.1405.

***(S)-4-ethyl-4-hydroxy-N-methyl-3,14-dioxo-3,4,12,14-tetrahydro-1H-pyrano[3',4': 6,7]indolizino[1,2-b]quinoline-11-carboxamide (4b)***

Compound **4b** was synthesized as a yellow solid in 42% yield; ^1^H NMR (600 MHz, DMSO-*d*_6_) *δ* 8.84 (d, *J* = 4.6 Hz, 1H, NH), 8.23 (d, *J* = 8.5 Hz, 1H, Ar-H), 8.14 (d, *J* = 8.4 Hz, 1H, Ar-H), 7.92 (t, *J* = 7.6 Hz, 1H, Ar-H), 7.80 – 7.74 (m, 1H, Ar-H), 7.37 (s, 1H, Ar-H), 6.55 (s, 1H, OH), 5.44 (s, 2H, CH_2_), 5.30 (s, 2H, CH_2_), 2.97 (d, *J* = 4.6 Hz, 3H, NHCH_3_), 1.87 (m, 2H, CH_2_CH_3_), 0.88 (t, *J* = 7.3 Hz, 3H, CH_2_CH_3_); ^13^C NMR (151 MHz, DMSO*-d_6_*) *δ* 172.86, 165.21, 157.17, 153.00, 150.55, 148.96, 145.48, 138.48, 131.17, 129.98, 128.86, 126.93, 126.26, 124.70, 120.01, 97.43, 72.84, 65.70, 50.29, 30.74, 26.58, 8.25; HRMS (ESI): calcd for C_22_H_19_N_3_O_5_ (M+H^+^) 406.1325, found 406.1397.

***(S)-4-ethyl-4-hydroxy-3,14-dioxo-N-propyl-3,4,12,14-tetrahydro-1H-pyrano[3',4': 6,7]indolizino[1,2-b]quinoline-11-carboxamide (4c)***

Compound **4c** was synthesized as a yellow solid in 45% yield; ^1^H NMR (600 MHz, DMSO-*d*_6_) *δ* 8.91 (t, *J* = 5.7 Hz, 1H, NH), 8.24 (d, *J* = 8.4 Hz, 1H, Ar-H), 8.12 (d, *J* = 8.4 Hz, 1H, Ar-H), 7.93 (t, *J* = 7.7 Hz, 1H, Ar-H), 7.78 (t, *J* = 7.6 Hz, 1H, Ar-H), 7.37 (s, 1H, Ar-H), 6.56 (s, 1H, OH), 5.44 (s, 2H, CH_2_), 5.28 (s, 2H, CH_2_), 3.41 (m, 2H, NHCH_2_CH_2_CH_3_), 1.88 (m, 2H, CH_2_CH_3_), 1.65 (m, 2H, NHCH_2_CH_2_CH_3_), 1.00 (t, *J* = 7.4 Hz, 3H, NHCH_2_CH_2_CH_3_), 0.89 (t, *J* = 7.3 Hz, 3H, CH_2_CH_3_); ^13^C NMR (151 MHz, DMSO*-d_6_*) *δ* 177.51, 169.49, 161.94, 157.75, 155.30, 153.75, 150.27, 143.51, 135.86, 134.75, 133.59, 131.46, 130.82, 129.48, 124.77, 102.18, 77.59, 70.48, 54.87, 46.19, 35.68, 27.55, 16.71, 12.95; HRMS (ESI): calcd for C_24_H_23_N_3_O_5_ (M+H^+^) 434.1638, found 434.1712.

***(S)-4-ethyl-4-hydroxy-3,14-dioxo-N-pentyl-3,4,12,14-tetrahydro-1H-pyrano[3',4': 6,7]indolizino[1,2-b]quinoline-11-carboxamide (4d)***

Compound **4d** was synthesized as a yellow solid in 43% yield; ^1^H NMR (600 MHz, DMSO-*d*_6_) *δ* 8.91 (t, *J* = 5.6 Hz, 1H, NH), 8.24 (d, *J* = 8.5 Hz, 1H, Ar-H), 8.11 (d, *J* = 8.4 Hz, 1H, Ar-H), 7.92 (t, *J* = 7.6 Hz, 1H, Ar-H), 7.78 (t, *J* = 7.6 Hz, 1H, Ar-H), 7.37 (s, 1H, Ar-H), 6.56 (s, 1H, OH), 5.44 (s, 2H, CH_2_), 5.28 (s, 2H, CH_2_), 3.44 (m, 2H, NHCH_2_C_4_H_9_), 1.88 (m, 2H, CH_2_CH_3_), 1.69 – 1.59 (m, 2H, NHCH_2_CH_2_C_3_H_7_), 1.44 – 1.34 (m, 4H, NHC_2_H_4_CH_2_CH_2_CH_3_), 0.94 (t, *J* = 7.0 Hz, 3H, NHC_4_H_8_CH_3_), 0.89 (t, *J* = 7.3 Hz, 3H, CH_2_CH_3_); ^13^C NMR (151 MHz, DMSO-*d*_6_) *δ* 172.87, 164.66, 157.16, 152.99, 150.54, 148.92, 145.47, 138.64, 131.17, 130.00, 128.89, 126.76, 126.06, 124.69, 120.02, 97.46, 72.84, 65.70, 50.17, 30.75, 29.17, 29.10, 22.29, 14.44, 8.24 ; HRMS (ESI): calcd for C_25_H_27_N_3_O_5_ (M+H^+^) 462.1951, found 462.2025.

***(S)-N-cyclopropyl-4-ethyl-4-hydroxy-3,14-dioxo-3,4,12,14-tetrahydro-1H-pyrano[3',4': 6,7]indolizino[1,2-b]quinoline-11-carboxamide (4e)***

Compound **4e** was synthesized as a yellow solid in 40% yield; ^1^H NMR (600 MHz, DMSO-*d*_6_) *δ* 8.99 (d, *J* = 4.1 Hz, 1H, NH), 8.23 (d, *J* = 8.4 Hz, 1H, Ar-H), 8.12 (d, *J* = 8.2 Hz, 1H, Ar-H), 7.92 (t, *J* = 7.6 Hz, 1H, Ar-H), 7.78 (t, *J* = 7.7 Hz, 1H, Ar-H), 7.37 (s, 1H, Ar-H), 6.56 (s, 1H, OH), 5.45 (s, 2H, CH_2_), 5.27 (s, 2H, CH_2_), 3.04 (m, 1H, NHCH), 1.88 (m, 2H, CH_2_CH_3_), 0.88 (t, *J* = 7.3 Hz, 3H, CH_2_CH_3_), 0.85 – 0.81 (m, 2H, CH_2_), 0.69 – 0.63 (m, 2H, CH_2_)；^13^C NMR (151 MHz, DMSO-*d*_6_) *δ* 172.86, 165.91, 157.16, 152.95, 150.53, 148.92, 145.42, 138.33, 131.20, 129.98, 128.95, 126.84, 126.10, 124.63, 120.03, 97.47, 72.84, 65.70, 50.07, 30.74, 23.31, 8.24, 6.46, 6.42；HRMS (ESI): calcd for C_24_H_21_N_3_O_5_ (M+H^+^) 432.1481, found 432.1552.

***(S)-N-benzyl-4-ethyl-4-hydroxy-3,14-dioxo-3,4,12,14-tetrahydro-1H-pyrano[3',4': 6,7]indolizino[1,2-b]quinoline-11-carboxamide (4f)***

Compound 4f was synthesized as a yellow solid in 50% yield; ^1^H NMR (600 MHz, DMSO-*d*_6_) *δ* 9.45 (t, *J* = 5.3 Hz, 1H, NH), 8.24 (d, *J* = 8.3 Hz, 1H, Ar-H), 8.13 (d, *J* = 8.3 Hz, 1H, Ar-H), 7.98 – 7.88 (m, 1H, Ar-H), 7.82 – 7.73 (m, 1H, Ar-H), 7.40 (m, 6H, Ar-H), 6.56 (s, 1H, OH), 5.44 (s, 2H, CH_2_), 5.29 (s, 2H, CH_2_), 4.67 (d, *J* = 5.5 Hz, 2H, NHCH_2_), 1.95 – 1.81 (m, 2H, CH_2_CH_3_), 0.89 (t, *J* = 7.0 Hz, 3H, CH_2_CH_3_); ^13^C NMR (151 MHz, DMSO-*d*_6_) *δ* 172.86, 164.90, 157.13, 153.02, 150.53, 148.93, 145.43, 139.19, 138.21, 131.18, 130.04, 129.02, 128.95 (two), 128.00 (two), 127.68, 126.96, 126.01, 124.68, 120.02, 97.47, 72.84, 65.69, 50.23, 43.30, 30.77, 8.24; HRMS (ESI): calcd for C_28_H_23_N_3_O_5_ (M-H^+^) 480.1638, found 480.1551.

***(S)-4-ethyl-4-hydroxy-3,14-dioxo-N- (pyridin-4-ylmethyl)-3,4,12,14-tetrahydro-1H-pyrano[3',4': 6,7]indolizino[1,2-b]quinoline-11-carboxamide (4g)***

Compound **4g** was synthesized as a yellow solid in 50% yield; ^1^H NMR (600 MHz, DMSO-*d*_6_) *δ* 9.54 (t, *J* = 6.0 Hz, 1H, NH), 8.60 (d, *J* = 1.6 Hz, 1H, Ar-H), 8.59 (d, *J* = 1.5 Hz, 1H, Ar-H), 8.26 (d, *J* = 8.4 Hz, 1H, Ar-H), 8.15 (d, *J* = 7.7 Hz, 1H, Ar-H), 7.94 (t, *J* = 7.7 Hz, 1H, Ar-H), 7.80 (t, *J* = 8.3 Hz, 1H, Ar-H), 7.45 (d, *J* = 1.4 Hz, 1H, Ar-H), 7.44 (d, *J* = 1.5 Hz, 1H, Ar-H), 7.38 (s, 1H, Ar-H), 6.56 (s, 1H, OH), 5.45 (s, 2H, CH_2_), 5.33 (s, 2H, CH_2_), 4.70 (d, *J* = 6.0 Hz, 2H, NHCH_2_), 1.95 – 1.82 (m, 2H, CH_2_CH_3_), 0.89 (t, *J* = 7.3 Hz, 3H, CH_2_CH_3_); 13C NMR (151 MHz, DMSO-*d*_6_) *δ* 172.84, 165.22, 157.13, 153.05, 150.52, 150.21 (two), 148.94, 148.16, 145.39, 137.84, 131.24, 130.07, 129.06, 127.15, 126.00, 124.60, 122.85 (two), 120.05, 97.47, 72.83, 65.69, 50.29, 42.38, 30.75, 8.24; HRMS (ESI): calcd for C_27_H_24_N_4_O_4_ (M-H^-^)481.1590, found 481.1536.

***(S)-4-ethyl-4-hydroxy-N- (4-methoxybenzyl)-3,14-dioxo-3,4,12,14-tetrahydro-1H-pyrano[3',4': 6,7]indolizino[1,2-b]quinoline-11-carboxamide (4h)***

Compound **4h** was synthesized as a yellow solid in 50% yield; ^1^H NMR (600 MHz, DMSO-*d*_6_) *δ* 9.38 (t, *J* = 5.9 Hz, 1H, NH), 8.24 (d, *J* = 8.5 Hz, 1H, Ar-H), 8.12 (d, *J* = 8.4 Hz, 1H, Ar-H), 7.92 (t, *J* = 7.6 Hz, 1H, Ar-H), 7.77 (t, *J* = 7.6 Hz, 1H, Ar-H), 7.38 (s, 1H, Ar-H), 7.38 – 7.35 (m, 2H, Ar-H), 6.97 (d, *J* = 2.0 Hz, 1H, Ar-H), 6.96 (d, *J* = 1.9 Hz, 1H, Ar-H), 6.56 (s, 1H, OH), 5.44 (s, 2H, CH_2_), 5.27 (s, 2H, CH_2_), 4.59 (d, *J* = 5.8 Hz, 2H, NHCH_2_), 3.77 (s, 3H, OCH_3_), 1.95 – 1.81 (m, 2H, CH_2_CH_3_), 0.88 (t, *J* = 7.3 Hz, 3H, CH_2_CH_3_); ^13^C NMR (151 MHz, DMSO-*d*_6_) *δ* 172.86, 164.75, 158.94, 157.14, 153.01, 150.53, 148.92, 145.44, 138.28, 131.18, 131.12, 130.03 (two), 129.40, 128.93, 126.91, 126.02, 124.69, 120.01, 114.39 (two), 97.46, 72.84, 65.69, 55.58, 50.21, 42.74, 30.76, 8.24; HRMS (ESI): calcd for C_29_H_25_N_3_O_6_ (M-H^+^) 510.1743, found 510.1662.

***(S)-N- (4-chlorobenzyl)-4-ethyl-4-hydroxy-3,14-dioxo-3,4,12,14-tetrahydro-1H-pyrano[3',4': 6,7]indolizino[1,2-b]quinoline-11-carboxamide (4i)***

Compound **4i** was synthesized as a yellow solid in 47% yield; ^1^H NMR (600 MHz, DMSO-*d*_6_) *δ* 9.48 (t, *J* = 5.9 Hz, 1H, NH), 8.24 (d, *J* = 8.5 Hz, 1H, Ar-H), 8.12 (d, *J* = 8.3 Hz, 1H, Ar-H), 7.93 (t, *J* = 7.6 Hz, 1H, Ar-H), 7.81 – 7.76 (m, 1H, Ar-H), 7.50 – 7.45 (m, 4H, Ar-H), 7.37 (s, 1H, Ar-H), 6.56 (s, 1H, OH), 5.44 (s, 2H, CH_2_), 5.28 (s, 2H, CH_2_), 4.65 (d, *J* = 5.9 Hz, 2H, NHCH_2_), 1.88 (m, 2H, CH_2_CH_3_), 0.88 (t, *J* = 7.3 Hz, 3H, CH_2_CH_3_); ^13^C NMR (151 MHz, DMSO-*d*_6_) *δ* 172.82, 164.94, 157.12, 153.02, 150.52, 148.93, 145.40, 138.28, 138.05, 132.22, 131.19, 130.04, 129.94 (two), 128.95 (two), 127.01, 125.98, 124.63, 120.03, 97.46, 72.83, 65.69, 50.22, 49.06, 42.65, 30.78, 8.23; HRMS (ESI): calcd for C_28_H_22_ClN_3_O_5_ (M-H^+^) 514.1248, found 514.1173.

***(S)-4-ethyl-N- (4-fluorobenzyl)-4-hydroxy-3,14-dioxo-3,4,12,14-tetrahydro-1H-pyrano[3',4': 6,7]indolizino[1,2-b]quinoline-11-carboxamide (4j)***

Compound **4j** was synthesized as a yellow solid in 60% yield; ^1^H NMR (600 MHz, DMSO-*d*_6_) *δ* 9.44 (t, *J* = 5.8 Hz, 1H, NH), 8.24 (d, *J* = 8.4 Hz, 1H, Ar-H), 8.11 (d, *J* = 8.3 Hz, 1H, Ar-H), 7.95 – 7.89 (m, 1H, Ar-H), 7.80 – 7.75 (m, 1H, Ar-H), 7.50 (d, *J* = 5.8 Hz, 1H, Ar-H), 7.49 (d, *J* = 5.7 Hz, 1H, Ar-H), 7.37 (s, 1H, Ar-H), 7.23 (m, 2H, Ar-H), 6.55 (s, 1H, OH), 5.44 (s, 2H, CH_2_), 5.27 (s, 2H, CH_2_), 4.65 (d, *J* = 5.8 Hz, 2H, NHCH_2_), 1.88 (m, 2H, CH_2_CH_3_), 0.88 (t, *J* = 7.2 Hz, 3H, CH_2_CH_3_); ^13^C NMR (151 MHz, DMSO-*d*_6_) *δ* 172.84, 164.88, 157.13, 153.02, 150.53, 148.94, 145.42, 138.13, 135.42, 131.19, 130.15 (two), 130.09, 128.97, 126.98, 125.98, 124.65, 120.03, 115.82 (two), 115.68, 97.46, 72.83, 65.69, 50.19, 42.61, 30.79, 8.23; HRMS (ESI): calcd for C_28_H_22_FN_3_O_5_ (M-H^+^) 498.1543, found 498.1471.

***(S)-methyl 2- (4-ethyl-4-hydroxy-3,14-dioxo-3,4,12,14-tetrahydro-1H-pyrano[3',4': 6,7]indolizino[1,2-b]quinoline-11-carboxamido)acetate (4k)***

Compound **4k** was synthesized as a yellow solid in 57% yield; ^1^H NMR (600 MHz, DMSO-*d*_6_) *δ* 9.42 (t, *J* = 5.9 Hz, 1H, NH), 8.25 – 8.20 (m, 2H, Ar-H), 7.94 (t, *J* = 7.7 Hz, 1H, Ar-H), 7.80 (t, *J* = 7.6 Hz, 1H, Ar-H), 7.38 (s, 1H, Ar-H), 6.56 (s, 1H, OH), 5.45 (s, 2H, CH_2_), 5.32 (s, 2H, CH_2_), 4.25 (d, *J* = 6.3 Hz, 2H, NHCH_2_), 3.78 (s, 3H, OCH_3_), 1.93 – 1.80 (m, 2H, CH_2_CH_3_), 0.89 (t, *J* = 7.3 Hz, 3H, CH_2_CH_3_); ^13^C NMR (151 MHz, DMSO-*d*_6_) δ 172.87, 170.47, 165.67, 157.19, 153.05, 150.53, 148.89, 145.44, 137.81, 131.27, 129.99, 128.96, 127.15, 126.12, 124.64, 120.06, 97.48, 72.84, 65.70, 52.55, 50.27, 41.47, 30.74, 8.25; HRMS (ESI): calcd for C_24_H_21_N_3_O_7_ (M+H^+^) 464.1380, found 464.1463.

***(S)-methyl 2- ( (S)-4-ethyl-4-hydroxy-3,14-dioxo-3,4,12,14-tetrahydro-1H-pyrano[3',4': 6,7]indolizino[1,2-b]quinoline-11-carboxamido)propanoate (4l)***

Compound **4l** was synthesized as a yellow solid in 55% yield; ^1^H NMR (600 MHz, DMSO-*d*_6_) *δ* 9.40 (d, *J* = 7.0 Hz, 1H, NH), 8.25 (d, *J* = 8.5 Hz, 1H, Ar-H), 8.18 (d, *J* = 8.4 Hz, 1H, Ar-H), 7.97 – 7.90 (m, 1H, Ar-H), 7.80 (t, *J* = 7.6 Hz, 1H, Ar-H), 7.38 (s, 1H, Ar-H), 6.56 (s, 1H, OH), 5.45 (s, 2H, CH_2_), 5.35 (d, *J* = 18.9 Hz, 1H, CH-H), 5.26 (d, *J* = 18.9 Hz, 1H, CH-H), 4.69 (p, *J* = 7.3 Hz, 1H, NHCH), 3.79 (s, 3H, OCH_3_), 1.95 – 1.81 (m, 2H, CH_2_CH_3_), 1.46 (d, *J* = 7.3 Hz, 3H, NHCHCH_3_), 0.89 (t, *J* = 7.3 Hz, 3H, CH_2_CH_3_); ^13^C NMR (151 MHz, DMSO-*d*_6_) *δ* 173.10, 172.83, 165.00, 157.17, 153.00, 150.51, 148.86, 145.40, 137.89, 131.25, 129.97, 128.95, 127.09, 126.06, 124.64, 120.06, 97.48, 72.84, 65.70, 52.71, 50.15, 48.66, 30.78, 17.07, 8.24; HRMS (ESI): calcd for C_25_H_23_N_3_O_7_ (M+H^+^) 478.1536, found 478.1619.

***(S)-ethyl 2- ( (S)-4-ethyl-4-hydroxy-3,14-dioxo-3,4,12,14-tetrahydro-1H-pyrano[3',4': 6,7]indolizino[1,2-b]quinoline-11-carboxamido)propanoate (4m)***

Compound **4m** was synthesized as a yellow solid in 53% yield; ^1^H NMR (600 MHz, CDCl_3_) *δ* 8.16 (d, *J* = 8.4 Hz, 1H, Ar-H), 8.09 (d, *J* = 8.3 Hz, 1H, Ar-H), 7.78 (t, *J* = 7.6 Hz, 1H, Ar-H), 7.65 (d, *J* = 7.3 Hz, 1H, NH), 7.59 (t, *J* = 7.6 Hz, 1H, Ar-H), 7.54 (s, 1H, Ar-H), 5.42 (d, *J* = 16.2 Hz, 1H, CH-H), 5.31 (s, 1H, OH), 5.20 (d, *J* = 19.4 Hz, 1H, CH-H), 5.14 (d, *J* = 16.2 Hz, 1H, CH-H), 5.07 (d, *J* = 19.4 Hz, 1H, CH-H), 4.97 (p, *J* = 7.3 Hz, 1H, NHCH), 4.37 (q, *J* = 7.1 Hz, 2H, OCH_2_CH_3_), 1.90 – 1.82 (m, 2H, CH_2_CH_3_), 1.76 (d, *J* = 7.3 Hz, 3H, NHCHCH_3_), 1.41 (t, *J* = 7.1 Hz, 3H, OCH_2_CH_3_), 1.02 (t, *J* = 7.3 Hz, 3H, CH_2_CH_3_); ^13^C NMR (151 MHz, CDCl_3_) *δ* 173.46, 172.38, 164.69, 157.16, 151.96, 150.09, 149.09, 145.36, 137.17, 131.07, 130.05, 128.88, 125.88, 125.01, 123.86, 119.10, 98.37, 72.63, 66.04, 62.11, 49.74, 49.03, 31.62, 18.15, 14.24, 7.81; HRMS (ESI): calcd for C_26_H_25_N_3_O_7_ (M+H^+^) 492.1693, found 492.1760.

***(S)-tert-butyl 2- ( (S)-4-ethyl-4-hydroxy-3,14-dioxo-3,4,12,14-tetrahydro-1H-pyrano[3',4': 6,7]indolizino[1,2-b]quinoline-11-carboxamido)propanoate (4n)***

Compound **4n** was synthesized as a yellow solid in 50% yield; ^1^H NMR (600 MHz, DMSO-*d*_6_) *δ* 9.32 (d, *J* = 6.8 Hz, 1H, NH), 8.25 (d, *J* = 8.5 Hz, 1H, Ar-H), 8.21 (d, *J* = 8.4 Hz, 1H, Ar-H), 7.93 (t, *J* = 7.7 Hz, 1H, Ar-H), 7.79 (t, *J* = 7.6 Hz, 1H, Ar-H), 7.38 (s, 1H, Ar-H), 6.56 (s, 1H, OH), 5.44 (s, 2H, CH_2_), 5.35 (d, *J* = 18.8 Hz, 1H, CH-H), 5.27 (d, *J* = 18.9 Hz, 1H, CH-H), 4.53 (p, *J* = 7.3 Hz, 1H, NHCH), 1.88 (m, 2H, CH_2_CH_3_), 1.52 (s, 9H, OCC_3_H_9_), 1.42 (d, *J* = 7.3 Hz, 3H, NHCHCH_3_), 0.89 (t, *J* = 7.3 Hz, 3H, CH_2_CH_3_); ^13^C NMR (151 MHz, DMSO-*d*_6_) *δ* 172.85, 171.78, 164.72, 157.17, 153.00, 150.50, 148.86, 145.42, 137.87, 131.22, 129.96, 128.85, 127.08, 126.12, 124.67, 120.04, 97.46, 81.50, 72.83, 65.70, 50.24, 49.55, 30.75, 28.20 (three), 17.17, 8.25; HRMS (ESI): calcd for C_28_H_29_N_3_O_7_ (M+H^+^) 520.2006, found 520.2078.

***(S)-methyl 2- ( (S)-4-ethyl-4-hydroxy-3,14-dioxo-3,4,12,14-tetrahydro-1H-pyrano[3',4': 6,7]indolizino[1,2-b]quinoline-11-carboxamido)-3-methylbutanoate (4o)***

Compound **4o** was synthesized as a yellow solid in 54% yield; ^1^H NMR (600 MHz, DMSO-*d*_6_) *δ* 9.23 (d, *J* = 7.9 Hz, 1H, NH), 8.25 (d, *J* = 8.5 Hz, 1H, Ar-H), 8.09 (d, *J* = 8.3 Hz, 1H, Ar-H), 7.93 (t, *J* = 7.6 Hz, 1H, Ar-H), 7.80 (t, *J* = 7.6 Hz, 1H, Ar-H), 7.39 (s, 1H, Ar-H), 6.56 (s, 1H, OH), 5.44 (s, 2H, CH_2_), 5.34 (d, *J* = 18.7 Hz, 1H, CH-H), 5.23 (d, *J* = 18.7 Hz, 1H, CH-H), 4.63 (dd, *J* = 7.9, 5.8 Hz, 1H, NHCH), 3.80 (s, 3H, OCH_3_), 2.26 (m, 1H, NHCHCHC_2_H_6_), 1.95 – 1.81 (m, 2H, CH_2_CH_3_), 1.04 (d, *J* = 6.8 Hz, 3H, NHCHCHCH_3_CH_3_), 0.97 (d, *J* = 6.8 Hz, 3H, NHCHCHCH_3_CH_3_), 0.89 (t, *J* = 7.3 Hz, 3H, CH_2_CH_3_); ^13^C NMR (151 MHz, DMSO-*d*_6_) *δ* 172.86, 172.00, 165.63, 157.14, 152.96, 150.52, 148.81, 145.40, 138.01, 131.20, 129.99, 128.99, 127.13, 125.86, 124.73, 120.04, 97.49, 72.84, 65.68, 58.41, 52.54, 50.19, 30.76, 29.96, 19.59, 18.54, 8.23; HRMS (ESI): calcd for C_27_H_27_N_3_O_7_ (M+H^+^) 506.1849, found 506.1922.

***(S)-methyl 2- ( (S)-4-ethyl-4-hydroxy-3,14-dioxo-3,4,12,14-tetrahydro-1H-pyrano[3',4': 6,7]indolizino[1,2-b]quinoline-11-carboxamido)-3,3-dimethylbutanoate (4p)***

Compound **4p** was synthesized as a yellow solid in 50% yield; ^1^H NMR (600 MHz, DMSO-*d*_6_) *δ* 9.17 (d, *J* = 8.1 Hz, 1H, NH), 8.26 (d, *J* = 8.5 Hz, 1H, Ar-H), 8.04 (d, *J* = 8.4 Hz, 1H, Ar-H), 7.93 (t, *J* = 7.7 Hz, 1H, Ar-H), 7.80 (t, *J* = 7.6 Hz, 1H, Ar-H), 7.39 (s, 1H, Ar-H), 6.57 (s, 1H, OH), 5.44 (s, 2H, CH_2_), 5.31 (d, *J* = 18.6 Hz, 1H, CH-H), 5.21 (d, *J* = 18.7 Hz, 1H, CH-H), 4.54 (d, *J* = 8.1 Hz, 1H, NHCH), 3.80 (s, 3H, OCH_3_), 1.95 – 1.82 (m, 2H, CH_2_CH_3_), 1.07 (s, 9H, CC_3_H_9_), 0.89 (t, *J* = 7.3 Hz, 3H, CH_2_CH_3_); ^13^C NMR (151 MHz, DMSO-*d*_6_) *δ* 172.88, 171.46, 165.58, 157.15, 152.97, 150.54, 148.80, 145.42, 138.00, 131.21, 130.01, 127.23, 125.81, 124.82, 120.05, 97.48, 72.84, 65.68, 61.86, 52.29, 50.17, 33.92, 30.74, 27.13 (three), 8.22; HRMS (ESI): calcd for C_28_H_29_N_3_O_7_ (M+H^+^) 520.2006, found 520.2079.

***(******S)-methyl 2- ( (S)-4-ethyl-4-hydroxy-3,14-dioxo-3,4,12,14-tetrahydro-1H-pyrano[3',4': 6,7]indolizino[1,2-b]quinoline-11-carboxamido)-3-phenylpropanoate (4q)***

Compound **4q** was synthesized as a yellow solid in 50% yield; ^1^H NMR (600 MHz, DMSO-*d*_6_) *δ* 9.44 (d, *J* = 8.3 Hz, 1H, NH), 8.21 (d, *J* = 8.5 Hz, 1H, Ar-H), 7.89 (m, 1H, Ar-H), 7.71 – 7.63 (m, 2H, Ar-H), 7.41 – 7.28 (m, 6H, Ar-H), 6.56 (s, 1H, OH), 5.45 (s, 2H, CH_2_), 5.11 (d, *J* = 18.9 Hz, 1H, CH-H), 5.07 – 4.97 (m, 2H, CH-H, NHCH), 3.81 (s, 3H, OCH_3_), 3.33 (dd, *J* = 14.0, 4.6 Hz, 1H, NHCHCH-H), 2.98 (dd, *J* = 14.0, 11.2 Hz, 1H, NHCHCH-H), 1.88 (m, 2H, CH_2_CH_3_), 0.88 (t, J = 7.3 Hz, 3H, CH_2_CH_3_); ^13^C NMR (151 MHz, DMSO-*d*_6_) *δ* 172.87, 171.94, 165.13, 157.13, 152.92, 150.46, 148.74, 145.33, 137.85, 137.62, 131.20, 129.85, 129.60 (two), 128.91 (two), 128.75, 127.26, 126.76, 125.89, 124.42, 120.05, 97.48, 72.83, 65.71, 54.10, 52.86, 49.96, 36.68, 30.74, 8.23; HRMS (ESI): calcd for C_31_H_27_N_3_O_7_ (M+H^+^) 552.1849, found 552.1795.

**4. Copies of NMR spectra**


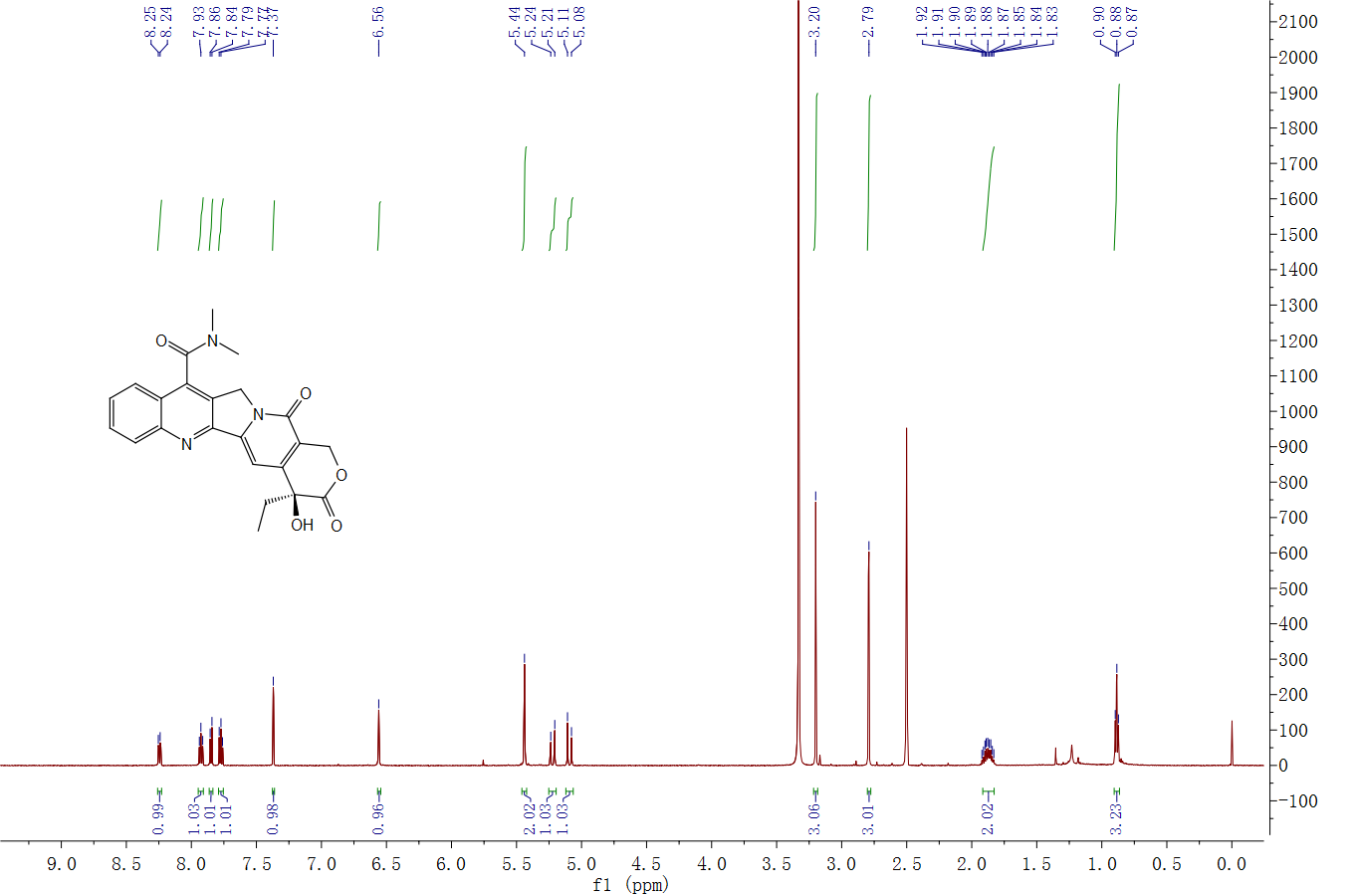


**Supplementary Figure 5**. ^1^H NMR spectrum of **4a**

**
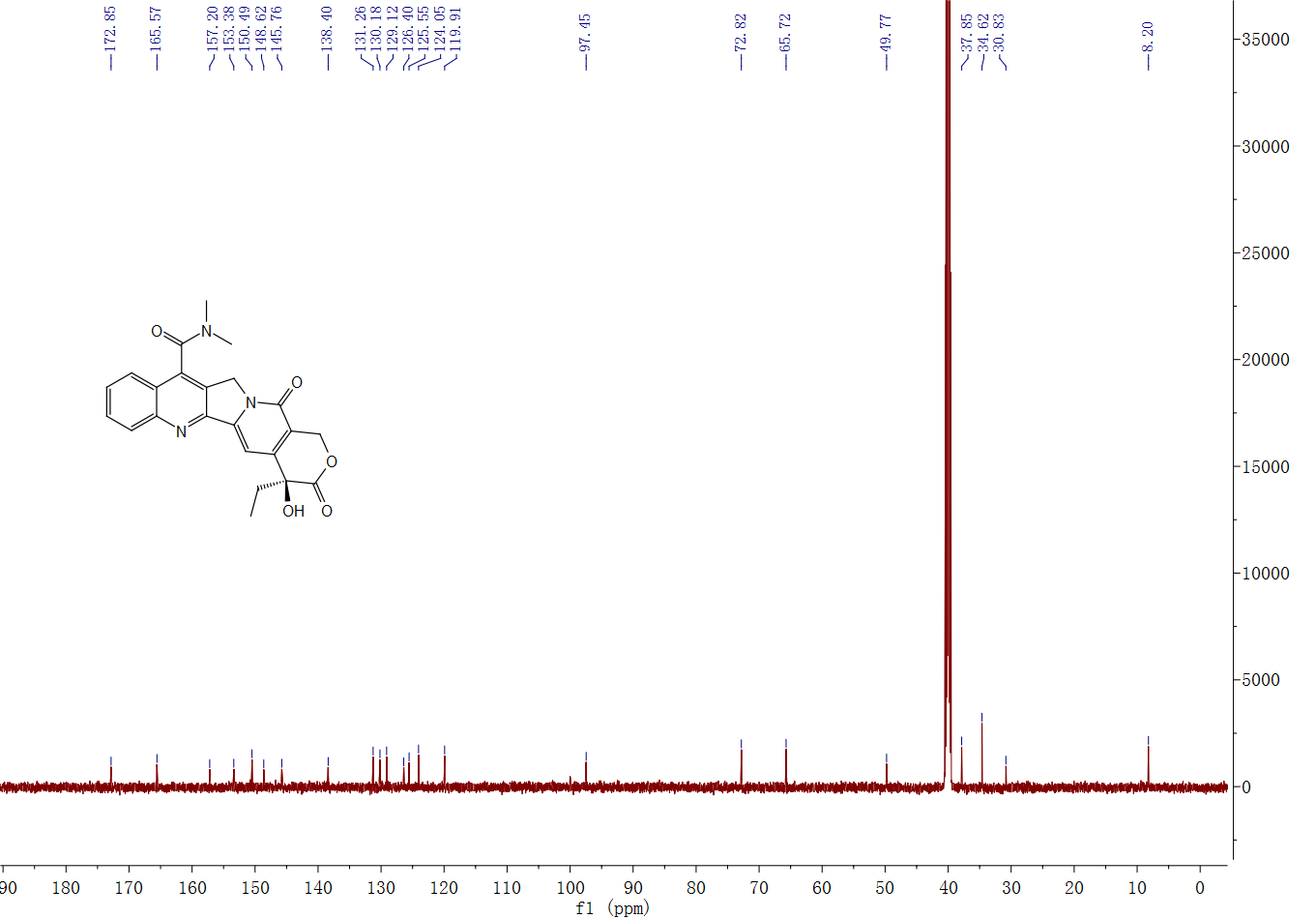
**

**Supplementary Figure 6**. ^13^C NMR spectrum of **4a**


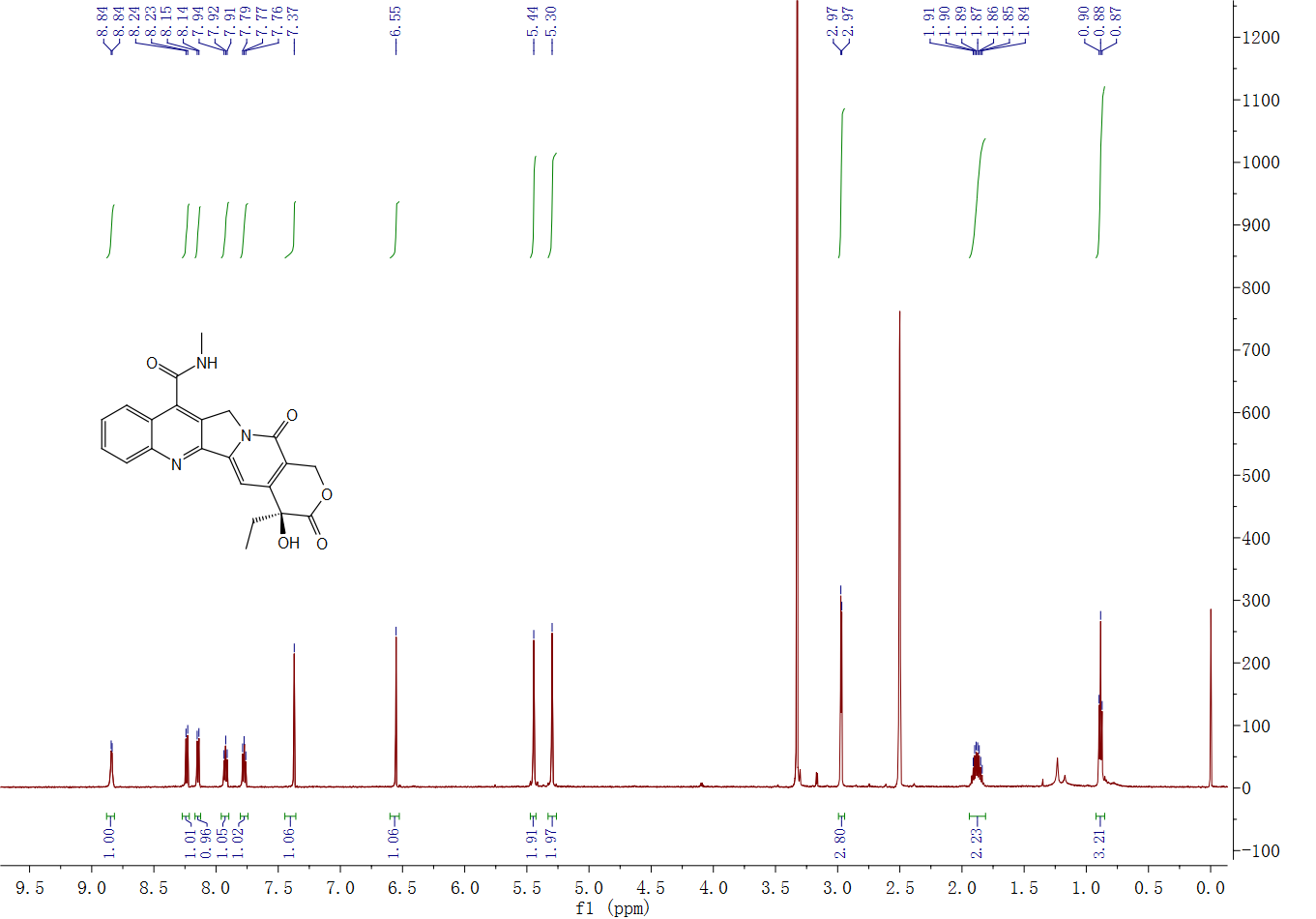


**Supplementary Figure 7**. ^1^H NMR spectrum of **4b**

**
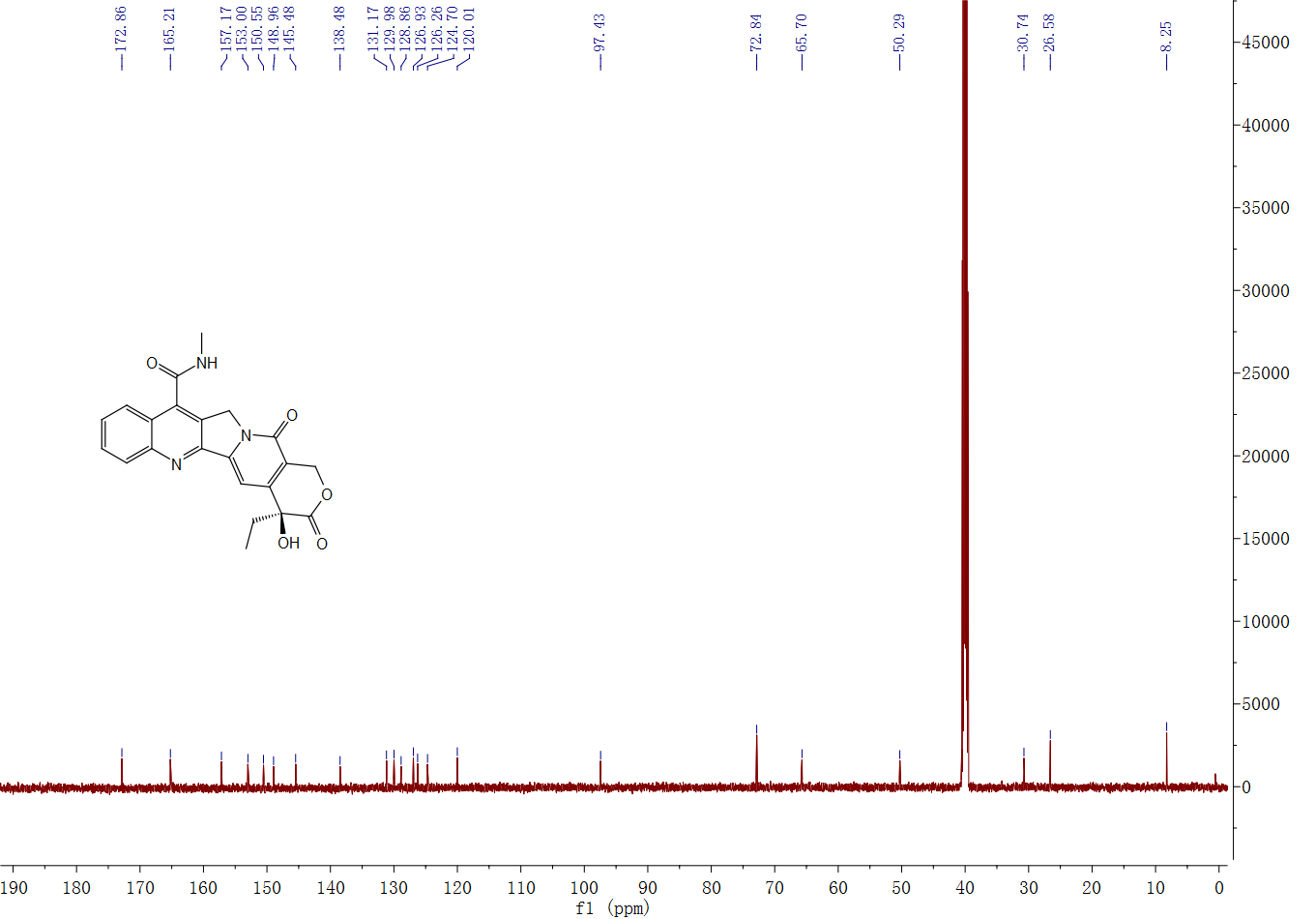
**

**Supplementary Figure 8**. ^13^C NMR spectrum of **4b**


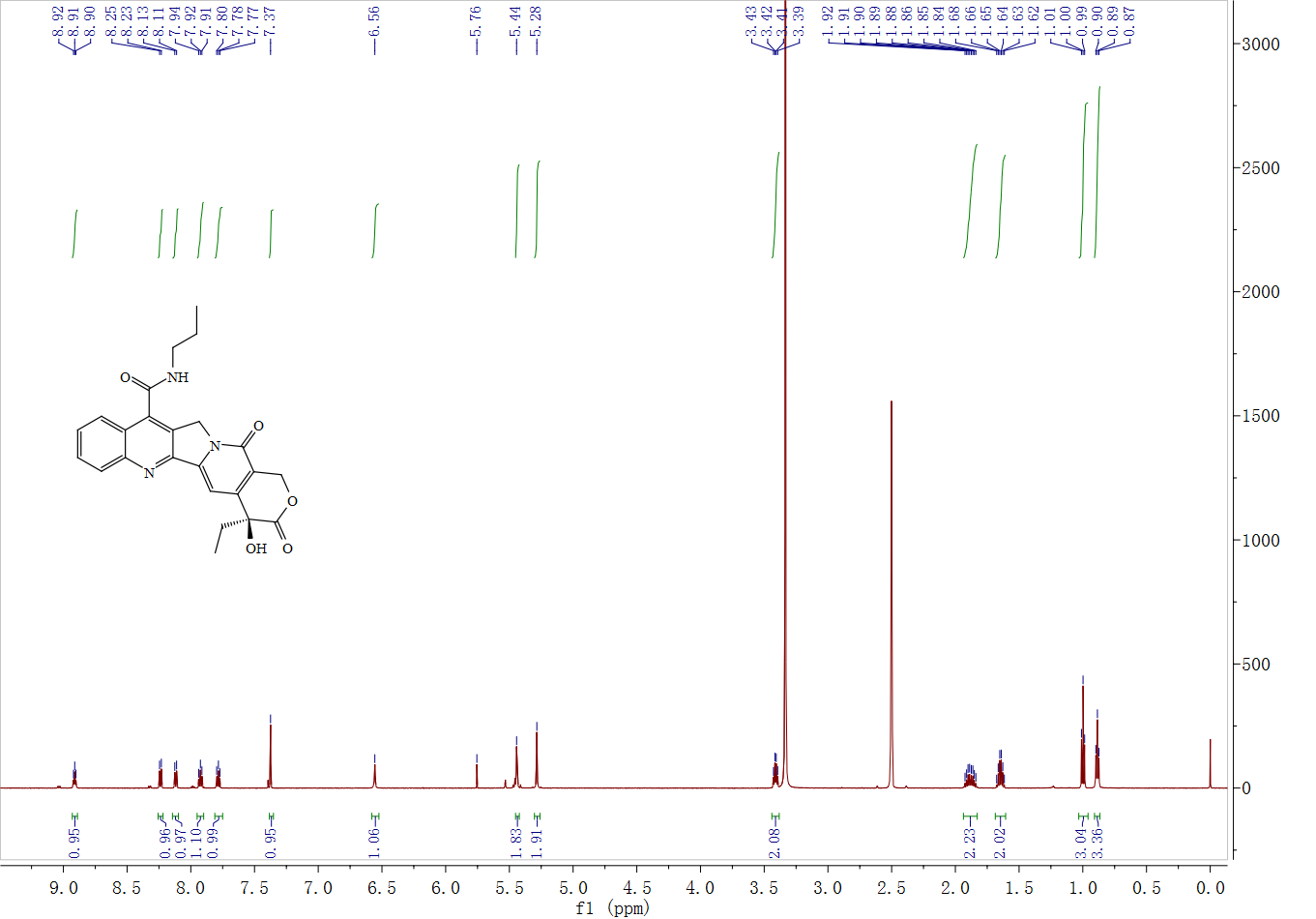


**Supplementary Figure 9**. ^1^H NMR spectrum of **4c**

**
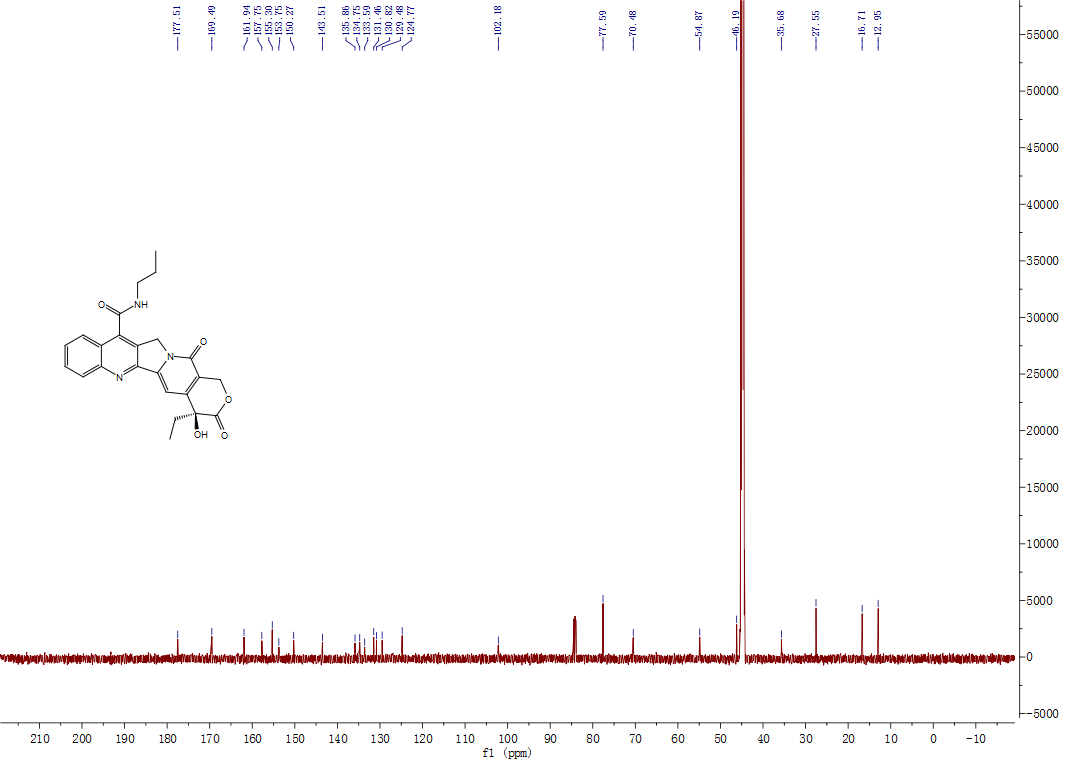
**

**Supplementary Figure 10**. ^13^C NMR spectrum of **4c**


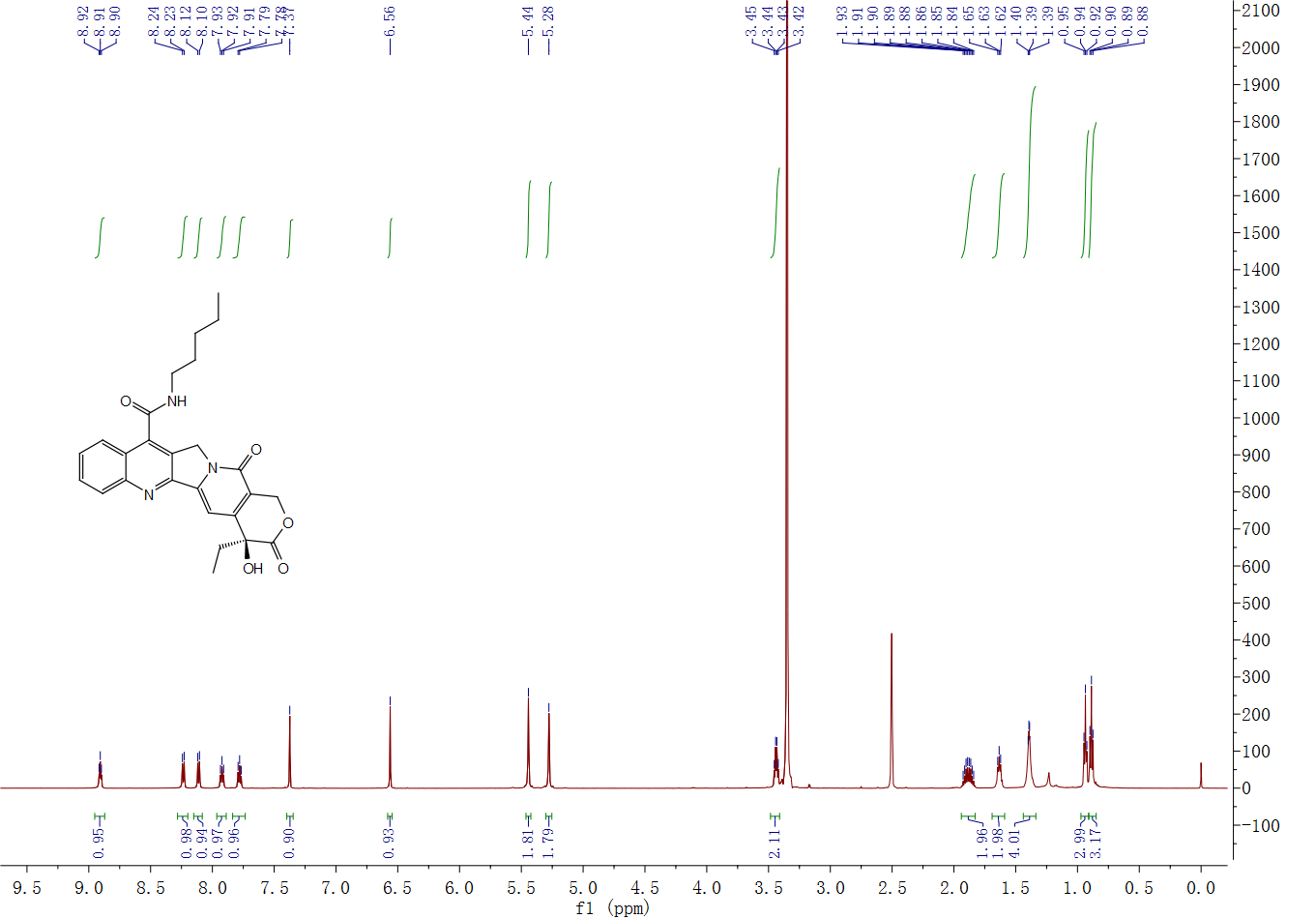


**Supplementary Figure 11**. ^1^H NMR spectrum of **4d**

**
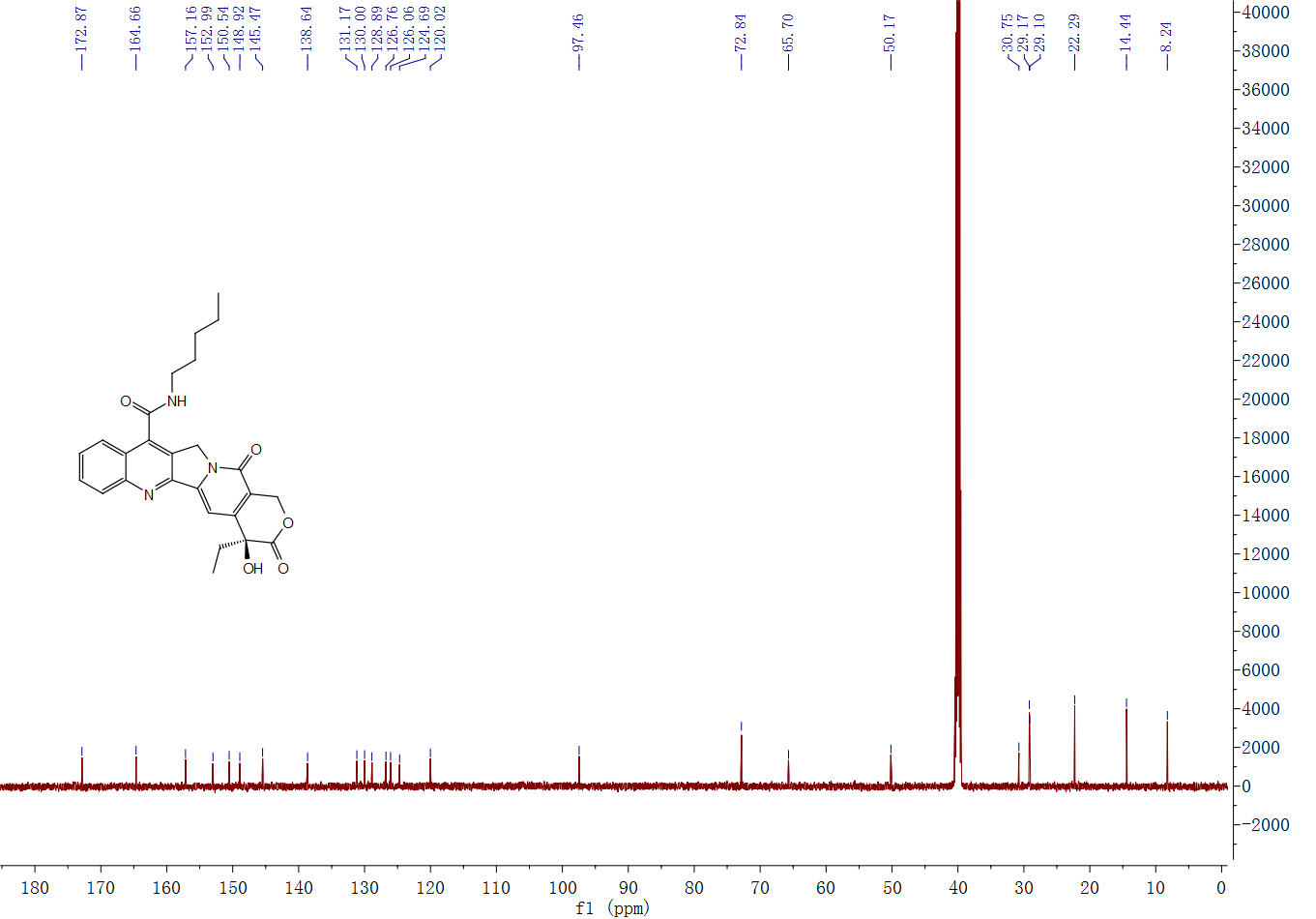
**

**Supplementary Figure 12**. ^13^C NMR spectrum of **4d**


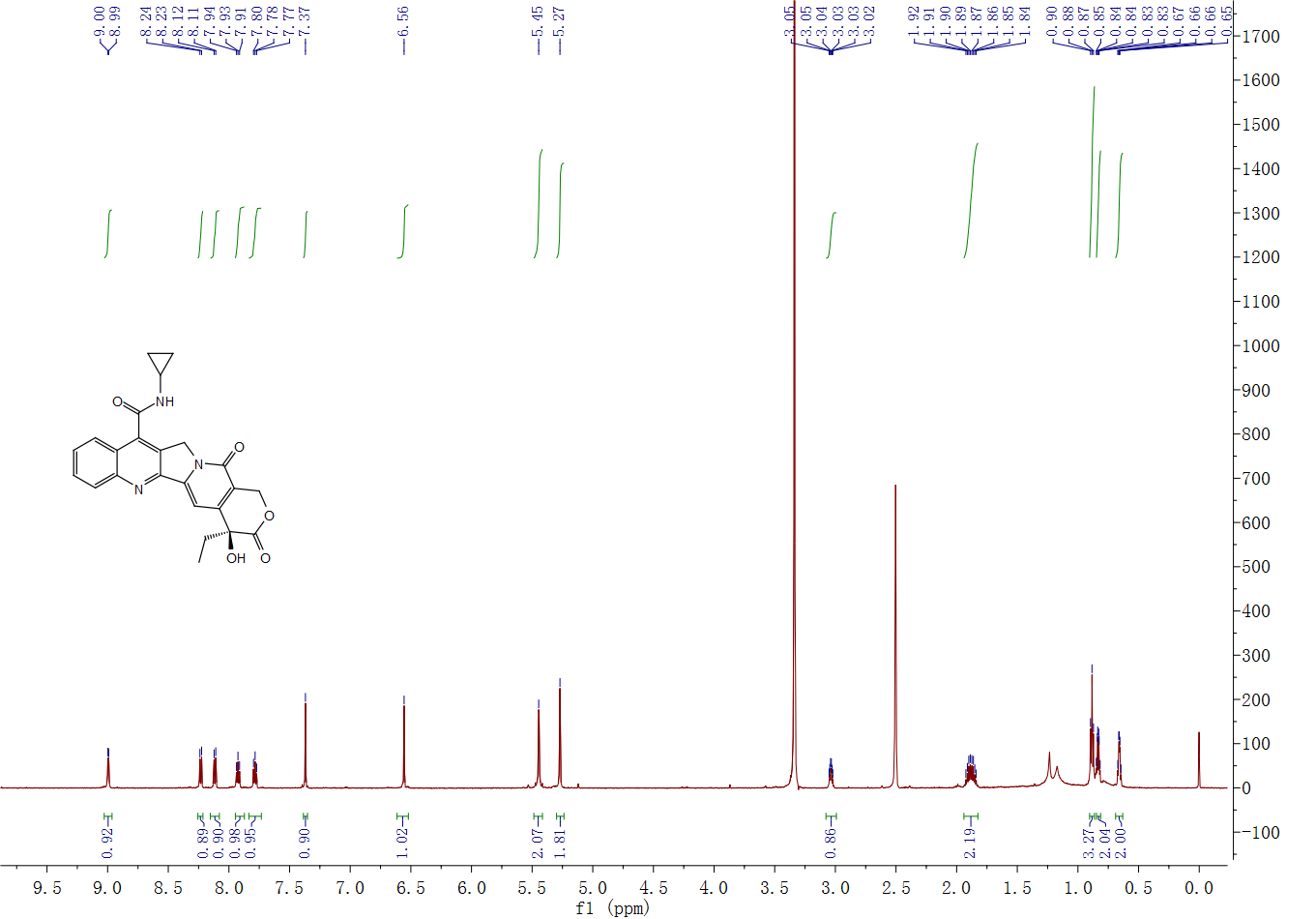


**Supplementary Figure 13**. ^1^H NMR spectrum of **4e**

**
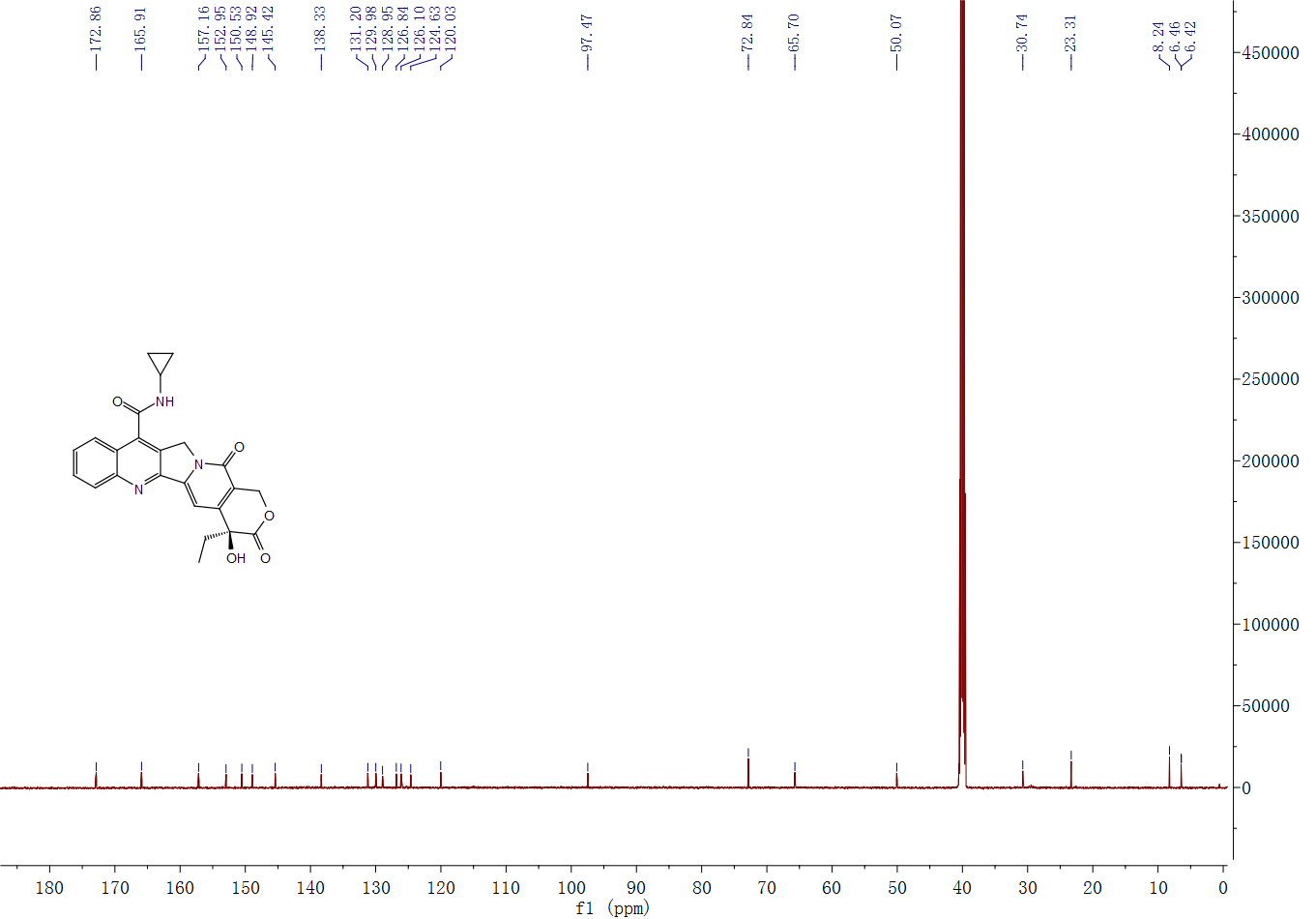
**

**Supplementary Figure 14**. ^13^C NMR spectrum of **4e**


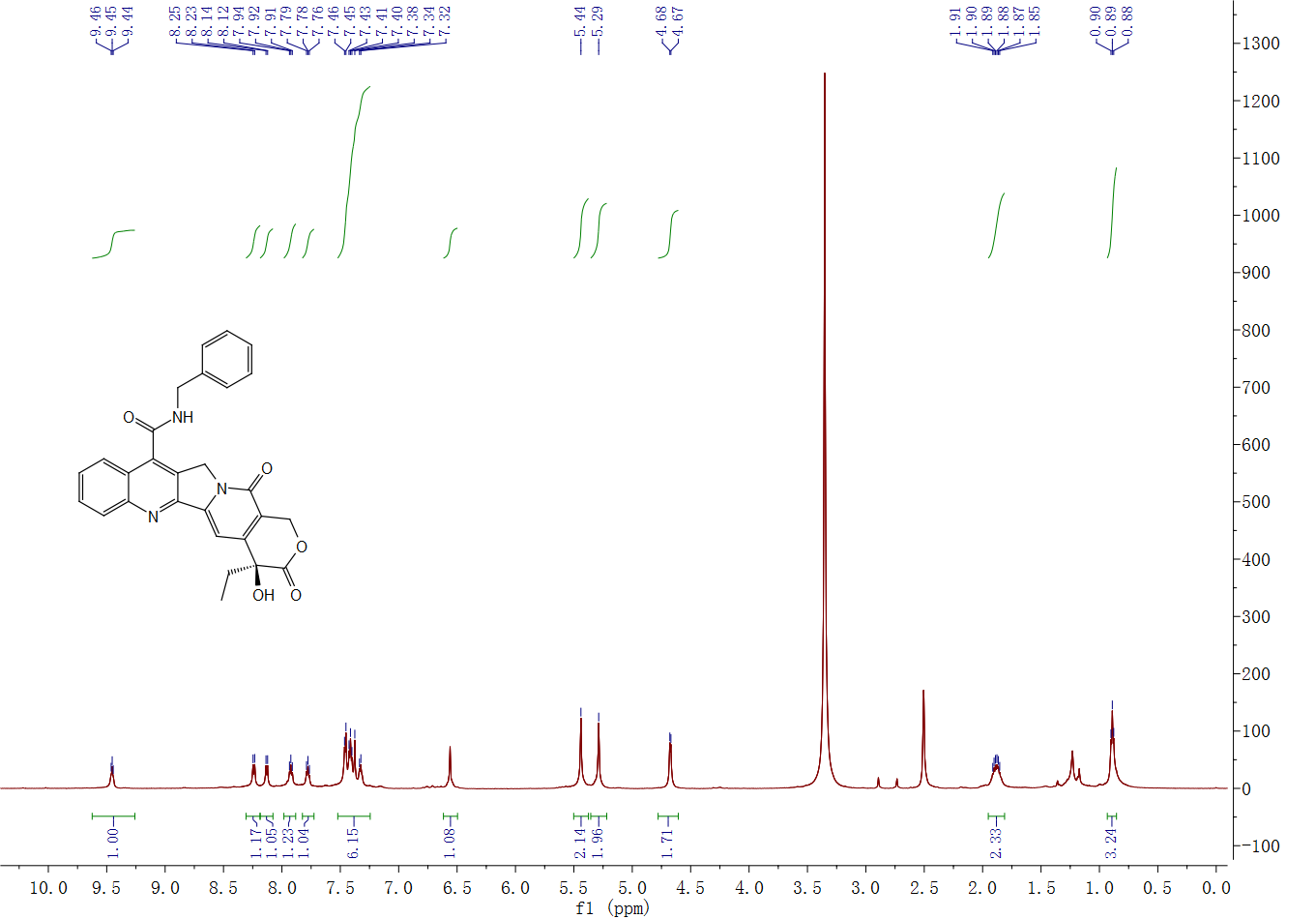


**Supplementary Figure 15**. ^1^H NMR spectrum of **4f**

**
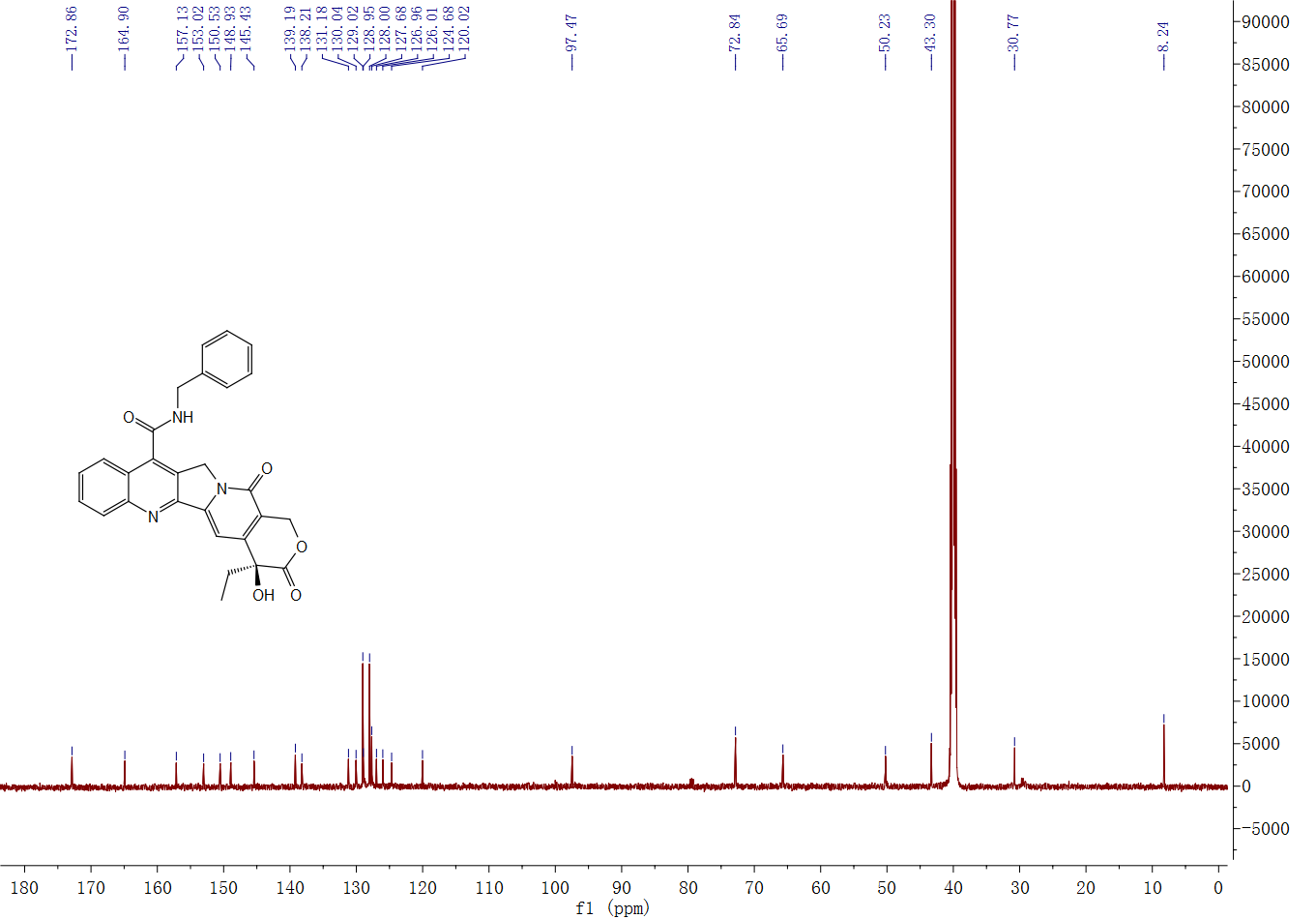
**

**Supplementary Figure 16**. ^13^C NMR spectrum of **4f**


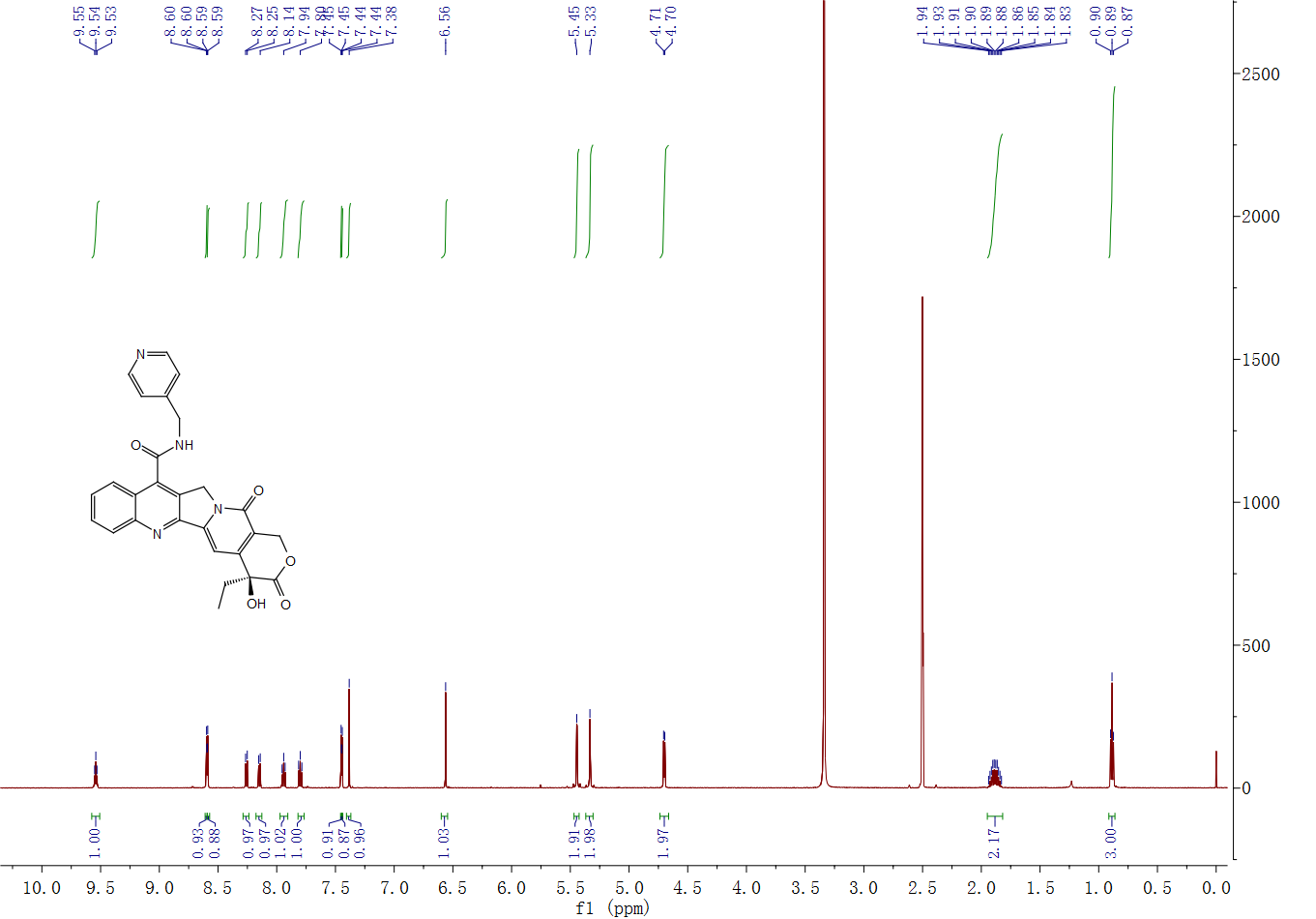


**Supplementary Figure 17**. ^1^H NMR spectrum of **4g**

**
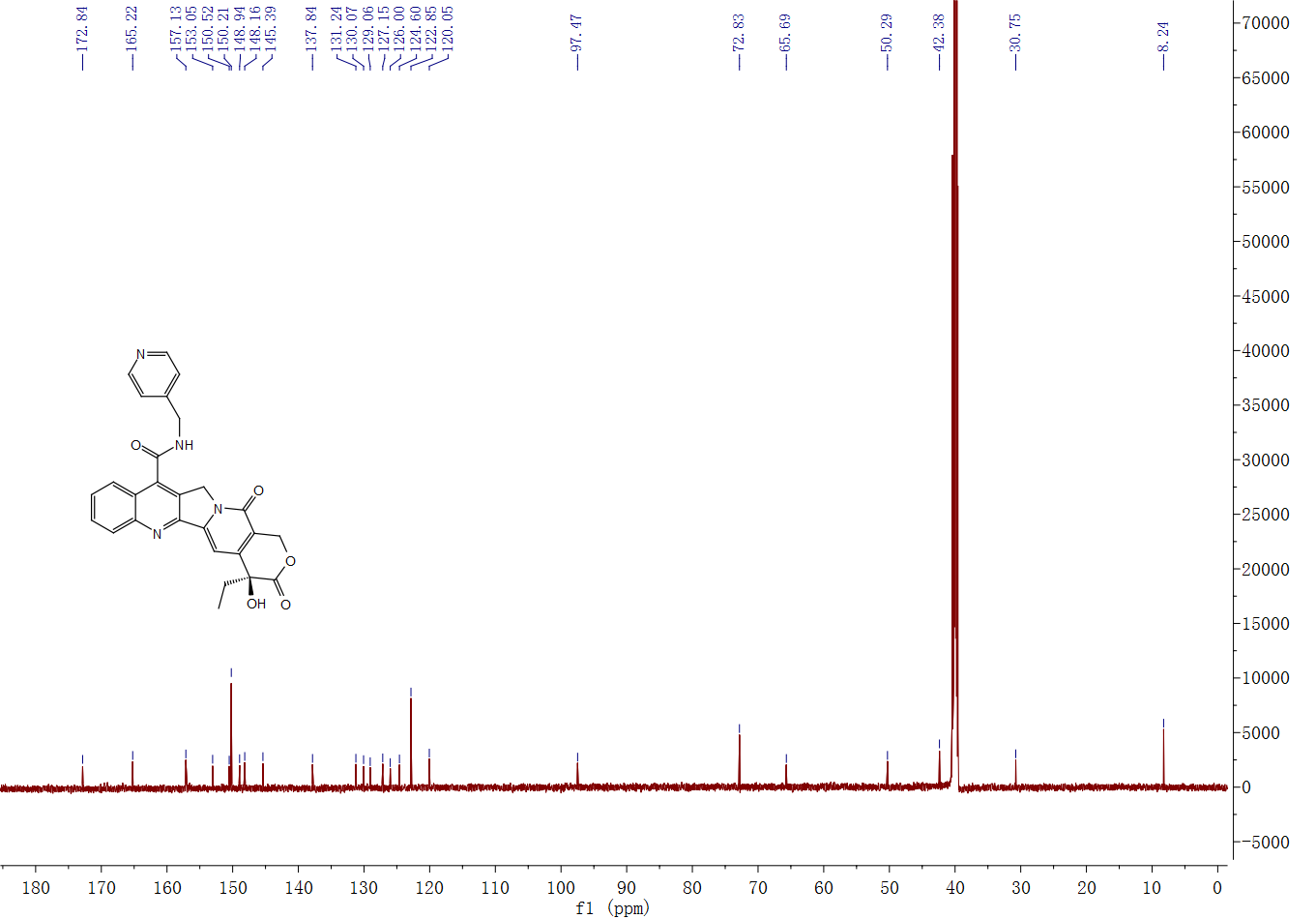
**

**Supplementary Figure 18**. ^13^C NMR spectrum of **4g**


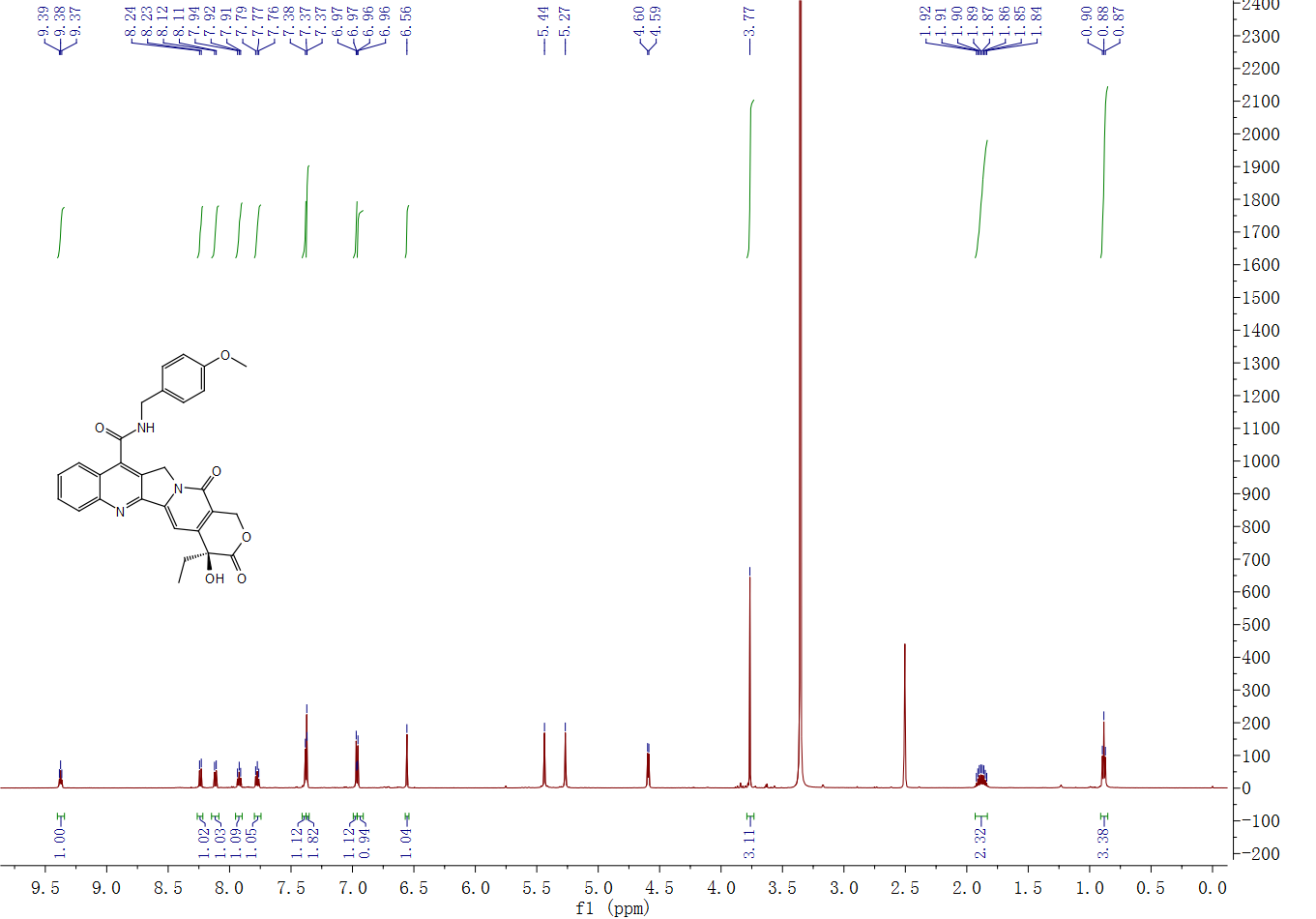


**Supplementary Figure 19**. ^1^H NMR spectrum of **4h**

**
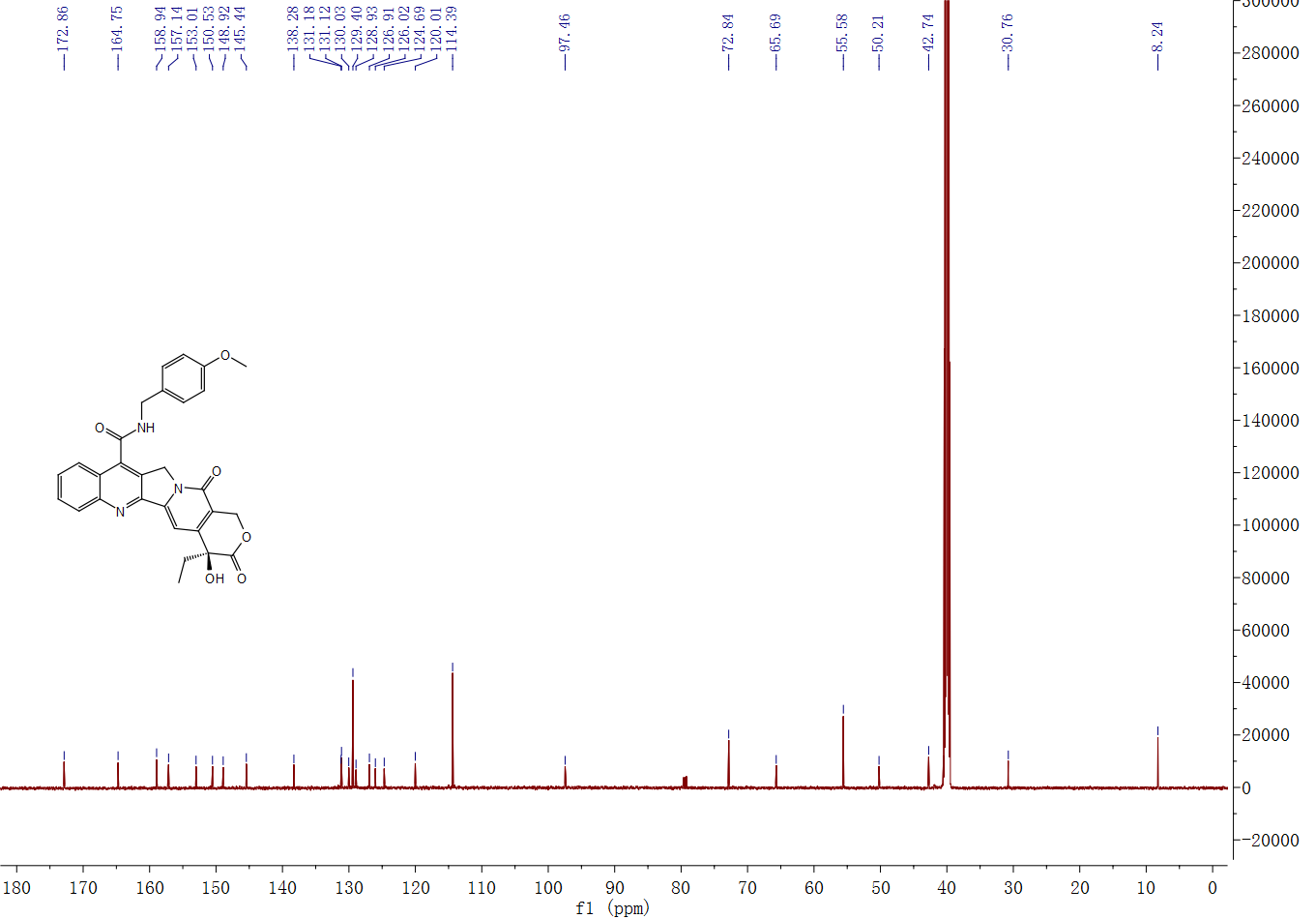
**

**Supplementary Figure 20**. ^13^C NMR spectrum of **4h**


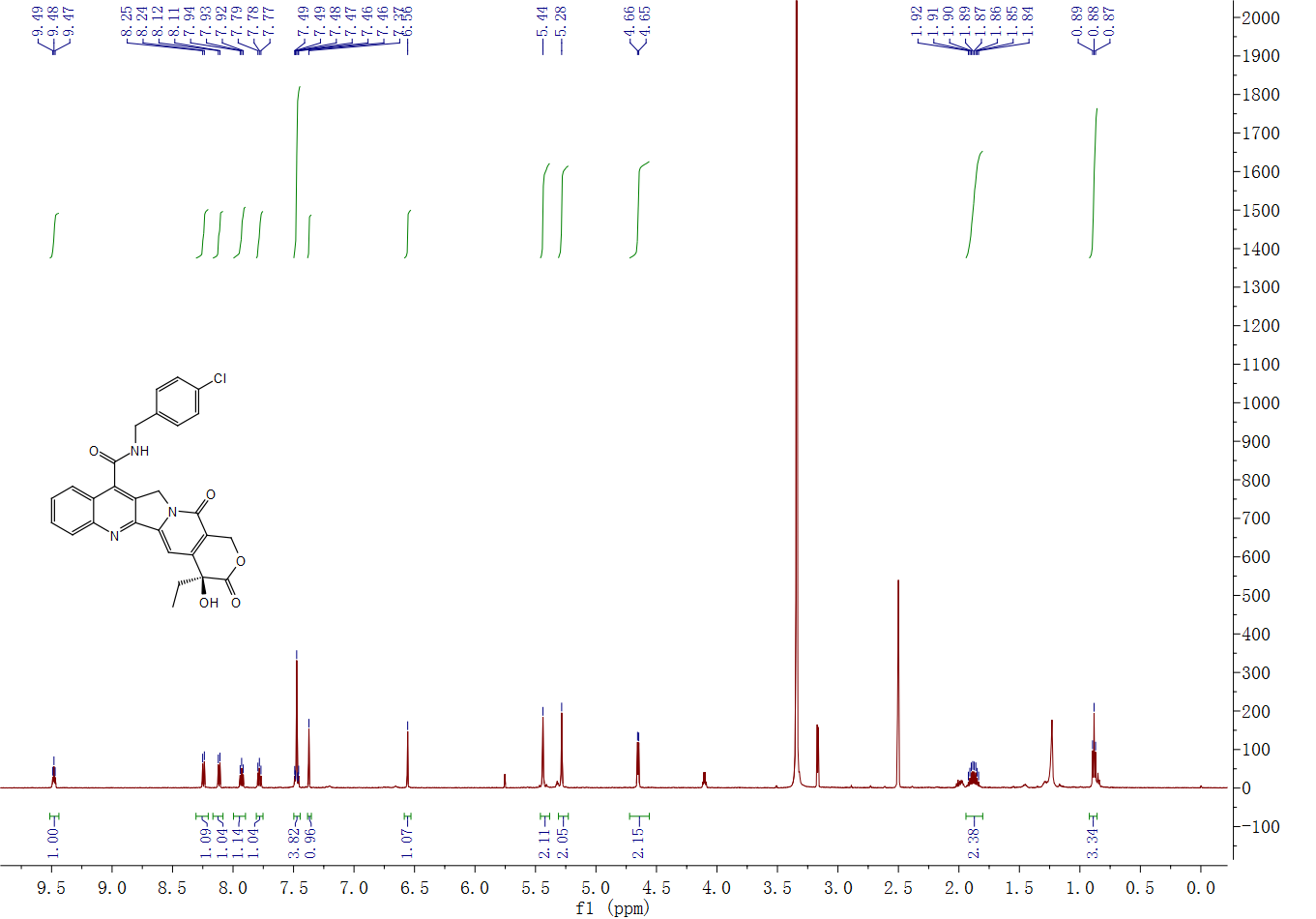


**Supplementary Figure 21**. ^1^H NMR spectrum of **4i**

**
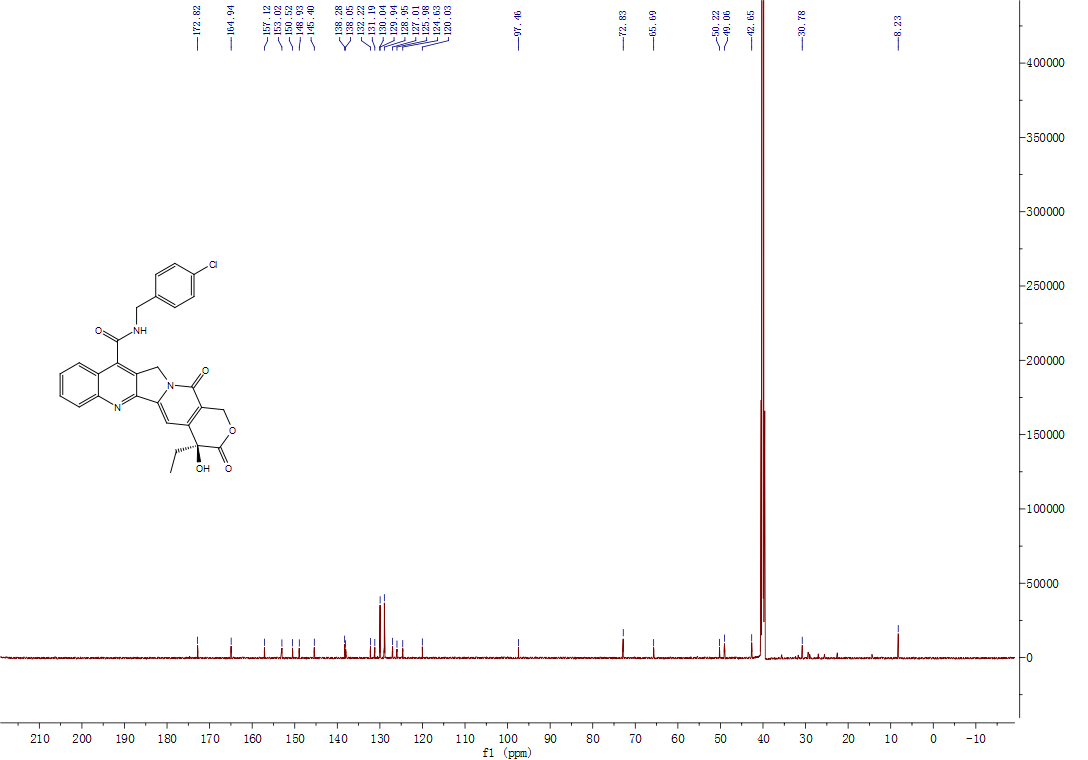
**

**Supplementary Figure 22**. ^13^C NMR spectrum of **4i**


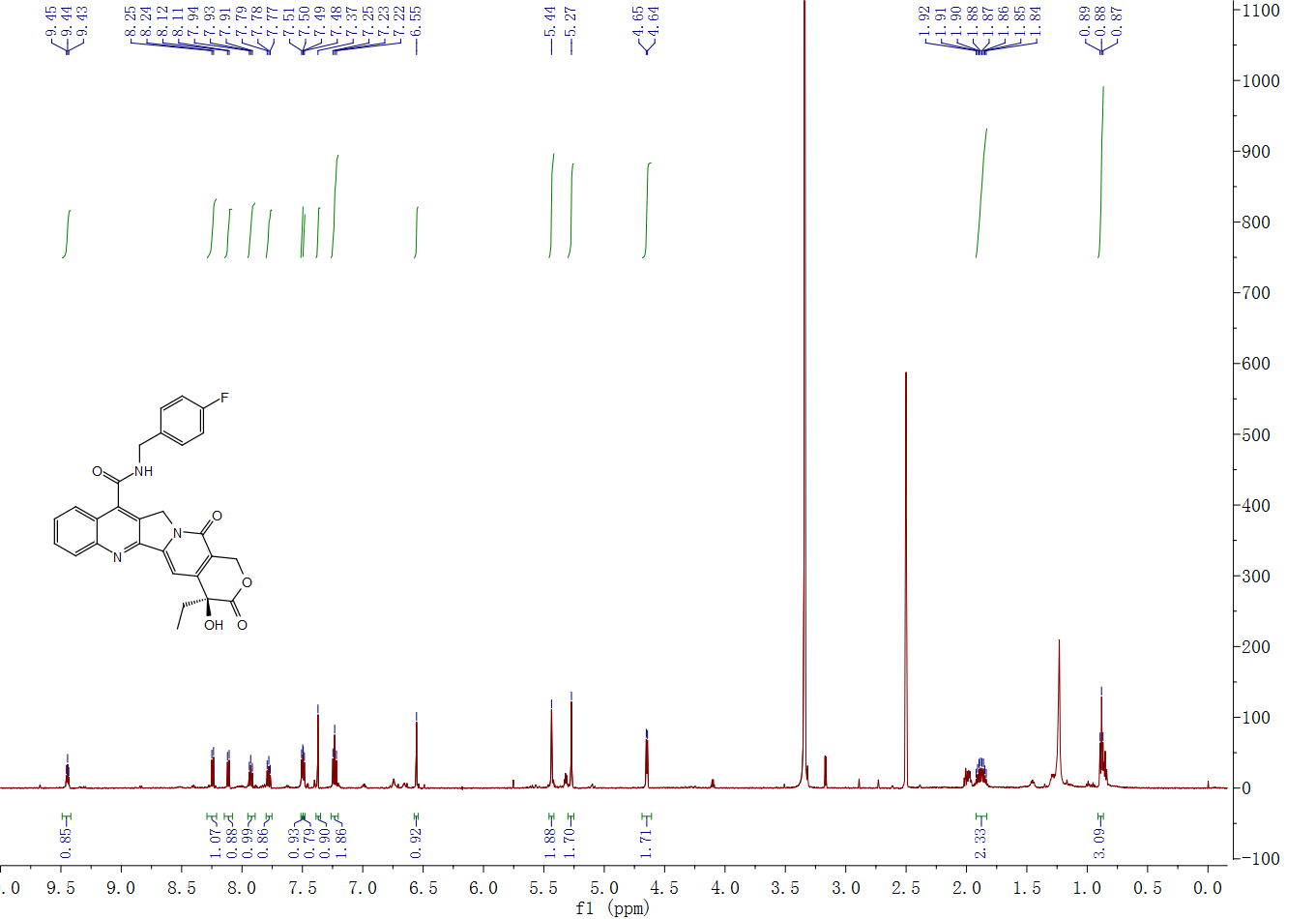


**Supplementary Figure 23**. ^1^H NMR spectrum of **4j**

**
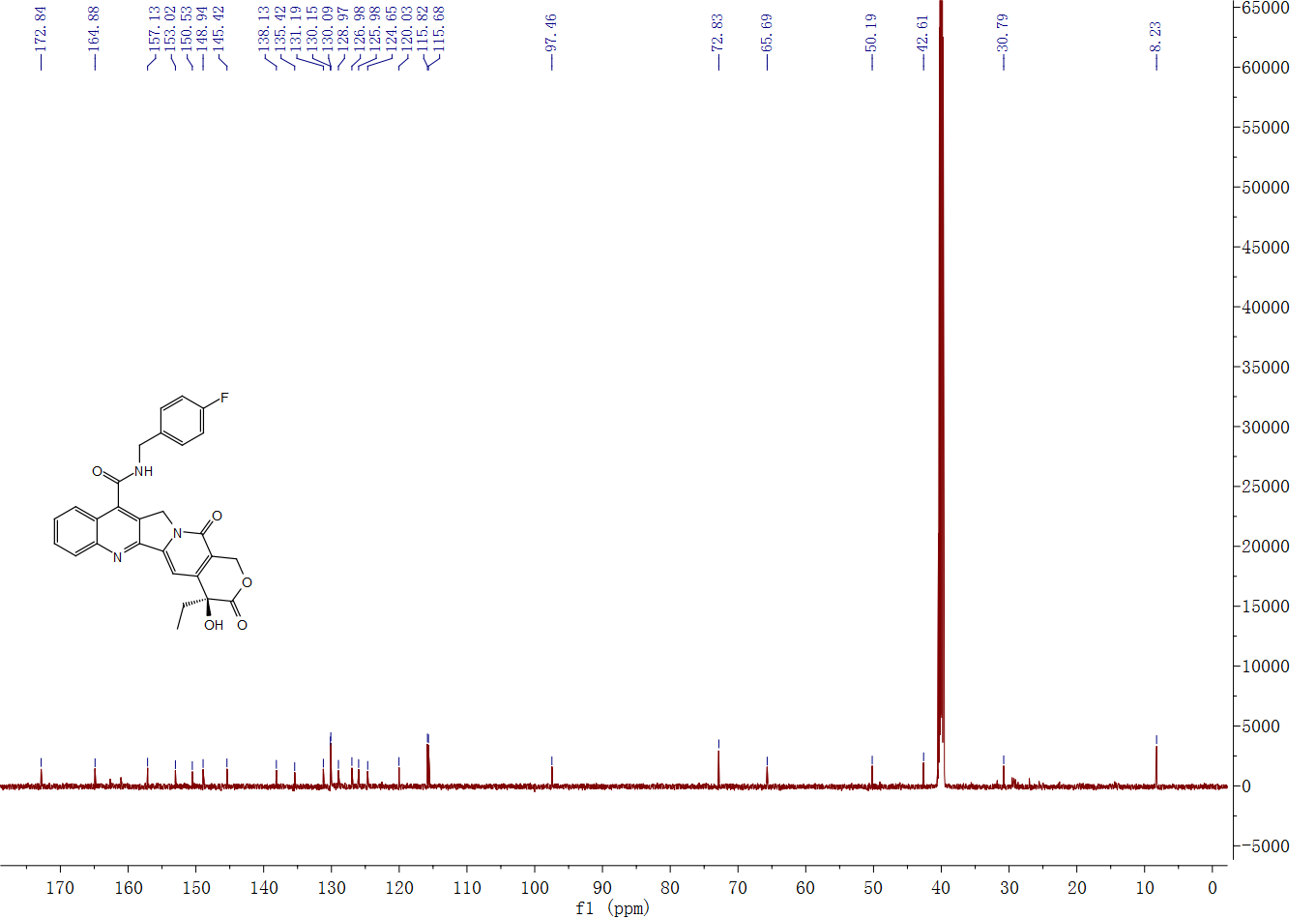
**

**Supplementary Figure 24**. ^13^C NMR spectrum of **4j**


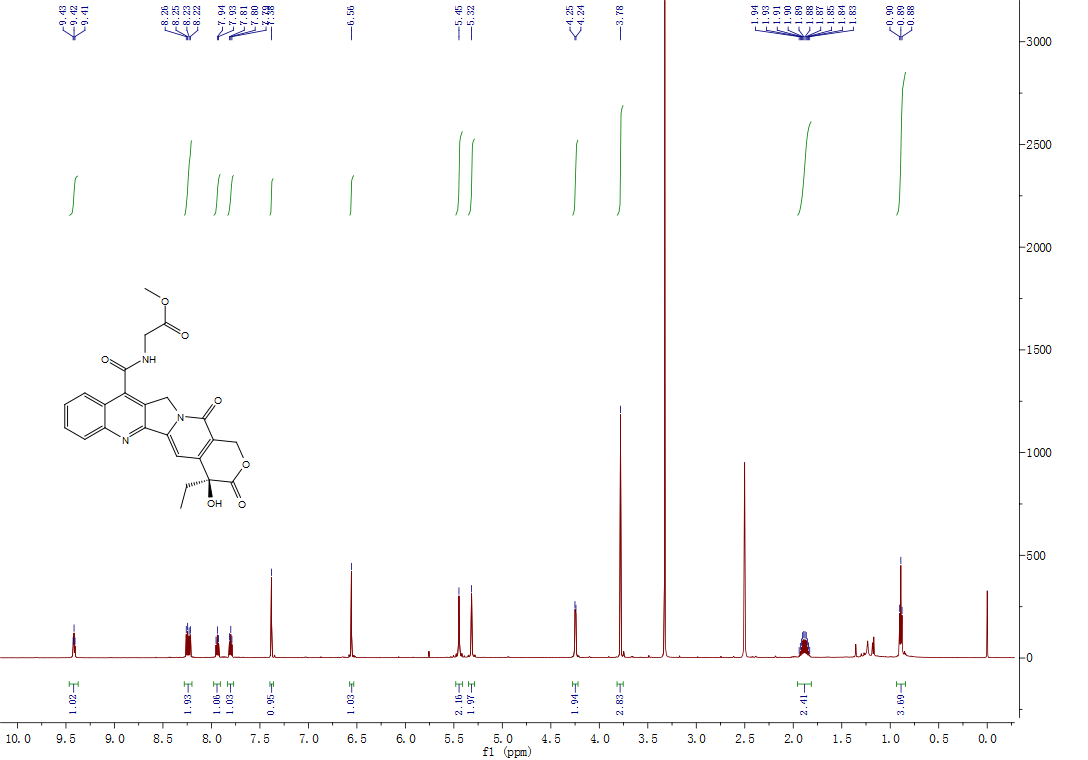


**Supplementary Figure 25**. ^1^H NMR spectrum of **4k**

**
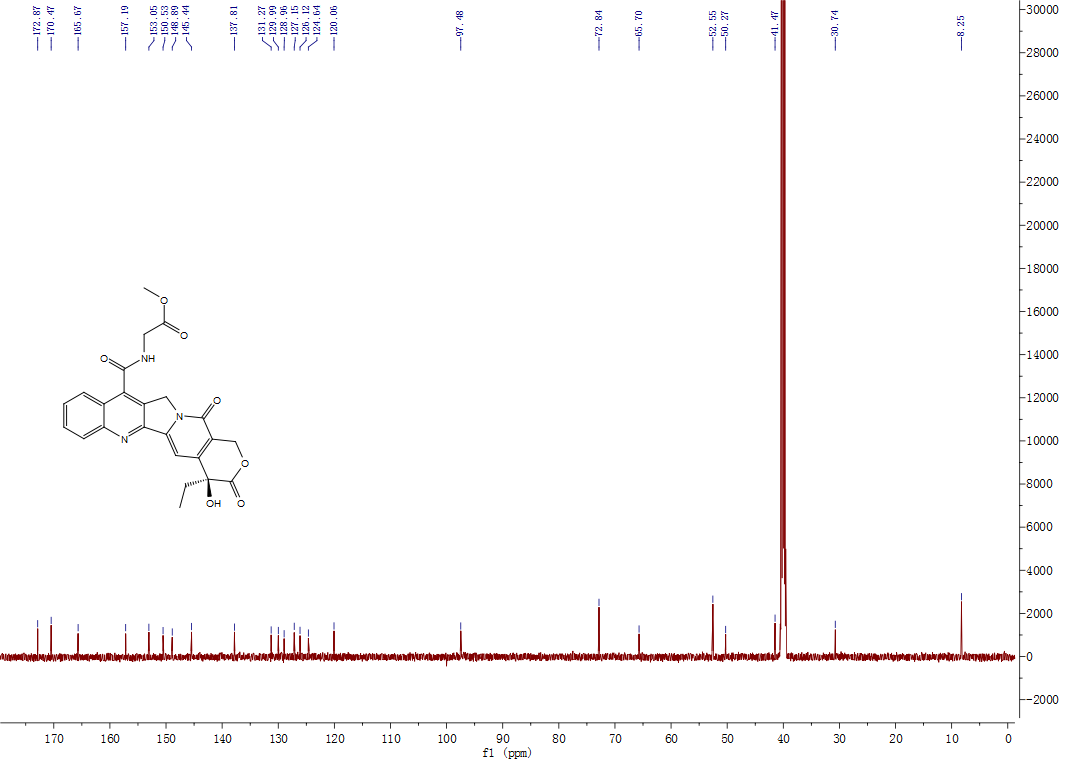
**

**Supplementary Figure 26**. ^13^C NMR spectrum of **4k**


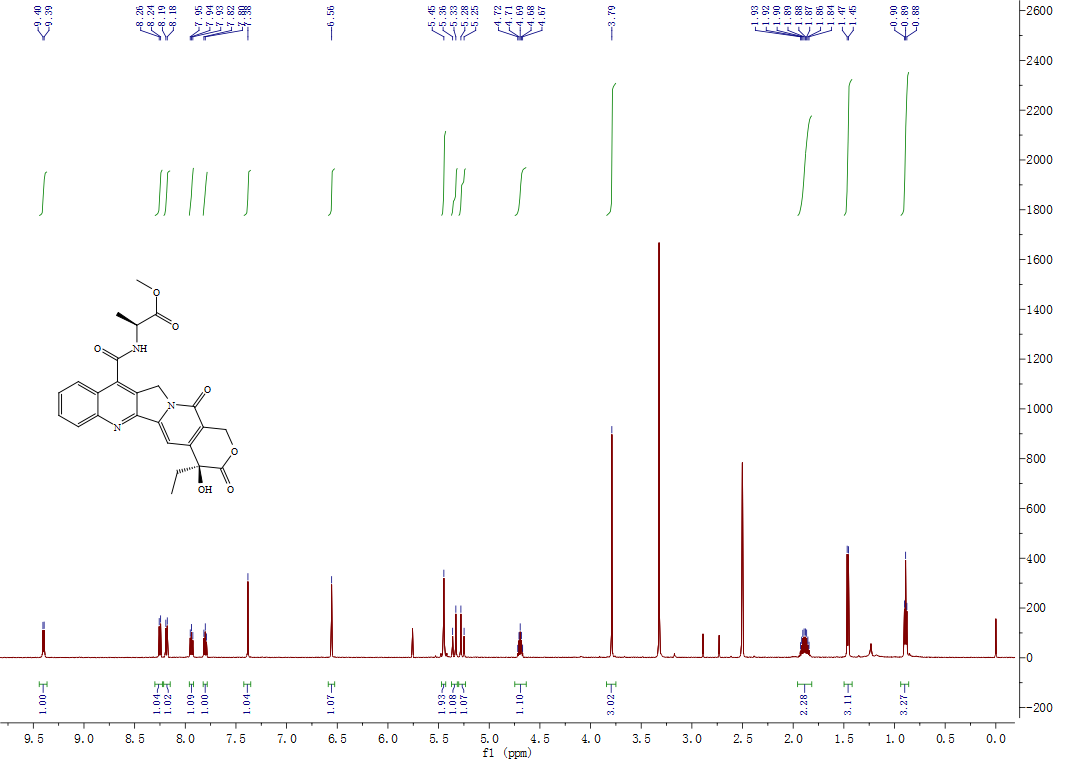


**Supplementary Figure 27**. ^1^H NMR spectrum of **4l**

**
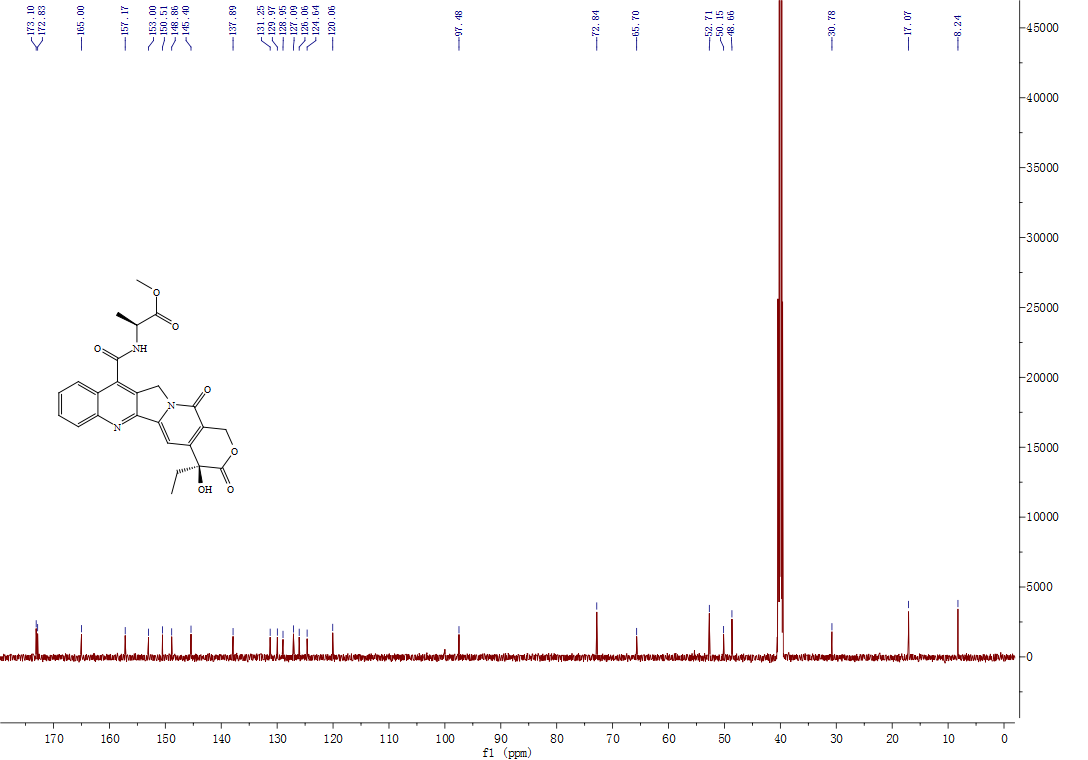
**

**Supplementary Figure 28**. ^13^C NMR spectrum of **4l**


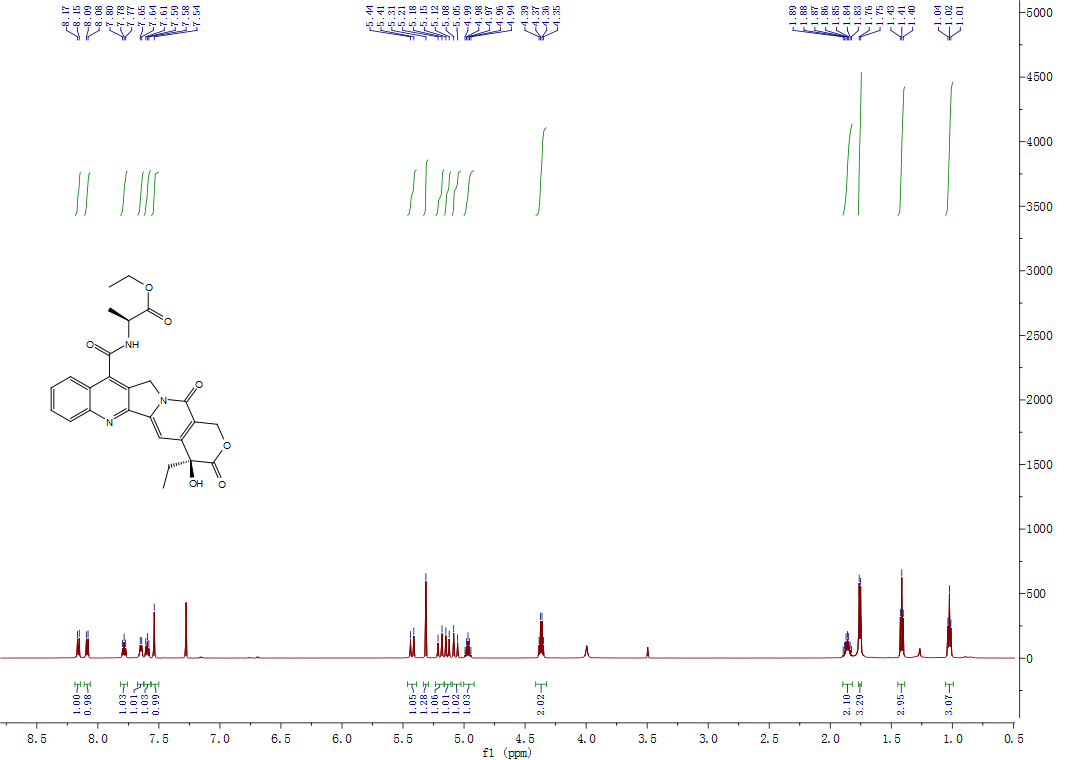


**Supplementary Figure 29**. ^1^H NMR spectrum of **4m**

**
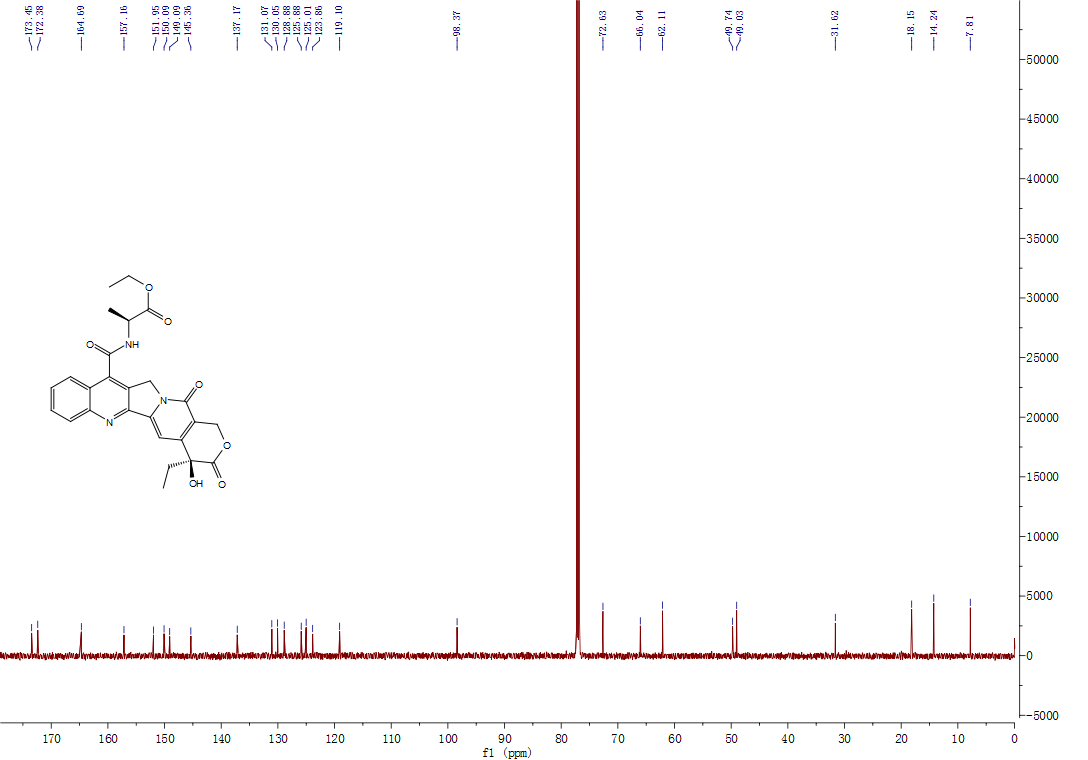
**

**Supplementary Figure 30**. ^13^C NMR spectrum of **4m**


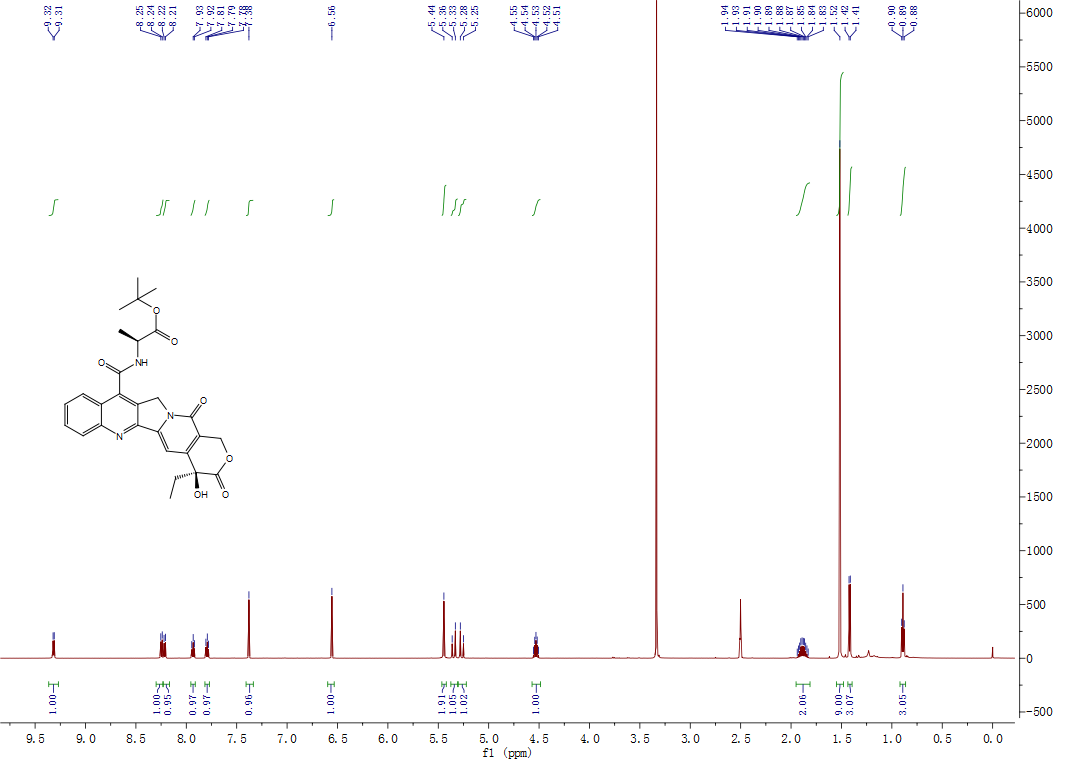


**Supplementary Figure 31**. ^1^H NMR spectrum of **4n**

**
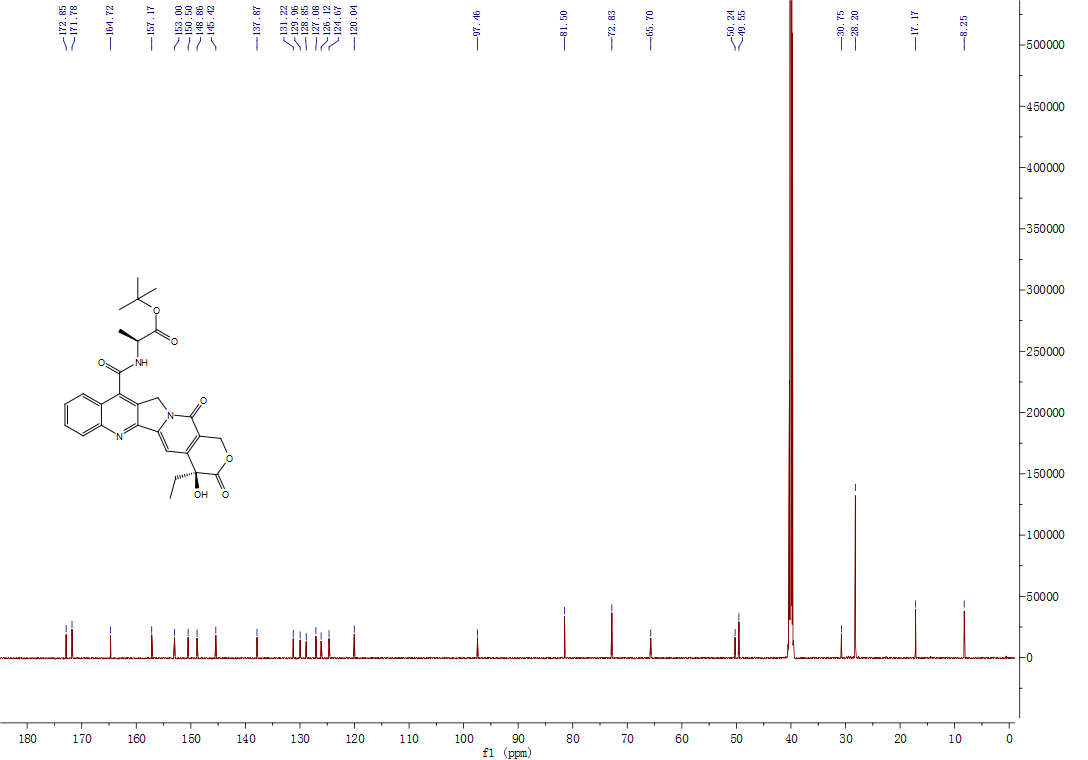
**

**Supplementary Figure 32**. ^13^C NMR spectrum of **4n**


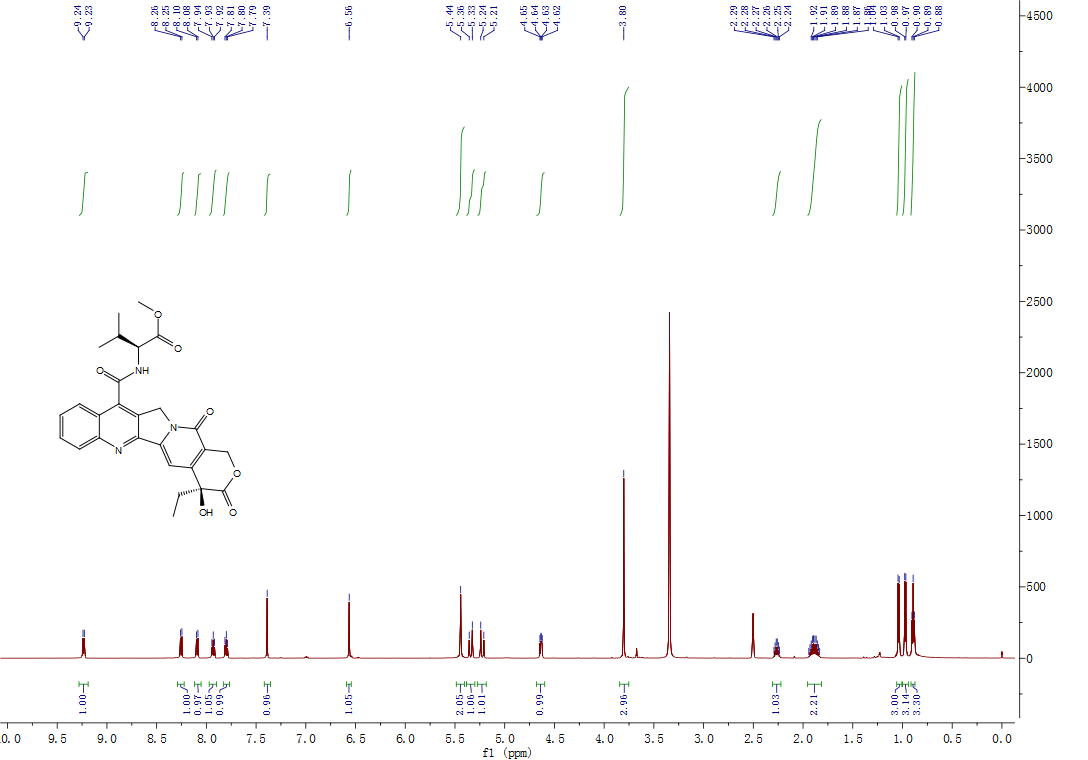


**Supplementary Figure 33**. ^1^H NMR spectrum of **4o**

**
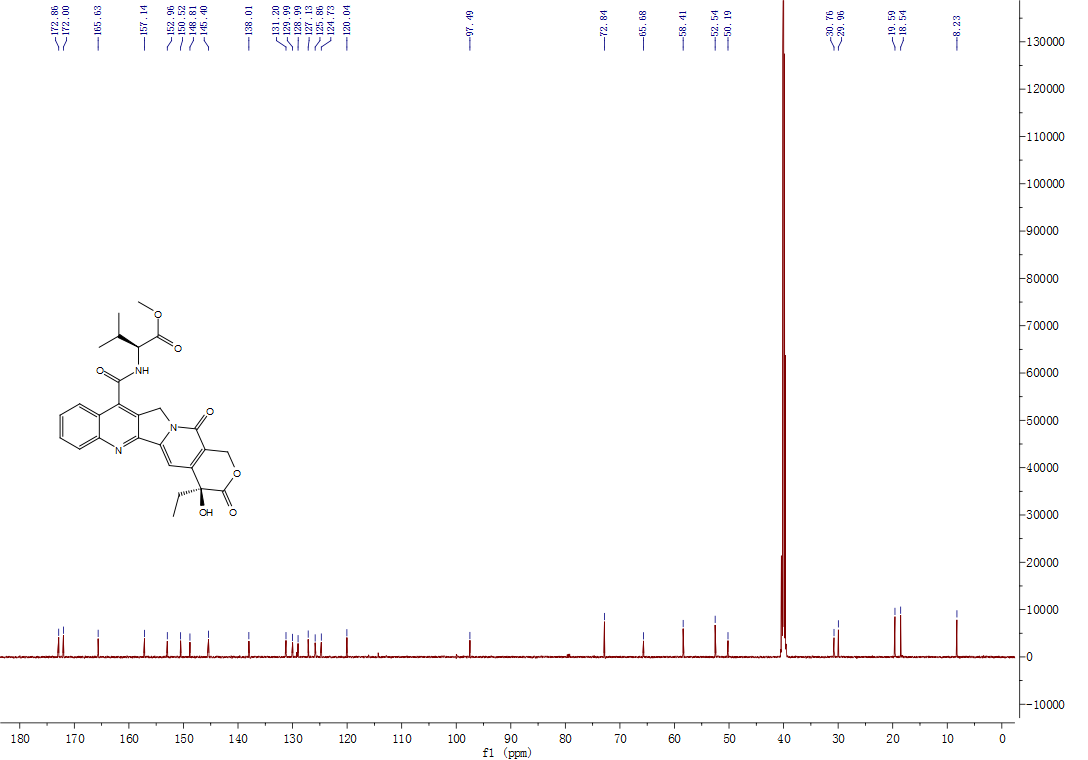
**

**Supplementary Figure 34**. ^13^C NMR spectrum of **4o**

**
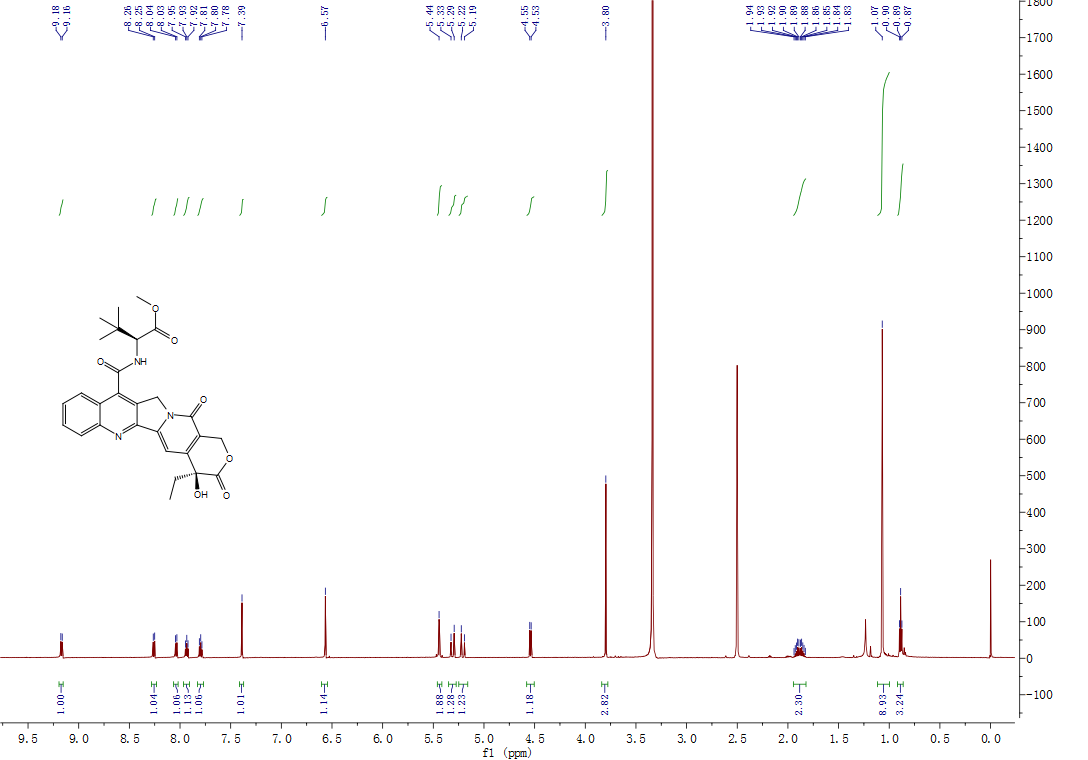
**

**Supplementary Figure 35**. ^1^H NMR spectrum of **4p**

**
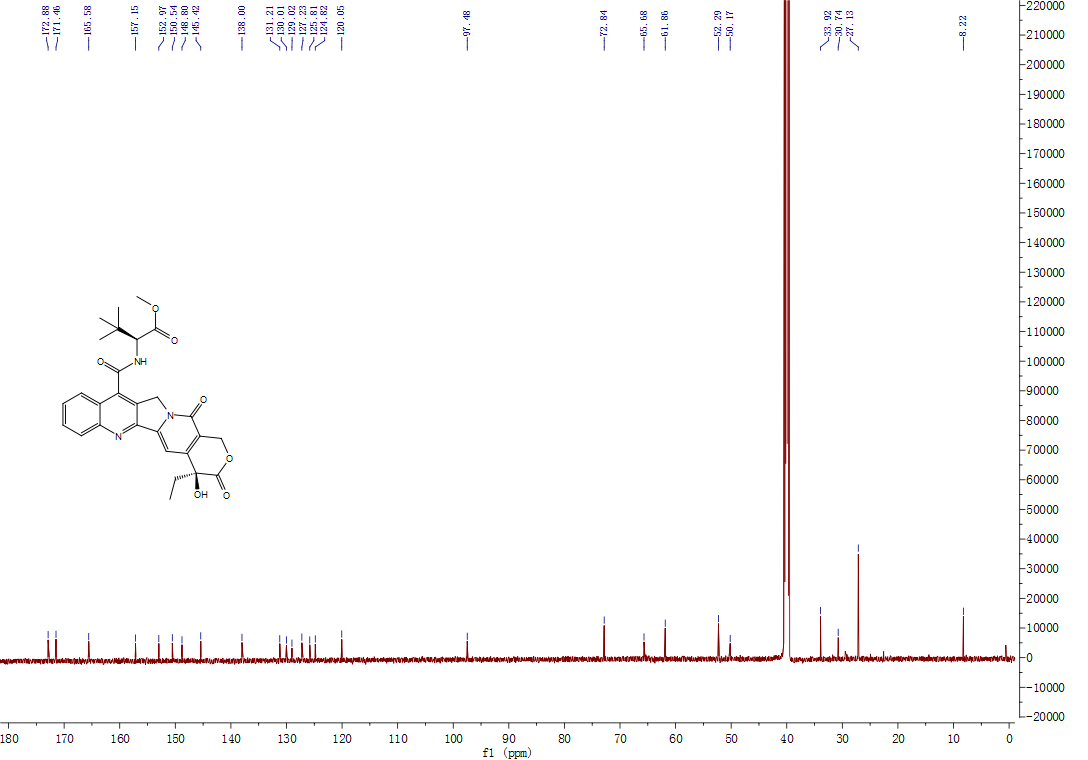
**

**Supplementary Figure 36**. ^13^C NMR spectrum of **4p**


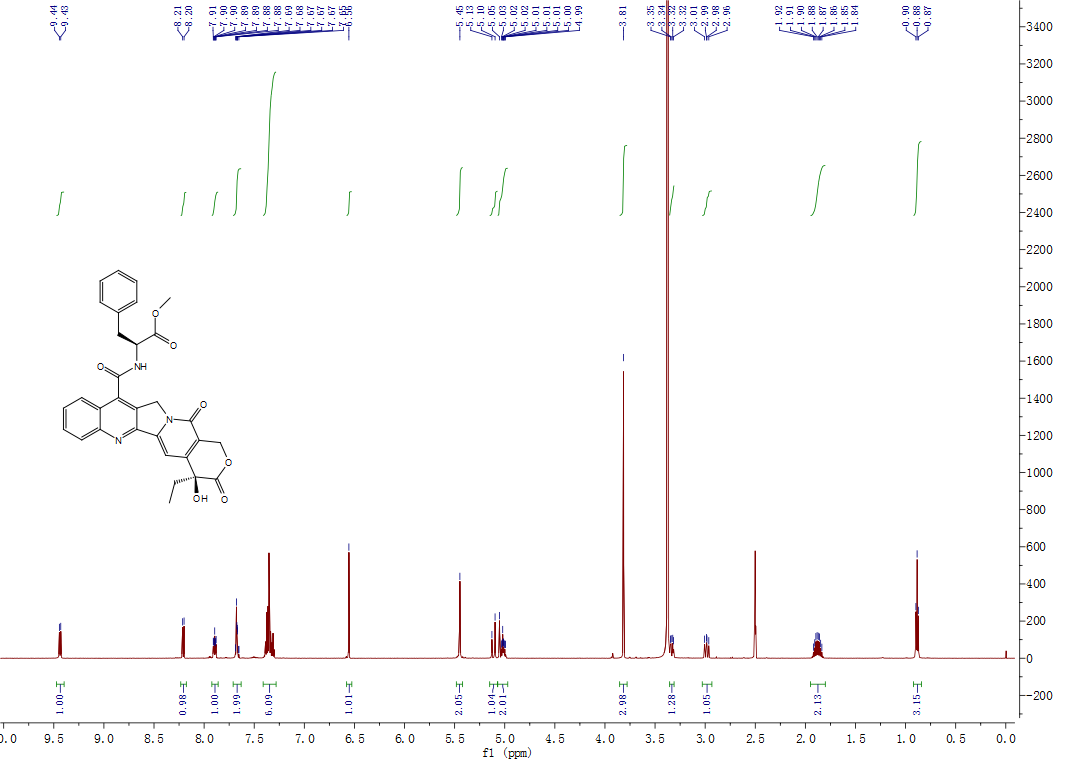


**Supplementary Figure 37**. ^1^H NMR spectrum of **4q**


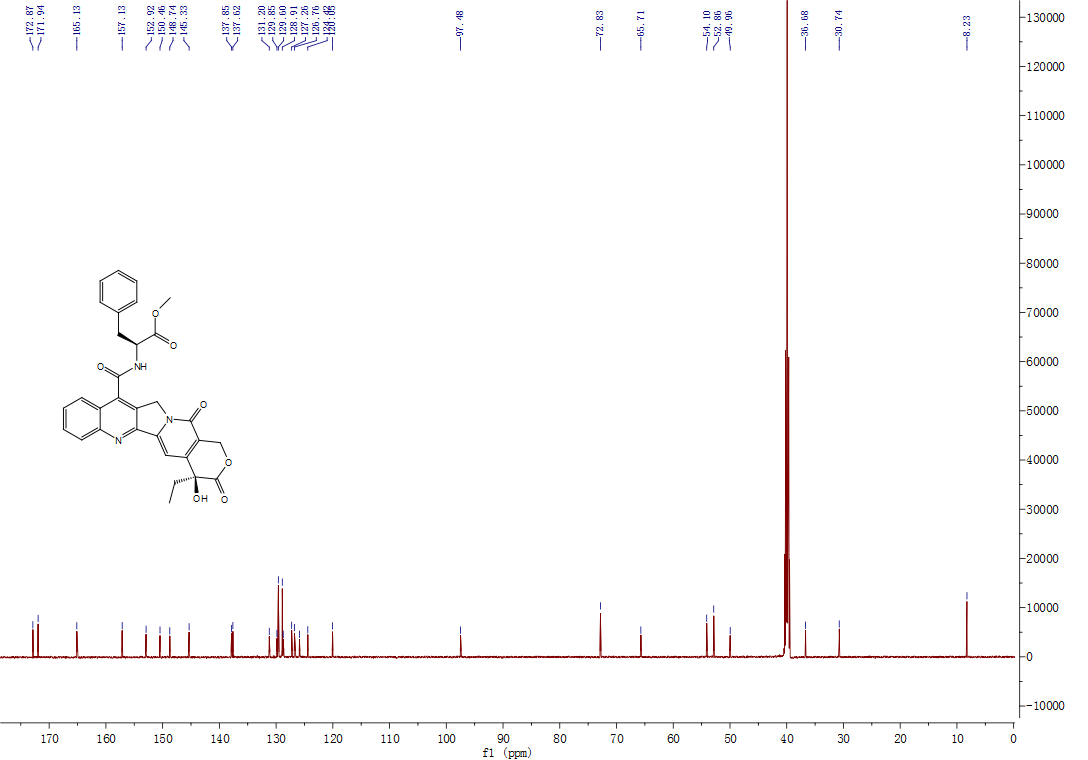


**Supplementary Figure 38**. ^13^C NMR spectrum of **4q**
